# Global, regional and time-trend prevalence of central obesity: a systematic review and meta-analysis of 13.2 million subjects

**DOI:** 10.1007/s10654-020-00650-3

**Published:** 2020-05-24

**Authors:** Martin C. S. Wong, Junjie Huang, Jingxuan Wang, Paul S. F. Chan, Veeleah Lok, Xiao Chen, Colette Leung, Harry H. X. Wang, Xiang Qian Lao, Zhi-Jie Zheng

**Affiliations:** 1 Jockey Club School of Public Health and Primary Care, Faculty of Medicine, Chinese University of Hong Kong, 4/F, School of Public Health, Prince of Wales Hospital, Hong Kong, Hong Kong; 2 School of Public Health, Sun Yat-Sen University, Guangzhou, China; 3 Department of Global Health, School of Public Health, Peking University, 38, Xue Yuan Road, Haidian District, Beijing, China

**Keywords:** Central obesity, Waist circumference, Prevalence, Epidemiology, Temporal trend

## Abstract

We aimed to examine the global prevalences of central obesity according to age, sex, race, place of residence, geographical region, national income level, and the definitions of central obesity. MEDLINE and Embase were searched. Studies with sample size of ≥ 500 and investigated individuals aged ≥ 15 years were included. Metaprop (a Stata command) was adopted to conduct a meta-analysis of prevalence, and the Freeman-Tukey Double Arcsine Transformation was used to stabilize the variances. A random-effects model was used to evaluate the prevalence and 95% confidence intervals (CI) of central obesity. There were 288 studies involving 13,233,675 individuals in this analysis. The overall prevalence of central obesity was 41.5% (95% CI 39.9–43.2%). A higher prevalence was found in older individuals, female subjects, urban residents, Caucasians, and populations of higher income level countries. Regarding regional variations, the highest prevalence was found in Sothern America (55.1%, 95% CI 45.8–64.3%) and Central American (52.9%, 95% CI 32.7–72.7%). Its prevalence was rapidly rising from 1985 to 2014. From 1985–1999 to 2010–2014, younger subjects aged 15–40 years showed a more drastic rise in prevalence (16.3 to 33.9%) than subjects aged > 40 years (43.6 to 57.9%). Male individuals have a more drastic rise (25.3 to 41.6%) than females (38.6 to 49.7%). Major increasing in prevalence of the condition in the past three decades, particularly in certain subgroups. These findings could act as a useful reference to inform public health strategies to minimize the impact of central obesity on population health.

**Electronic supplementary material:**

The online version of this article (10.1007/s10654-020-00650-3) contains supplementary material, which is available to authorized users.

## Introduction

Central obesity is defined by the World Health Organization defined central obesity as a waist circumference (WC) of greater than 94 cm and 80 cm for males and females, respectively. The International Diabetes Federation proposed different cut-off points for different ethnic groups (e.g. 94 cm for males and 80 cm for females for Europeans, 90 cm for males and 80 cm for females for Asians) [1]. The prevalence of central obesity is rising globally due to a combination of physical inactivity and consumption of unhealthy diet [2]. This has contributed significantly to increased financial burden [3] and avoidable utilization of the healthcare system [4].

Central obesity could contribute to an increased risk of many medical conditions, including cardiovascular diseases (CVD)[5], stroke [6], type 2 diabetes mellitus[7], hypertension [8], various types of cancer (e.g. colorectum, pancreas, endometrium, and breast) and all-cause mortality [9]. It is also associated with other co-morbidities, such as dyslipidemia, hip fracture [10], and depression. Some studies supported WC as a better predictor for CVD than body mass index (BMI). A recent study reported that WC had a higher relative integrated discrimination index than BMI in both men (6.9% versus 3.2%) and women (9.6% versus 9.2%) [11]. Although total cholesterol, HDL-C, hypertension, and diabetes appeared to mediate the risk incurred by central obesity, the association between WC and CVD risk remained significant after controlling for these factors. This indicated that central obesity may play an important role in the primary prevention of CVD.

Determining the regional and time-trend prevalence of central obesity is important as it may inform resource allocation and policy making to reduce its disease burden through health education, screening and early intervention. However, the reported prevalence of central obesity in existing literature may be affected by the research settings (e.g. geographical region, place of residence, and study time period), the characteristics of the population groups studied (e.g. age range, gender proportion and race), sampling methods, diagnostic criteria, and other factors. A systematic review is required to obtain a comprehensive examination of central obesity as a global disease burden. As there is no such previous study identified, we aim to perform a systematic review and meta-analysis of the worldwide and time-trend prevalence of central obesity.

## Methods

### Search strategy and selection criteria

MEDLINE and Embase were searched for population-based, epidemiological studies reporting the prevalence of central obesity from our previous database of metabolic syndrome. A pre-determined search strategy (Supplementary Table 1) was used to search literature according to the quality of reporting of the MOOSE (Meta-analyses Of Observational Studies in Epidemiology) guidelines (Supplementary Table 2) [12].

A multidisciplinary group conducted the meta-analysis led by MCSW with JH, JW, PSFC, and VL as reviewers. Consensus was reached by referral to a third reviewer (XC) when there was disagreement. All returned citations were screened by title and abstract first, followed by full texts if relevant. The pre-determined criteria in the initial screening stage were studies (1) investigating the prevalence of central obesity; (2) using observational design; (3) reporting original data. Citations remained were eligible for full-text screening. Only population-based studies were reviewed, and they were defined as those that involved all residents in a specific region as the sampling frame based on a sampling method that was representative of that region. Additionally, the studies should: (1) have a sample size of no less than 500 participants; (2) have investigated individuals aged no less than 15 years; (3) adopt clinical approaches of Adult Treatment Panel (ATP III), International Diabetes Federation (IDF), World Health Organization (WHO) or Joint Interim Statement (JIS) to assess central obesity; and (4) contain sufficient information to calculate the number of individuals with central obesity. If there were citations based on the same study, the one reporting the most detailed information was used.

### Data extraction and quality assessment

Basic information collected from the individual studies consists of the name of first author; year of publication; study time period (the period in which the study was performed); country and region of recruitment (urban vs. rural); age range; sex; as well as ethnicity of the study participants. Relevant information was extracted to estimate prevalence, and the data retrieved included definitions of central obesity; sample size and case numbers; and crude/age-specified/sex-specified prevalence rate. Different criteria of central obesity were shown in Supplementary Table 3. The classification of regions including income group were clarified in Supplementary Table 4. Two reviewers (PSFC, JW) independently evaluated the quality of each included citation using the modified Newcastle–Ottawa-Quality Assessment Scale (NOS) [13].

### Statistical analysis

A systematic, analytical method was used to compute the pooled prevalence rate of central obesity from all included studies. A Stata command, “metaprop”, was adopted to conduct meta-analysis of rates to generate pooled estimates with exact binomial and score test-based confidence intervals (CIs) [14]. The method provided appropriate ways of combining rates close to the margins by using the Freeman–Tukey Double Arcsine Transformation to stabilize the variances. A random-effects model was used to pool the prevalence of central obesity with proportions and 95% CIs. Heterogeneity across different studies was calculated using Cochran’s Q test and chi square statistics. Subgroup analysis by age, sex, race, place of residence, geographical region, national income level (according to World Bank Income Group in 2017), and definition of central obesity were conducted to explore the observed heterogeneity. Temporal trends of the prevalence of central obesity in different age and sex groups were investigated by subgroup analysis. A choropleth map, which used differences in shading to indicate the prevalence rate of central obesity in different countries, was generated for the overall estimation of prevalence rates. Univariate and multivariate meta-regression analysis were conducted to identify potential effects of confounders or modifiers. Sensitivity analysis was performed by omitting one study at a time, generating the pooled estimates and comparing with the original estimates to examine the stability of the results. A choropleth map was generated for the overall estimated prevalence of central obesity in individual countries. The statistical analysis was conducted using *Stata* version 14.0 (College Station, Texas). The graphic compositions were performed by *R* version 3.3.2 (R Core Team).

## Results

**Fig. 1:**
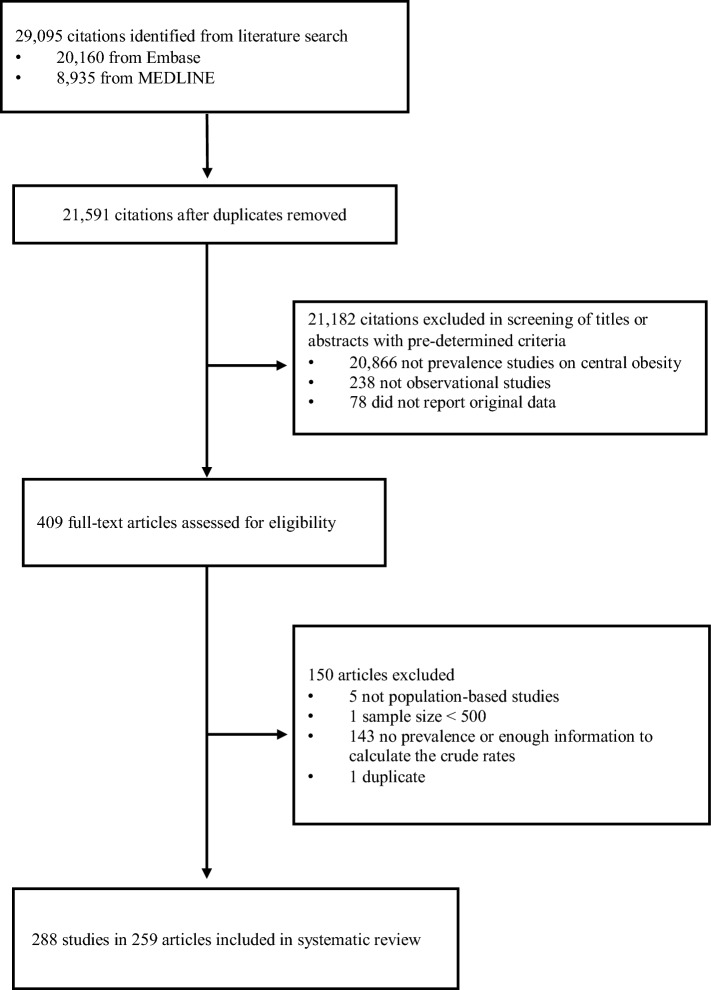
Selection of articles for systematic review

In the literature search, 29,095 citations were identified, of which 20,160 were from Embase and 8,935 were from MEDLINE (Fig. 1). There were 21,591 citations after removal of duplicates. We retrieved 409 full-text articles assessed for eligibility after 21,182 citations were excluded during title or abstract screening as they did not investigate the prevalence of central obesity, use an observational design, or report original data. We excluded 150 articles as they were not population-based; the sample size was smaller than 500; there were no sufficient information to calculate the crude prevalence; or due to duplicate data source. Some publications recruited subjects with more than one ethnicity; or were performed across decades. We considered them as articles containing multiple studies, and more than one prevalence rates were extracted in this type of publication. Finally, there were 288 studies in 259 articles included in the present meta-analysis.

The characteristics of all eligible studies were shown in Supplementary Tables 5, and the quality of these studies as assessed by the NOS was presented in Supplementary Table 6. Among 259 citations, 174 citations (67.2%) were judged to have high quality in both patient definition and data representativeness. Prevalence figures were presented for Western Europe (11 studies), Northern Europe (10), Eastern Europe (10), Southern Europe (21), Eastern Asia (100), Western Asia (25), Southern-Central Asia (30), South-Eastern Asia (8), Northern America (31), Central America (5), Southern America (21), and Africa (16). Trained staffs were recruited to measure the waist circumference in 273 studies (94.8%) while other studies did not mention how the data were collected (n = 15, 5.2%).

**Fig. 2:**
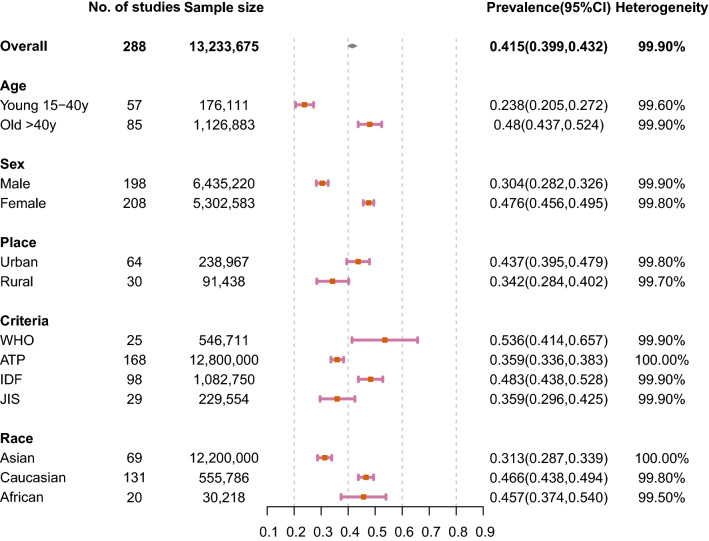
Prevalence of central obesity by age, sex, place, criteria, and race

**Fig. 3:**
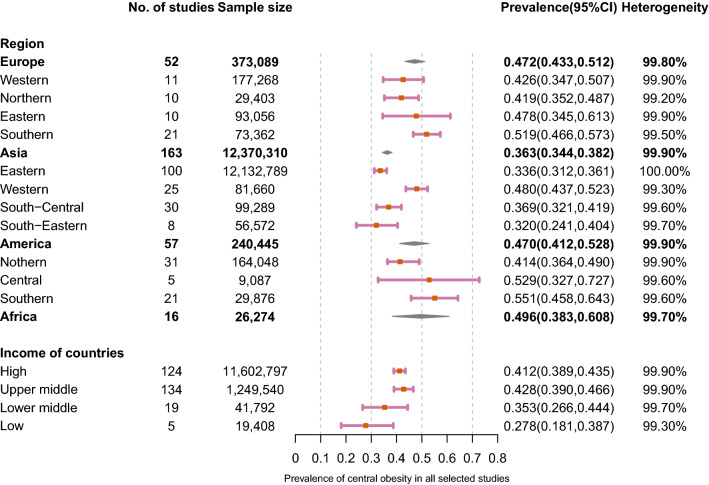
Prevalence of central obesity by region and income level

**Fig. 4:**
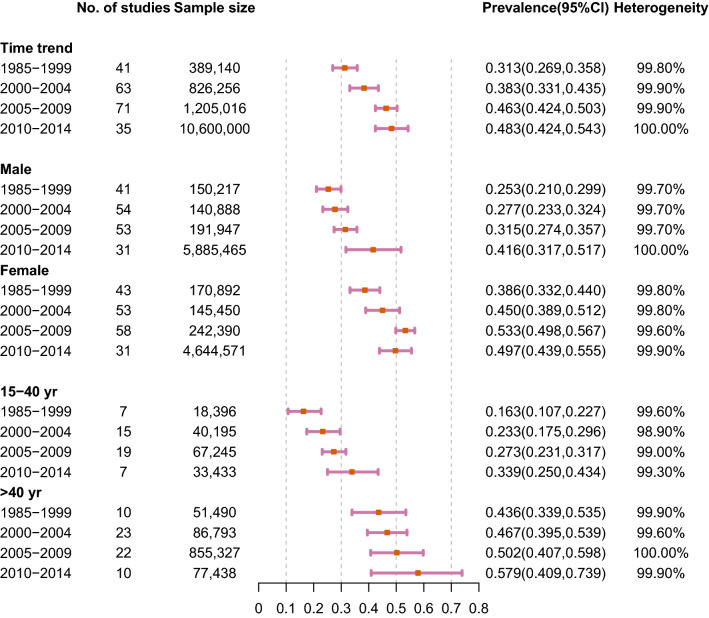
Time trend prevalence of central obesity by sex and age

The results of meta-analysis were presented in Figs. 2, 3 and 4. The overall prevalence of central obesity in 288 selected studies involving 13.2 million individuals was 41.5% (95% CI 39.9%-43.2%; I^2^ 99.9%). Time trend was classified by 4 periods (i.e. 1985–1999, 2000–2004, 2005–2009 and 2010–2014, Fig. 4). We stratified the study period into four groups, taking a balance between the number of groups and sample size within each group into consideration. An increasing trend of prevalence was observed across time periods. The prevalence of each period was 31.3% (1985–1999, 95% CI 26.9–35.0.8%; I^2^ 99.8%), 38.3% (2000–2004, 95% CI 33.1–43.5%; I^2^ 99.9%), 46.3% (2005–2009, 95% CI 42.4–50.3%; I^2^ 99.9%) and 48.3% (2010–2014, 95% CI 42.4–54.3%; I^2^ 100.0%), respectively. Regarding age-specific prevalence, the older group (age > 40 years) (48.0%, 95% CI 43.7–52.4%; I^2^ 99.9%) had a higher prevalence than the younger group (15–40 years) (23.8%, 95% CI 20.5–27.2%; I^2^ 99.6%). Besides, the overall prevalence was higher in female (47.6%, 95% CI 45.6–49.5%; I^2^ 99.8%) than male individuals (30.4%, 95% CI 28.2–32.6%; I^2^ 99.9%).

When the time trend was analysed by different sex and age groups, an even sharper increase was observed among young adults and male subjects than other subgroups (Fig. 4). Among adults aged 15–40 years, the prevalence increased from 16.3% (95% CI 10.7%-22.7%; I^2^ 99.6%) during 1985–1999 to 33.9% (95% CI 25.0–43.4%; I^2^ 99.3%) during 2010–2014. For adults aged > 40 years, it increased from 43.6% (95% CI 33.9–53.5%; I^2^ 99.9%) during 1985–1999 to 57.9% (95% CI 40.9–73.9%; I^2^ 99.9%) during 2010–2014. We chose the age of 40 years as a cut-off due to this value was adopted in more primary studies. For male individuals, the prevalence increased from 25.3% (95% CI 21.0–29.9%; I^2^ 99.7%) during 1985–1999 to 41.6% (95% CI 31.7–51.7%, I^2^ = 100.0%) during 2010–2014. For female adults, it increased from 38.6% (95% CI 33.2–44.0%; I^2^ 99.8%) during 1985–1999 to 49.7% (95% CI 43.9–55.5%, I^2^ 99.9%) during 2010–2014.

Individuals living in urban regions (43.7%, 95% CI 39.5–47.9%; I^2^ 99.8%) had a higher prevalence than individuals living in rural regions (34.2%, 95% CI 28.4–40.2%; I^2^ 99.7%) (Fig. 2**)**. Diagnostic criteria using WHO, ATP, IDF and JIS are the most common used. The prevalence of central obesity based on WHO is 53.6% (95% CI 41.4–65.7%; I^2^ 99.9%), ATP is 35.9% (95% CI 33.6–38.3%; I^2^ 100.0%), IDF is 48.3% (95% CI 43.8–52.8%; I^2^ 99.9%) and JIS is 35.9% (95% CI 29.6–42.5%; I^2^ 99.9%). When compared with Caucasian (46.6%, 95% CI 43.8–49.4%; I^2^ 99.8%) and African (45.7%, 95% CI 37.4–54.0%; I^2^ 99.5%) population, Asian people (31.3%, 95% CI 28.7–33.9%; I^2^ 100.0%) were reported to have lower prevalence. Regarding regional variations, the highest prevalence was found in Sothern America (55.1%, 95% CI 45.8–64.3%; I^2^ 99.6%) and Central American (52.9%, 95% CI 32.7–72.7%; I^2^ 99.6%) followed by Southern Europe (51.9%, 95% CI 46.6–57.3%; I^2^ 99.5%) (Fig. 3). The highest prevalence in Asian regions was found in Western Asia (48.0%, 95% CI 43.7–52.3%; I^2^ 99.3%). The prevalence in Africa was 49.6% (95% CI 38.3%-60.8%; I^2^ 99.7%). The prevalence was higher in high-income (41.2%, 95% CI 38.9–43.5%; I^2^ 99.9%) than low-income countries (27.8%, 95% CI 18.1–38.7%; I^2^ 99.3%).

**Fig. 5:**
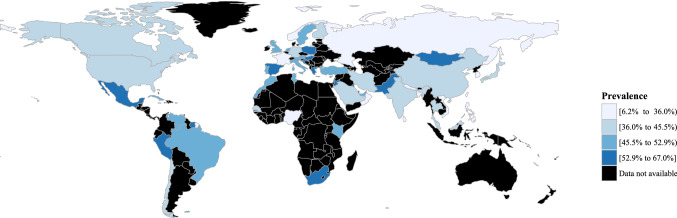
Global prevalence of central obesity by country

A choropleth map for the prevalence of central obesity indicates substantial variations across different countries (Fig. 5). Regions with the highest prevalence (over 55%) include Hungary (67.0%), Peru (64.7%), Kuwait (64.5%), Mongolia (64.3%), Qatar (62.0%), Pakistan (61.8%), South Africa (58.4.5%), Jordan (58.4%), Poland (57.9%), Suriname (57.4%), Greece (56.8%), and Croatia (56.5%). Regions with the lowest prevalence (below 30%) include Nigeria (6.2%), Bangladesh (15.3%), Vietnam (15.4%), Sri Lanka (16.2%), Taiwan (18.7%), Philippines (22.4%), Nepal (22.9%), France (25.9%), and Jamaica (29.2%).

We performed a meta-regression analysis to investigate study-level factors that may impact the estimation on prevalence of central obesity and contribute to heterogeneity across studies (Supplementary Table 7). The results showed that the estimation on prevalence of central obesity was not substantially altered by region (*p* = 0.071–0.779), criteria (*p* = 0.099–0.849), measurement method (*p* = 0.657), or study quality (*p* = 0.330). In the univariate meta-regression analysis age (*p* = 0.007), region (*p* = 0.042), place (*p* < 0.001), period (*p* < 0.001), and study quality (*p* = 0.042) were associated with the heterogeneity observed. However, in the multivariate regression analysis, only age (*p* = 0.001), place (*p* < 0.001), and period (*p* < 0.001) remained to be the sources of heterogeneity. It was found that the prevalence of central obesity was higher in studies on older individuals (median age ≥ 55 years vs < 55 years, adjusted risk ratio (ARR) 1.23, 95% CI 1.09–1.38), residents in urban regions (urban vs rural, ARR 1.70, 95% CI 1.31–2.19), and the more recent decade (2005–2014 vs 1980–2004, ARR 1.34, 95% CI 1.17–1.53).

The sensitivity analysis shows that the results of the overall prevalence estimation were not affected by excluding any single study, indicating the stability and robustness of the results (Supplementary Table 8).

## Discussion

This systematic review provided a comprehensive and up-to-date analysis on the global prevalence of central obesity by different sociodemographic characteristics, its various diagnostic criteria, national income of countries where the study subjects were living in; as well as its temporal tend by age and sex, respectively. We found a rising trend in overall prevalence since the 1990s and this trend was more drastic in young adults and male individuals. A higher prevalence was found in older subjects, females, urban regions, Caucasians, and people from higher income countries.

The overall prevalence of central obesity was estimated to be 41.5% (95% C.I. 39.9–43.2%), with an obvious increase since the 1990s from 31.3% (1985–1999) to 48.3% (2010–2014). This increasing trend of prevalence in central obesity could be attributed to economic development and urbanization which could lead to an unfavourable change in dietary habits (consumption of high-calorie foods and sweetened beverages) [15], physical inactivity [16], sedentary behaviours (smartphone use, computer use, TV or video viewing), stress and cortisol secretion [17]. The global dietary habits have changed in obvious ways in the last four decades. Dietary habits in the population have transformed to a greater intake of processed and over-processed meats and drinks that are lacking nutrients and excess in calorie [18]. The over intake of processed food has also been associated with lower consumption of some healthy nutrient components, including white meat, legumes, coarse grains, fruits and other vegetables [19]. The consumption of sodium, fats, and sugars did not meet the levels recommended by guideline in many regions [20, 21]. Decreases in physical activity are continuously popular over the past four decades [22] and may have contributed to the increased prevalence in central obesity. Industrialization has resulted in some risk factors, including air pollution, heavy traffic transportation, high-density buildings, limited resources of green space and sports facilities, and violence, which may have caused some barriers to the access of physical activity [23]. The current low levels of physical activity are attributed to the less involvement in physical activities and an increasing trend of sedentary behaviour in the working and domestic environments [24]. The WHO has made an agreement on a voluntary global non-communicable diseases programme goal for an increase of 10% in physical activity by 2025 [25].

The prevalence of central obesity among individuals aged > 40 were around twice as much as that in younger adults aged 15–40 (48.0 vs. 23.8%). This is well understood by researchers as the basal metabolic rate is lower in old adults than in young adults [26], leading to excess fat stored in the body due to an increase in energy intake: energy expenditure ratio [27]. Another reason could be older individuals may not be as physically active as young adults and have lower energy expenditure [49]. Despite the above facts, it is remarkable that the prevalence of central obesity among young adults increased by twice as much as that in older subjects from 1985–1999 to 2010–2014. This could be attributed to more individuals, such as children and adolescents, are having central obesity at an earlier stage of their life [28, 29]. This trend has been speculated to rise continuously in the future given the lifelong exposure and with inevitable ageing. Obesity may be associated with higher risks of complications, including diabetes, hypertension, lipid disorders, and cancers in different ages [30–33]. A study found age at onset of obesity was negatively associated with risk of diabetes [34]. Similarly, another study concluded the mortality decreased with onset age of obesity. They observed the lowest mortality in subjects with obesity onset at > 50 years but the highest mortality in people with obesity onset at 18 to 29 years [35]. We have also conducted an analysis of the epidemiology of colorectal cancer from 39 countries, and found that the incidence of colorectal cancer continued to increase in younger populations. This may be partly explained by the increasing prevalence of central obesity among younger individuals [36]. Therefore, the implications for medical professionals and supporting agencies are to encourage the prevention of central obesity, weight loss and more physical activity in the younger population. Besides, attention need to be paid on early detecting, closely monitoring and positively reversing metabolic syndrome for all patients, especially those in younger age. Aging is also associated with progressive transitions in fat distribution, which refers to the increase in abdominal fat combined with a decrease in lower body subcutaneous fat. This age-related fat redistribution is associated with an increased risk of morbidity and mortality. However, the impacts of lifestyle risk factors on fat redistribution remained unknown [37].

The global prevalence of central obesity was estimated to be higher among females when compared with males (47.6 vs. 30.4%, respectively). In additional to the biological differences where females generally have higher body fat proportion than that of males and need more essential fat, cultural factors and social restrictions may also explain this gender difference. Women generally have less physical activity because of a lower education level, sedentary lifestyle and a higher level of housework engagement [38, 39]. Also, sex hormones and the effect of menopause might explain the differences in prevalence between males and females [40, 41]. Central obesity seems to be associated with low levels of testosterone as the hormone promotes fat consumption and decrease central obesity [42–45]. The high prevalence of central obesity in females implicates the importance to implement healthy lifestyle modification strategies for this group. Although the prevalence of central obesity was higher in females in both periods, the increasing rates of central obesity were higher in males (from 25.3 to 41.6%) compared with females (from 38.6 to 49.7%). This may be related to the difference in the prevalence trend of lifestyle risk factors by genders. The prevalence of males (50.0%) smoking was three times the prevalence of users among females (16.7%) in 2000. By 2015, the prevalence for males (40.3%) was more than four times the prevalence for females (9.5%). It was estimated that the prevalence for males (35.1%) is five times the prevalence for females (6.7%) by 2025 [46]. In terms of alcohol consumption, its global adult per-capita had increased from 5.9 to 6.5 L in the past three decades and is expected to grow to 7.6 L in the next decade [47]. The sex ratios (males/ females) for heavy episodic drinking have increased from 2.3 in 2000 to 2.5 in 2016 [48]. As for physical activity, It was found by a recent analysis that females were less active than males and the prevalence of female physical activity has not increased since 2001 [49]. Therefore, the prevalence of central obesity among females is expected to rise further and contribute to a more substantial disease burden. This represents a target group where preventive measures and clinical management should be strengthened. Evidence-based approaches are needed to enhance its early detection, formulate lifestyle modification strategies, and devise evidence-based guidelines in order to mitigate its rising trends and ameliorate its associated morbidity and mortality.

The prevalence of central obesity was 44.7% in high-income and 43.6% in upper-middle income countries, compared with 30.1% in lower-middle income and 30.6% in low-income countries. Economic development is associated with a high risk of obesity [50]. Economic theory suggested that obesity may be attributed to scientific and technological advances which had altered people’s dietary habits, lifestyles and work patterns [51]. This may also be applied to central obesity. Previous studies have discussed a dose–response association between exposure to work stress and the development of central obesity [52], which possibly induce central obesity through dysregulation of the hypothalamic–pituitary–adrenal axis [53]. The high prevalence of central obesity in upper-middle-income countries and high-income countries highlights the importance for physicians and policymakers to implement health promotion strategies for their patients and the general population. As the economy grows, the prevalence of central obesity in the underdeveloped regions is expected to increase further. There is a rising trend in the developing regions, where the economic and lifestyle transition imposes more constraints on facing the double burden of communicable and non-communicable diseases (NCDs) in an underprivileged environment, characterized by poor health systems [54]. It was reported that NCDs resulted in more than half of all mortality in low- and middle-income nations (29 million deaths annually).[55]. NCDs are increasing in all over the world but most rapidly in the region of sub-Saharan Africa, where the estimated rise in NCDs will outpace the decrease in communicable diseases [56].

### Study limitations

This study was the first comprehensive meta-analysis that examined the global prevalence of central obesity taking sociodemographic variables, peoples’ races, geographical regions, and national income into consideration. It has a large sample size of over 13 million subjects. However, several limitations should be addressed. Firstly, the degree of heterogeneity in this study was large (*I*^2^ > 98%). The included studies had a long-time span (from 1985 to 2014); adopted different sampling methods; and possessed a huge between-study variation in sample size and age range. Nevertheless, a pervious study indicated that the measurement of heterogeneity by *I*^2^ can be influenced by large sample size and could be large (> 75%),[57], and any amount of heterogeneity is acceptable if both accurate data and predefined eligibility criteria were provided [58]. In addition, inconsistent criteria used in different studies made direct comparison of findings challenging, yet additional sensitivity analysis had showed the distribution of definitions used was mostly balanced between groups. Also, it is difficult to compare the prevalence trend across countries and populations at different times. These weaknesses may have limited the generalizability of the results to a particular region or population.

## Conclusions

In summary, this meta-analysis estimated the global prevalence of central obesity in the past few decades using more than 280 population-based studies worldwide. The estimated global prevalence is 41.5% among individuals aged 15 or above. Higher prevalence was found in individuals aged > 40 years, females, people living in urban regions, Caucasian and African population, and residents in high-income countries. We also identified an increase in the overall prevalence, and a more drastic increase in younger subjects and male subjects according to temporal trend. Therefore, it is of vital importance for the government and health organizations to devise and implement preventive measures and early interventions to tackle its rising threat. Besides the general population, measures at individual and public health levels targeted at the high-risk groups (e.g. Caucasian or African older adults and females) may represent cost-effective means to control its growing burden. However, the measurements should be tailored for individual country or population as the epidemiology of central obesity could vary between countries or populations.

## Author Contributors

MCSW, JH, JW, and ZJZ participated in study concept and design. MCSW, JH, JW, PSFC, VL, XC, CL and ZJZ participated in acquisition, analysis, or interpretation of data. MCSW and JH drafted the manuscript. All authors, including HHXW and XQL, provided critical revision of the manuscript for important intellectual content. All authors read and approved the final manuscript. All authors included on a paper fulfil the criteria of authorship. There is no one else who fulfils the criteria but has not been included as an author.

## Electronic supplementary material

Below is the link to the electronic supplementary material.

Supplementary file1 (DOCX 277 kb)
