# Supplementary material for: Global, regional and time-trend prevalence of central obesity: a systematic review and meta-analysis of 13.2 million subjects
Source: Eur J Epidemiol. 2020 May 24;35(7):673–83. doi: 10.1007/s10654-020-00650-3 (PMC7387368; doi:10.1007/s10654-020-00650-3)
Supplement: Supplementary file 1 — Supplementary file1 (DOCX 277 kb) [file 10654_2020_650_MOESM1_ESM.docx]

| **Supplementary Files**  **Supplementary Table 1** Search strategy for article selection  **Supplementary Table 2** MOOSE Checklist for Meta-analyses of Observational Studies  **Supplementary Table 3** Different definitions of metabolic syndrome  **Supplementary Table 4** Classifications of regions  **Supplementary Table 5** Characteristics of studies on prevalence  **Supplementary Table 6** Quality assessment of selected articles  **Supplementary Table 7** Results of Univariate and Multivariate Meta-regression Analysis  **Supplementary Table 8** Results of sensitivity analysis  **Supplementary Table 1. Search strategy for article selection**    **Supplementary Table 2. MOOSE Checklist for Meta-analyses of Observational Studies**   \| **Item No** \| **Recommendation** \| **Reported on Page No** \| \| --- \| --- \| --- \| \| Reporting of background should include \| \| \| \| 1 \| Problem definition \| 4 \| \| 2 \| Hypothesis statement \| 5 \| \| 3 \| Description of study outcome(s) \| 6 \| \| 4 \| Type of exposure or intervention used \| 6 \| \| 5 \| Type of study designs used \| 6 \| \| 6 \| Study population \| 6 \| \| Reporting of search strategy should include \| \| \| \| 7 \| Qualifications of searchers (eg, librarians and investigators) \| 6 \| \| 8 \| Search strategy, including time period included in the synthesis and key words \| 5 \| \| 9 \| Effort to include all available studies, including contact with authors \| 5 \| \| 10 \| Databases and registries searched \| 5 \| \| 11 \| Search software used, name and version, including special features used (eg, explosion) \| 5 \| \| 12 \| Use of hand searching (eg, reference lists of obtained articles) \| 5 \| \| 13 \| List of citations located and those excluded, including justification \| 7 \| \| 14 \| Method of addressing articles published in languages other than English \| 6 \| \| 15 \| Method of handling abstracts and unpublished studies \| 6 \| \| 16 \| Description of any contact with authors \| NA \| \| Reporting of methods should include \| \| \| \| 17 \| Description of relevance or appropriateness of studies assembled for assessing the hypothesis to be tested \| 6 \| \| 18 \| Rationale for the selection and coding of data (eg, sound clinical principles or convenience) \| 6 \| \| 19 \| Documentation of how data were classified and coded (eg, multiple raters, blinding and interrater reliability) \|  \| \| 20 \| Assessment of confounding (eg, comparability of cases and controls in studies where appropriate) \| 6 \| \| 21 \| Assessment of study quality, including blinding of quality assessors, stratification or regression on possible predictors of study results \| 6 \| \| 22 \| Assessment of heterogeneity \| 7 \| \| 23 \| Description of statistical methods (eg, complete description of fixed or random effects models, justification of whether the chosen models account for predictors of study results, dose-response models, or cumulative meta-analysis) in sufficient detail to be replicated \| 6 \| \| 24 \| Provision of appropriate tables and graphics \| 17 \| \| Reporting of results should include \| \| \| \| 25 \| Graphic summarizing individual study estimates and overall estimate \| 19 \| \| 26 \| Table giving descriptive information for each study included \| 24 \| \| 27 \| Results of sensitivity testing (eg, subgroup analysis) \| 19 \| \| 28 \| Indication of statistical uncertainty of findings \| 19 \| \| Reporting of discussion should include \| \| \| \| 29 \| Quantitative assessment of bias (eg, publication bias) \| NA \| \| 30 \| Justification for exclusion (eg, exclusion of non-English language citations) \| 7 \| \| 31 \| Assessment of quality of included studies \| 8 \| \| Reporting of conclusions should include \| \| \| \| 32 \| Consideration of alternative explanations for observed results \| 11 \| \| 33 \| Generalization of the conclusions (ie, appropriate for the data presented and within the domain of the literature review) \| 13 \| \| 34 \| Guidelines for future research \| 13 \| \| 35 \| Disclosure of funding source \| 2 \| |
| --- | --- | --- | --- | --- | --- | --- | --- | --- | --- | --- | --- | --- | --- | --- | --- | --- | --- | --- | --- | --- | --- | --- | --- | --- | --- | --- | --- | --- | --- | --- | --- | --- | --- | --- | --- | --- | --- | --- | --- | --- | --- | --- | --- | --- | --- | --- | --- | --- | --- | --- | --- | --- | --- | --- | --- | --- | --- | --- | --- | --- | --- | --- | --- | --- | --- | --- | --- | --- | --- | --- | --- | --- | --- | --- | --- | --- | --- | --- | --- | --- | --- | --- | --- | --- | --- | --- | --- | --- | --- | --- | --- | --- | --- | --- | --- | --- | --- | --- | --- | --- | --- | --- | --- | --- | --- | --- | --- | --- | --- | --- | --- | --- | --- | --- | --- | --- | --- | --- | --- | --- | --- | --- | --- | --- | --- | --- |


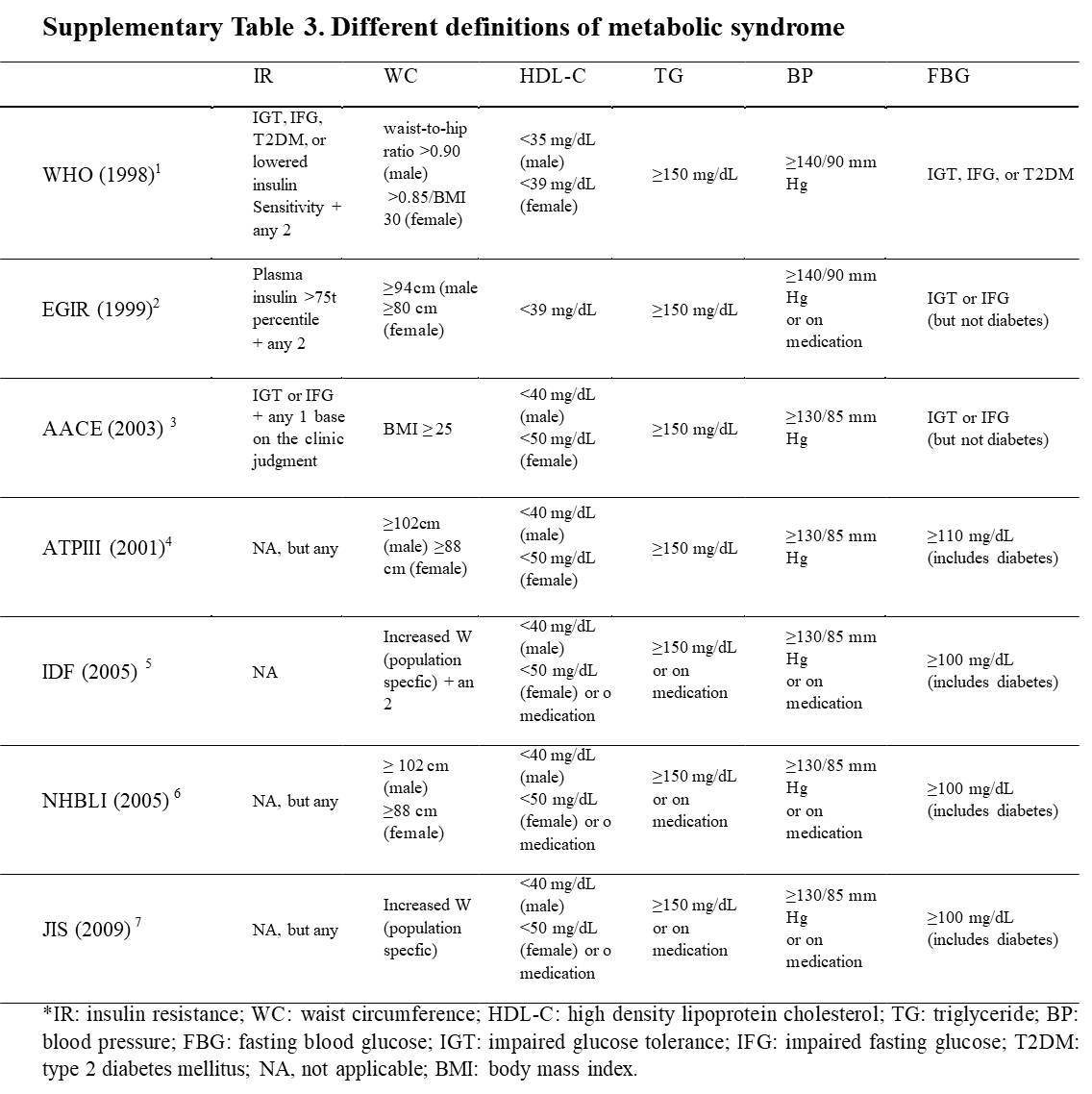


**Table reference:**

1. K. G. Alberti and P. Z. Zimmet, “Definition, diagnosis and classification of diabetes mellitus and its complications Part 1: diagnosis and classification of diabetes mellitus provisional report of aWHO consultation,” DiabeticMedicine, vol. 15, no. 7, pp. 539–553, 1998.
2. B. Balkau and M. A. Charles, “Comment on the provisional report from the WHO consultation: European Group fo the Study of Insulin Resistance (EGIR),” Diabetic Medicine, vol. 16, no. 5, pp. 442–443, 1999.
3. D. Einhorn,G.M. Reaven, R. H. Cobin et al., “American College of Endocrinology position statement on the insuli resistance syndrome,” Endocrine Practice, vol. 9, no. 3, pp. 237–252, 2003.
4. J. I. Cleeman, “Executive summary of the third report of the National Cholesterol Education Program (NCEP) expert panel on detection, evaluation, and treatment of high blood cholesterol in adults (adult treatment panel III),” Journal of the American Medical Association, vol. 285, no. 19, pp. 2486–2497, 2001.
5. Alberti, K. G., Zimmet, P., Shaw, J. & Group, I. D. F. E. T. F. C. The metabolic syndrome--a new worldwide definition. *Lancet* **366**, 1059-1062, doi:10.1016/S0140-6736(05)67402-8 (2005).
6. Grundy, S. M. *et al.* Diagnosis and management of the metabolic syndrome: an American Heart Association/National Heart, Lung, and Blood Institute Scientific Statement. *Circulation* **112**, 2735-2752, doi:10.1161/CIRCULATIONAHA.105.169404 (2005).
7. Alberti, K. G. *et al.* Harmonizing the metabolic syndrome: a joint interim statement of the International Diabetes Federation Task Force on Epidemiology and Prevention; National Heart, Lung, and Blood Institute; American Heart Association; World Heart Federation; International Atherosclerosis Society; and International Association for the Study of Obesity. *Circulation* **120**, 1640-1645, (2009).

**Supplementary Table 4. Classifications of regions**

| **Country Name** | **UN sub-region** | **UNICEF region^1^** | **WHO Region^2^** | **MDG Region** | **World Bank Income Group^3^** |
| --- | --- | --- | --- | --- | --- |
| Afghanistan | Southern Asia | South Asia | EMRO | Southern Asia | Low income |
| Albania | Southern Europe | Europe & Central Asia | EURO | Developed regions | Middle Income |
| Algeria | Northern Africa | Middle East & North Africa | AFRO | Northern Africa | Middle Income |
| American Samoa | Polynesia | Not Classified | Not Classified | Oceania | Middle Income |
| Andorra | Southern Europe | Europe & Central Asia | EURO | Developed regions | High Income |
| Angola | Middle Africa | ESARO | AFRO | Sub-Saharan Africa | Middle Income |
| Anguilla | Caribbean | Not Classified | Not Classified | Latin America & the Caribbean | Not Classified |
| Antigua and Barbuda | Caribbean | Latin America & Caribbean | AMRO | Latin America & the Caribbean | High Income |
| Argentina | South America | Latin America & Caribbean | AMRO | Latin America & the Caribbean | Middle Income |
| Armenia | Western Asia | Europe & Central Asia | EURO | Caucasus and Central Asia | Middle Income |
| Aruba | Caribbean | Not Classified | Not Classified | Latin America & the Caribbean | High Income |
| Australia | Australia and New Zealand | East Asia & Pacific | WPRO | Developed regions | High Income |
| Austria | Western Europe | Europe & Central Asia | EURO | Developed regions | High Income |
| Azerbaijan | Western Asia | Europe & Central Asia | EURO | Caucasus and Central Asia | Middle Income |
| Bahamas | Caribbean | Latin America & Caribbean | AMRO | Latin America & the Caribbean | High Income |
| Bahrain | Western Asia | Middle East & North Africa | EMRO | Western Asia | High Income |
| Bangladesh | Southern Asia | South Asia | SEARO | Southern Asia | Middle Income |
| Barbados | Caribbean | Latin America & Caribbean | AMRO | Latin America & the Caribbean | High Income |
| Belarus | Eastern Europe | Europe & Central Asia | EURO | Developed regions | Middle Income |
| Belgium | Western Europe | Europe & Central Asia | EURO | Developed regions | ${}$   \| High Income \| \| --- \| |
| Belize | Central America | Latin America & Caribbean | AMRO | Latin America & the Caribbean | Middle Income |
| Benin | Western Africa | WCARO | AFRO | Sub-Saharan Africa | Low income |
| Bermuda | Northern America | Not Classified | Not Classified | Developed regions | High Income |
| Bhutan | Southern Asia | South Asia | SEARO | Southern Asia | Middle Income |
| Bolivia (Plurinational State of) | South America | Latin America & Caribbean | AMRO | Latin America & the Caribbean | Middle Income |
| Bosnia and Herzegovina | Southern Europe | Europe & Central Asia | EURO | Developed regions | Middle Income |
| Botswana | Southern Africa | ESARO | AFRO | Sub-Saharan Africa | Middle Income |
| Brazil | South America | Latin America & Caribbean | AMRO | Latin America & the Caribbean | Middle Income |
| British Virgin Islands | Caribbean | Not Classified | Not Classified | Latin America & the Caribbean | High Income |
| Brunei Darussalam | South-eastern Asia | East Asia & Pacific | WPRO | South-eastern Asia | High Income |
| Bulgaria | Eastern Europe | Europe & Central Asia | EURO | Developed regions | Middle Income |
| Burkina Faso | Western Africa | WCARO | AFRO | Sub-Saharan Africa | Low income |
| Burundi | Eastern Africa | ESARO | AFRO | Sub-Saharan Africa | Low income |
| Cabo Verde | Western Africa | WCARO | AFRO | Sub-Saharan Africa | Middle Income |
| Cambodia | South-eastern Asia | East Asia & Pacific | WPRO | South-eastern Asia | Middle Income |
| Cameroon | Middle Africa | WCARO | AFRO | Sub-Saharan Africa | Middle Income |
| Canada | Northern America | North America | AMRO | Developed regions | High Income |
| Cayman Islands | Caribbean | Not Classified | Not Classified | Latin America & the Caribbean | High Income |
| Central African Republic (The) | Middle Africa | WCARO | AFRO | Sub-Saharan Africa | Low income |
| Chad | Middle Africa | WCARO | AFRO | Sub-Saharan Africa | Low income |
| Chile | South America | Latin America & Caribbean | AMRO | Latin America & the Caribbean | High Income |
| China | Eastern Asia | East Asia & Pacific | WPRO | Eastern Asia | Middle Income |
| Colombia | South America | Latin America & Caribbean | AMRO | Latin America & the Caribbean | Middle Income |
| Comoros (The) | Eastern Africa | ESARO | AFRO | Sub-Saharan Africa | Low income |
| Congo (The) | Middle Africa | WCARO | AFRO | Sub-Saharan Africa | Middle Income |
| Cook Islands | Polynesia | East Asia & Pacific | WPRO | Oceania | Not Classified |
| Costa Rica | Central America | Latin America & Caribbean | AMRO | Latin America & the Caribbean | Middle Income |
| Cote d'Ivoire | Western Africa | WCARO | AFRO | Sub-Saharan Africa | Middle Income |
| Croatia | Southern Europe | Europe & Central Asia | EURO | Developed regions | High Income |
| Cuba | Caribbean | Latin America & Caribbean | AMRO | Latin America & the Caribbean | Middle Income |
| Cyprus | Western Asia | Europe & Central Asia | EURO | Developed regions | High Income |
| Czechia | Eastern Europe | Europe & Central Asia | EURO | Developed regions | High Income |
| Democratic People's Rep. of Korea (The) | Eastern Asia | East Asia & Pacific | SEARO | Eastern Asia | Low income |
| Democratic Rep. of the Congo (The) | Middle Africa | WCARO | AFRO | Sub-Saharan Africa | Low income |
| Denmark | Northern Europe | Europe & Central Asia | EURO | Developed regions | High Income |
| Djibouti | Eastern Africa | ESARO | EMRO | Sub-Saharan Africa | Middle Income |
| Dominica | Caribbean | Latin America & Caribbean | AMRO | Latin America & the Caribbean | Middle Income |
| Dominican Republic (The) | Caribbean | Latin America & Caribbean | AMRO | Latin America & the Caribbean | Middle Income |
| Ecuador | South America | Latin America & Caribbean | AMRO | Latin America & the Caribbean | Middle Income |
| Egypt | Northern Africa | Middle East & North Africa | EMRO | Northern Africa | Middle Income |
| El Salvador | Central America | Latin America & Caribbean | AMRO | Latin America & the Caribbean | Middle Income |
| Equatorial Guinea | Middle Africa | WCARO | AFRO | Sub-Saharan Africa | Middle Income |
| Eritrea | Eastern Africa | ESARO | AFRO | Sub-Saharan Africa | Low income |
| Estonia | Northern Europe | Europe & Central Asia | EURO | Developed regions | High Income |
| Ethiopia | Eastern Africa | ESARO | AFRO | Sub-Saharan Africa | Low income |
| Falkland Islands (Malvinas) | South America | Not Classified | Not Classified | Latin America & the Caribbean | Not Classified |
| Fiji | Melanesia | East Asia & Pacific | WPRO | Oceania | Middle Income |
| Finland | Northern Europe | Europe & Central Asia | EURO | Developed regions | High Income |
| France | Western Europe | Europe & Central Asia | EURO | Developed regions | High Income |
| French Guiana | South America | Not Classified | Not Classified | Latin America & the Caribbean | Not Classified |
| French Polynesia | Polynesia | Not Classified | Not Classified | Oceania | High Income |
| Gabon | Middle Africa | WCARO | AFRO | Sub-Saharan Africa | Middle Income |
| Gambia (The) | Western Africa | WCARO | AFRO | Sub-Saharan Africa | Low income |
| Georgia | Western Asia | Europe & Central Asia | EURO | Caucasus and Central Asia | Middle Income |
| Germany | Western Europe | Europe & Central Asia | EURO | Developed regions | High Income |
| Ghana | Western Africa | WCARO | AFRO | Sub-Saharan Africa | Middle Income |
| Gibraltar | Southern Europe | Not Classified | Not Classified | Developed regions | High Income |
| Greece | Southern Europe | Europe & Central Asia | EURO | Developed regions | High Income |
| Greenland | Northern America | Not Classified | Not Classified | Developed regions | High Income |
| Grenada | Caribbean | Latin America & Caribbean | AMRO | Latin America & the Caribbean | Middle Income |
| Guadeloupe | Caribbean | Not Classified | Not Classified | Latin America & the Caribbean | Not Classified |
| Guam | Micronesia | Not Classified | Not Classified | Oceania | High Income |
| Guatemala | Central America | Latin America & Caribbean | AMRO | Latin America & the Caribbean | Middle Income |
| Guinea | Western Africa | WCARO | AFRO | Sub-Saharan Africa | Low income |
| Guinea-Bissau | Western Africa | WCARO | AFRO | Sub-Saharan Africa | Low income |
| Guyana | South America | Latin America & Caribbean | AMRO | Latin America & the Caribbean | Middle Income |
| Haiti | Caribbean | Latin America & Caribbean | AMRO | Latin America & the Caribbean | Low income |
| Honduras | Central America | Latin America & Caribbean | AMRO | Latin America & the Caribbean | Middle Income |
| Hungary | Eastern Europe | Europe & Central Asia | EURO | Developed regions | High Income |
| Iceland | Northern Europe | Europe & Central Asia | EURO | Developed regions | High Income |
| India | Southern Asia | South Asia | SEARO | Southern Asia | Middle Income |
| Indonesia | South-eastern Asia | East Asia & Pacific | SEARO | South-eastern Asia | Middle Income |
| Iran (Islamic Republic of) | Southern Asia | Middle East & North Africa | EMRO | Southern Asia | Middle Income |
| Iraq | Western Asia | Middle East & North Africa | EMRO | Western Asia | Middle Income |
| Ireland | Northern Europe | Europe & Central Asia | EURO | Developed regions | High Income |
| Israel | Western Asia | Middle East & North Africa | EURO | Developed regions | High Income |
| Italy | Southern Europe | Europe & Central Asia | EURO | Developed regions | High Income |
| Jamaica | Caribbean | Latin America & Caribbean | AMRO | Latin America & the Caribbean | Middle Income |
| Japan | Eastern Asia | East Asia & Pacific | WPRO | Developed regions | High Income |
| Jordan | Western Asia | Middle East & North Africa | EMRO | Western Asia | Middle Income |
| Kazakhstan | Central Asia | Europe & Central Asia | EURO | Caucasus and Central Asia | Middle Income |
| Kenya | Eastern Africa | ESARO | AFRO | Sub-Saharan Africa | Middle Income |
| Kiribati | Micronesia | East Asia & Pacific | WPRO | Oceania | Middle Income |
| Kuwait | Western Asia | Middle East & North Africa | EMRO | Western Asia | High Income |
| Kyrgyzstan | Central Asia | Europe & Central Asia | EURO | Caucasus and Central Asia | Middle Income |
| Lao People's Democratic Rep. (The) | South-eastern Asia | East Asia & Pacific | WPRO | South-eastern Asia | Middle Income |
| Latvia | Northern Europe | Europe & Central Asia | EURO | Developed regions | High Income |
| Lebanon | Western Asia | Middle East & North Africa | EMRO | Western Asia | Middle Income |
| Lesotho | Southern Africa | ESARO | AFRO | Sub-Saharan Africa | Middle Income |
| Liberia | Western Africa | WCARO | AFRO | Sub-Saharan Africa | Low income |
| Libya | Northern Africa | Middle East & North Africa | EMRO | Northern Africa | Middle Income |
| Liechtenstein | Western Europe | Europe & Central Asia | Not Classified | Developed regions | High Income |
| Lithuania | Northern Europe | Europe & Central Asia | EURO | Developed regions | High Income |
| Luxembourg | Western Europe | Europe & Central Asia | EURO | Developed regions | High Income |
| Madagascar | Eastern Africa | ESARO | AFRO | Sub-Saharan Africa | Low income |
| Malawi | Eastern Africa | ESARO | AFRO | Sub-Saharan Africa | Low income |
| Malaysia | South-eastern Asia | East Asia & Pacific | WPRO | South-eastern Asia | Middle Income |
| Maldives | Southern Asia | South Asia | SEARO | Southern Asia | Middle Income |
| Mali | Western Africa | WCARO | AFRO | Sub-Saharan Africa | Low income |
| Malta | Southern Europe | Europe & Central Asia | EURO | Developed regions | High Income |
| Marshall Islands | Micronesia | East Asia & Pacific | WPRO | Oceania | Middle Income |
| Martinique | Caribbean | Not Classified | Not Classified | Latin America & the Caribbean | Not Classified |
| Mauritania | Western Africa | WCARO | AFRO | Sub-Saharan Africa | Middle Income |
| Mauritius | Eastern Africa | ESARO | AFRO | Sub-Saharan Africa | Middle Income |
| Mexico | Central America | Latin America & Caribbean | AMRO | Latin America & the Caribbean | Middle Income |
| Micronesia (Federated States of) | Micronesia | East Asia & Pacific | WPRO | Oceania | Middle Income |
| Monaco | Western Europe | Europe & Central Asia | EURO | Developed regions | High Income |
| Mongolia | Eastern Asia | East Asia & Pacific | WPRO | Eastern Asia | Middle Income |
| Montenegro | Southern Europe | Europe & Central Asia | EURO | Developed regions | Middle Income |
| Montserrat | Caribbean | Not Classified | Not Classified | Latin America & the Caribbean | Not Classified |
| Morocco | Northern Africa | Middle East & North Africa | EMRO | Northern Africa | Middle Income |
| Mozambique | Eastern Africa | ESARO | AFRO | Sub-Saharan Africa | Low income |
| Myanmar | South-eastern Asia | East Asia & Pacific | SEARO | South-eastern Asia | Middle Income |
| Namibia | Southern Africa | ESARO | AFRO | Sub-Saharan Africa | Middle Income |
| Nauru | Micronesia | East Asia & Pacific | WPRO | Oceania | High Income |
| Nepal | Southern Asia | South Asia | SEARO | Southern Asia | Low income |
| Netherlands | Western Europe | Europe & Central Asia | EURO | Developed regions | High Income |
| Netherlands Antilles | Caribbean | Not Classified | Not Classified | Latin America & the Caribbean | Not Classified |
| New Caledonia | Melanesia | Not Classified | Not Classified | Oceania | Not Classified |
| New Zealand | Australia and New Zealand | East Asia & Pacific | WPRO | Developed regions | High Income |
| Nicaragua | Central America | Latin America & Caribbean | AMRO | Latin America & the Caribbean | Middle Income |
| Niger (The) | Western Africa | WCARO | AFRO | Sub-Saharan Africa | Low income |
| Nigeria | Western Africa | WCARO | AFRO | Sub-Saharan Africa | Middle Income |
| Niue | Polynesia | East Asia & Pacific | WPRO | Oceania | Not Classified |
| Norway | Northern Europe | Europe & Central Asia | EURO | Developed regions | High Income |
| Oman | Western Asia | Middle East & North Africa | EMRO | Western Asia | High Income |
| Pakistan | Southern Asia | South Asia | EMRO | Southern Asia | Middle Income |
| Palau | Micronesia | East Asia & Pacific | WPRO | Oceania | Middle Income |
| Panama | Central America | Latin America & Caribbean | AMRO | Latin America & the Caribbean | Middle Income |
| Papua New Guinea | Melanesia | East Asia & Pacific | WPRO | Oceania | Middle Income |
| Paraguay | South America | Latin America & Caribbean | AMRO | Latin America & the Caribbean | Middle Income |
| Peru | South America | Latin America & Caribbean | AMRO | Latin America & the Caribbean | Middle Income |
| Philippines (The) | South-eastern Asia | East Asia & Pacific | WPRO | South-eastern Asia | Middle Income |
| Poland | Eastern Europe | Europe & Central Asia | EURO | Developed regions | High Income |
| Portugal | Southern Europe | Europe & Central Asia | EURO | Developed regions | High Income |
| Puerto Rico | Caribbean | Not Classified | Not Classified | Latin America & the Caribbean | Not Classified |
| Qatar | Western Asia | Middle East & North Africa | EMRO | Western Asia | High Income |
| Republic of Korea (The) | Eastern Asia | East Asia & Pacific | WPRO | Eastern Asia | High Income |
| Republic of Moldova (The) | Eastern Europe | Europe & Central Asia | EURO | Developed regions | Middle Income |
| Reunion | Eastern Africa | Not Classified | Not Classified | sub-Saharan Africa | Not Classified |
| Romania | Eastern Europe | Europe & Central Asia | EURO | Developed regions | Middle Income |
| Russian Federation (The) | Eastern Europe | Europe & Central Asia | EURO | Developed regions | Middle Income |
| Rwanda | Eastern Africa | ESARO | AFRO | Sub-Saharan Africa | Low income |
| Saint Kitts and Nevis | Caribbean | Latin America & Caribbean | AMRO | Latin America & the Caribbean | High Income |
| Saint Lucia | Caribbean | Latin America & Caribbean | AMRO | Latin America & the Caribbean | Middle Income |
| Saint Vincent and the Grenadines | Caribbean | Latin America & Caribbean | AMRO | Latin America & the Caribbean | Middle Income |
| Samoa | Polynesia | East Asia & Pacific | WPRO | Oceania | Middle Income |
| San Marino | Southern Europe | Europe & Central Asia | EURO | Developed regions | High Income |
| Sao Tome and Principe | Middle Africa | WCARO | AFRO | Sub-Saharan Africa | Middle Income |
| Saudi Arabia | Western Asia | Middle East & North Africa | EMRO | Western Asia | High Income |
| Senegal | Western Africa | WCARO | AFRO | Sub-Saharan Africa | Low income |
| Serbia | Southern Europe | Europe & Central Asia | EURO | Developed regions | Middle Income |
| Seychelles | Eastern Africa | ESARO | AFRO | Sub-Saharan Africa | High Income |
| Sierra Leone | Western Africa | WCARO | AFRO | Sub-Saharan Africa | Low income |
| Singapore | South-eastern Asia | East Asia & Pacific | WPRO | South-eastern Asia | High Income |
| Slovakia | Eastern Europe | Europe & Central Asia | EURO | Developed regions | High Income |
| Slovenia | Southern Europe | Europe & Central Asia | EURO | Developed regions | High Income |
| Solomon Islands | Melanesia | East Asia & Pacific | WPRO | Oceania | Middle Income |
| Somalia | Eastern Africa | ESARO | EMRO | Sub-Saharan Africa | Low income |
| South Africa | Southern Africa | ESARO | AFRO | Sub-Saharan Africa | Middle Income |
| South Sudan | Eastern Africa | ESARO | AFRO | Sub-Saharan Africa | Low income |
| Spain | Southern Europe | Europe & Central Asia | EURO | Developed regions | High Income |
| Sri Lanka | Southern Asia | South Asia | SEARO | Southern Asia | Middle Income |
| Sudan (The) | Northern Africa | ESARO | EMRO | Sub-Saharan Africa | Middle Income |
| Suriname | South America | Latin America & Caribbean | AMRO | Latin America & the Caribbean | Middle Income |
| Swaziland | Southern Africa | ESARO | AFRO | Sub-Saharan Africa | Middle Income |
| Sweden | Northern Europe | Europe & Central Asia | EURO | Developed regions | High Income |
| Switzerland | Western Europe | Europe & Central Asia | EURO | Developed regions | High Income |
| Syrian Arab Republic (The) | Western Asia | Middle East & North Africa | EMRO | Western Asia | Middle Income |
| Tajikistan | Central Asia | Europe & Central Asia | EURO | Caucasus and Central Asia | Middle Income |
| Thailand | South-eastern Asia | East Asia & Pacific | SEARO | South-eastern Asia | Middle Income |
| The Former Yugoslav Republic of Macedonia | Southern Europe | Europe & Central Asia | EURO | Developed regions | Middle Income |
| Timor-Leste | South-eastern Asia | East Asia & Pacific | SEARO | South-eastern Asia | Middle Income |
| Togo | Western Africa | WCARO | AFRO | Sub-Saharan Africa | Low income |
| Tokelau | Polynesia | Not Classified | Not Classified | Oceania | Not Classified |
| Tonga | Polynesia | East Asia & Pacific | WPRO | Oceania | Middle Income |
| Trinidad and Tobago | Caribbean | Latin America & Caribbean | AMRO | Latin America & the Caribbean | High Income |
| Tunisia | Northern Africa | Middle East & North Africa | EMRO | Northern Africa | Middle Income |
| Turkey | Western Asia | Europe & Central Asia | EURO | Western Asia | Middle Income |
| Turkmenistan | Central Asia | Europe & Central Asia | EURO | Caucasus and Central Asia | Middle Income |
| Turks and Caicos Islands | Caribbean | Not Classified | Not Classified | Latin America & the Caribbean | Not Classified |
| Tuvalu | Polynesia | East Asia & Pacific | WPRO | Oceania | Middle Income |
| Uganda | Eastern Africa | ESARO | AFRO | Sub-Saharan Africa | Low income |
| Ukraine | Eastern Europe | Europe & Central Asia | EURO | Developed regions | Middle Income |
| United Arab Emirates | Western Asia | Middle East & North Africa | EMRO | Western Asia | High Income |
| United Kingdom (The) | Northern Europe | Europe & Central Asia | EURO | Developed regions | High Income |
| United Republic of Tanzania (The) | Eastern Africa | ESARO | AFRO | Sub-Saharan Africa | Low income |
| United States of America (The) | Northern America | North America | AMRO | Developed regions | High Income |
| Uruguay | South America | Latin America & Caribbean | AMRO | Latin America & the Caribbean | High Income |
| Uzbekistan | Central Asia | Europe & Central Asia | EURO | Caucasus and Central Asia | Middle Income |
| Vanuatu | Melanesia | East Asia & Pacific | WPRO | Oceania | Middle Income |
| Venezuela (Bolivarian Republic of) | South America | Latin America & Caribbean | AMRO | Latin America & the Caribbean | Middle Income |
| Viet Nam | South-eastern Asia | East Asia & Pacific | WPRO | South-eastern Asia | Middle Income |
| Virgin Islands (USA) | Caribbean | Not Classified | Not Classified | Latin America & the Caribbean | Not Classified |
| Wallis and Futuna | Polynesia | Not Classified | Not Classified | Not Classified | Not Classified |
| West Bank and Gaza | Western Asia | Middle East & North Africa | Not Classified | Western Asia | Middle Income |
| Yemen | Western Asia | Middle East & North Africa | EMRO | Western Asia | Middle Income |
| Zambia | Eastern Africa | ESARO | AFRO | Sub-Saharan Africa | Middle Income |
| Zimbabwe | Eastern Africa | ESARO | AFRO | Sub-Saharan Africa | Low income |

^1^UNICEF regional abbreviations and full names: Central and Eastern Europe/Commonwealth of Independent States (CEE- CIS), Developed regions (Dev), East Asia and Pacific (EAP), Eastern and Southern Africa (ESA), Middle East and North Africa (MENA), South Asia (SA), West and Central Africa (WCA), The Americas and Caribbean (TAC).

^2^ WHO regional abbreviations and full names: Africa (AFR), Americas (AMR), Eastern Mediterranean (EMR), Europe (EUR), South-East Asia (SEAR), Western Pacific (WPR).

^3^ Based on FY17 World Bank income classification^4^ 3 Based on FY17 World Bank income classification

| **Supplementary Table 5. Characteristics of studies on prevalence** | | | | | | | | | | | | |
| --- | --- | --- | --- | --- | --- | --- | --- | --- | --- | --- | --- | --- |
|  | | | | | | | | | | | | |
| **Ref*** | **Author** | | **Year** | **Region** | **Period** | **Place** | **Criteria** | **WC** | **Age** | **Case** | **Sample** | **Prevalence** |
| 1 | Ford | | 2002 | USA | 1985-1999 | mixed | ATP-III | TS | ≥20 | 3402 | 8814 | 38.60% |
| 2 | Al-Lawati | | 2003 | Oman | 2000-2004 | urban | ATP-III | TS | ≥20 | 349 | 1419 | 24.60% |
| 3 | Azizi | | 2003 | Iran | 2000-2004 | urban | ATP-III | TS | ≥20 | 2954 | 9846 | 30.00% |
| 4 | Ford | | 2003 | USA | 1985-1999 | mixed | mixed | TS | ≥20 | 4571 | 8608 | 53.10% |
| 5 | Park | | 2003 | USA | 1985-1999 | mixed | ATP-III | TS | 30-79 | 5662 | 13157 | 43.03% |
| 6 | Tanchoco | | 2003 | Philippines | 1985-1999 | mixed | WHO | NM | ≥20 | 696 | 4539 | 15.33% |
| 7 | Chuang | | 2004 | Taiwan | 2000-2004 | mixed | NM | TS | NM | 2153 | 24329 | 8.85% |
| 8 | Farrell | | 2004 | USA | 1985-1999 | NM | ATP-III | TS | 20-80 | 618 | 7104 | 8.70% |
| 9 | Ford | | 2004 | USA | 1985-2004 | mixed | ATP-III | TS | ≥20 | 3202 | 8113 | 39.48% |
| 10 | Gupta | | 2004 | India | NM | urban | ATP-III | TS | >20 | 362 | 1091 | 33.18% |
| 11 | Jia | | 2004 | China | 2000-2004 | urban | mixed | TS | 20-74 | 463 | 2048 | 22.63% |
| 12 | Jorgensen | | 2004 | Greenland | NM | mixed | ATP-III | TS | >35 | 339 | 917 | 36.92% |
| 13 | Kim | | 2004 | South Korea | 1985-1999 | mixed | mixed | TS | ≥25 | 2305 | 6147 | 37.50% |
| 14 | Oh | | 2004 | South Korea | 1985-1999 | urban | mixed | TS | 30-80 | 127 | 774 | 16.38% |
| 15 | Parikka | | 2004 | Finland | 1985-1999 | NM | mixed | TS | 45–64 | 1594 | 3210 | 49.65% |
| 16 | Santos | | 2004 | Portugal | NM | urban | ATP-III | TS | ≥18 | 438 | 1436 | 30.50% |
| 17 | Al-Nozha | | 2005 | Saudi Arabia | 1985-1999 | mixed | ATP-III | TS | 30-70 | 6943 | 16941 | 40.98% |
| 18 | Athyros | | 2005 | Greece | 2000-2004 | mixed | ATP-III | TS | >18 | 2359 | 4153 | 56.80% |
| 19 | Bo | | 2005 | Italy | NM | mixed | ATP-III | TS | 45-64 | 608 | 1657 | 36.70% |
| 20 | Boronat | | 2005 | Spain | NM | NM | mixed | TS | >30 | 704 | 987 | 71.35% |
| 21 | Choi | | 2005 | South Korea | 2000-2004 | NM | mixed | TS | 30-79 | 688 | 1230 | 55.91% |
| 22 | Dekker | | 2005 | Netherlands | 1985-1999 | NM | mixed | TS | 50-75 | 678 | 1400 | 48.42% |
| 23 | Florez | | 2005 | Venezuela | 2000-2004 | NM | ATP-III | TS | ≥20 | 1333 | 3108 | 42.90% |
| 24 | Grandinetti | | 2005 | USA | 1985-1999 | rural | ATP-III | TS | ≥18 | 568 | 1503 | 37.80% |
| 25 | Ko | | 2005 | China | 1985-1999 | NM | mixed | TS | 18-66 | 286 | 1513 | 18.91% |
| 26 | Li | | 2005 | China | 2000-2004 | urban | ATP-III | TS | ≥60 | 918 | 1677 | 54.74% |
| 27 | Miccoli | | 2005 | Italy | 1985-1999 | NM | ATP-III | TS | ≥19 | 800 | 2100 | 38.10% |
| 28 | Romero | | 2005 | Mexico | NM | NM | mixed | NM | 30-64 | 187 | 700 | 26.67% |
| 29 | Rosenbaum | | 2005 | Brazil | NM | NM | mixed | TS | >30 | 463 | 1166 | 39.68% |
| 30 | Son le | | 2005 | Vietnam | 2000-2004 | urban | ATP-III | TS | ≥20 | 185 | 611 | 30.20% |
| 31 | Tanaka | | 2005 | Japan | 2000-2004 | NM | ATP-III | TS | 30-79 | 2742 | 6985 | 39.25% |
| 32 | Thomas | | 2005 | China | 1985-1999 | NM | mixed | TS | NM | 462 | 2843 | 16.25% |
| 33 | Tillin | | 2005 | UK | 1985-1999 | NM | mixed | TS | 40-69 | 2170 | 4705 | 46.12% |
| 34 | Urashima | | 2005 | Japan | 2000-2004 | NM | mixed | TS | ≥20 | 4888 | 22892 | 21.35% |
| 35 | Yan | | 2005 | China | NM | rural | IDF | TS | NM | 769 | 2148 | 35.79% |
| 36 | Zhang | | 2005 | China | NM | NM | mixed | TS | 30-84 | 565 | 815 | 69.30% |
| 37 | Arai | | 2006 | Japan | 2000-2004 | NM | J-MS | TS | 20-79 | 1054 | 3264 | 32.30% |
| 38 | Bouguerra | | 2006 | Tunisia | 1985-1999 | mixed | ATP-III | TS | ≥20 | 852 | 3433 | 24.81% |
| 39 | Fakhrzadeh | | 2006 | Iran | NM | urban | ATP-III | TS | 25-64 | 620 | 1480 | 41.90% |
| 40 | Feng | | 2006 | China | 2005-2009 | rural | mixed | TS | 25-64 | 3225 | 18595 | 17.34% |
| 41 | Harzallah | | 2006 | Tunisia | 1985-1999 | urban | mixed | TS | ≥40 | 544 | 863 | 63.00% |
| 42 | He | | 2006 | China | 2000-2004 | urban | mixed | TS | 60-95 | 1131 | 2334 | 48.45% |
| 43 | Hu | | 2006 | China | NM | mixed | IDF | TS | 35-74 | 1937 | 5888 | 32.90% |
| 44 | Ko | | 2006 | China | 2000-2004 | NM | IDF | TS | 18-66 | 262 | 1513 | 17.30% |
| 45 | Lawati | | 2006 | Oman | 2000-2004 | NM | IDF | NM | ≥20 | 551 | 1419 | 38.83% |
| 46 | Liu | | 2006 | China | 1985-1999 | NM | mixed | TS | ≥18 | 8644 | 30448 | 28.39% |
| 47 | Liu | | 2006 | China | 2000-2004 | mixed | mixed | TS | 15-74 | 2150 | 14327 | 15.01% |
| 48 | Lu | | 2006 | Canada | 1980-1990 | mixed | mixed | TS | ≥18 | 1267 | 3476 | 36.5 % |
| 49 | Miyatake | | 2006 | Japan | NM | NM | mixed | TS | 20-79 | 2222 | 12712 | 17.48% |
| 50 | Nilsson | | 2006 | Sweden | 2000-2004 | NM | ATP-III | TS | 70 | 179 | 508 | 35.30% |
| 51 | PARK | | 2006 | South Korea | 1985-1999 | NM | IDF | TS | 20-80 | 1441 | 6824 | 21.11% |
| 52 | Patel | | 2006 | USA;Taiwan | 1985-2004 | NM | ATP-III | TS | ≥35 | 23899 | 65478 | 36.50% |
| 53 | Qahtani | | 2006 | Saudi Arabia | 2005-2009 | mixed | mixed | TS | 18–59 | 1100 | 1922 | 57.21% |
| 54 | Ravaglia | | 2006 | Italy | 2000-2004 | NM | NHLBI | TS | ≥65 | 475 | 981 | 48.42% |
| 55 | Seclen | | 2006 | Peru | 2000-2004 | urban | ATP-III | TS | 30-92 | 523 | 612 | 85.40% |
| 56 | Szurkowska | | 2006 | Poland | 2005-2009 | NM | ATP-III | TS | 25-29 | 11848 | 40989 | 28.90% |
| 57 | DECODA | | 2007 | China; Japan; India | 1985-1999 | NM | mixed | TS | 25-74 | 2773 | 14221 | 19.50% |
| 58 | Deepa | | 2007 | India | 2000-2004 | urban | mixed | TS | ≥20 | 978 | 2350 | 41.62% |
| 59 | Hildrum | | 2007 | Norway | 1985-1999 | NM | mixed | TS | 20-89 | 3473 | 10206 | 34.03% |
| 60 | Hwang | | 2007 | Taiwan | 2000-2004 | NM | NHLBI | TS | 20-79 | 1764 | 5880 | 30.00% |
| 61 | Khader | | 2007 | Jordan | 2000-2004 | NM | ATP-III | TS | ≥25 | 567 | 1121 | 50.60% |
| 62 | Kim | | 2007 | South Korea | 2000-2004 | NM | mixed | TS | ≥20 | 1314 | 4452 | 29.51% |
| 63 | Kozan | | 2007 | Turkey | NM | mixed | ATP-III | TS | 20-90 | 1542 | 4259 | 36.20% |
| 64 | Li | | 2007 | China | 1985-1999 | mixed | mixed | TS | 35-64 | 31411 | 60756 | 51.70% |
| 65 | Lin | | 2007 | Taiwan | 2005-2009 | urban | ATP-III | TS | ≥40 | 642 | 2359 | 27.21% |
| 66 | Mattsson | | 2007 | Finland | 1985-1999 | mixed | mixed | TS | 24-39 | 547 | 2182 | 25.07% |
| 67 | Park | | 2007 | South Korea | 1995-2004 | NM | ATP-III | TS | ≥20 | 1208 | 13409 | 9.01% |
| 68 | Prabhakaran | | 2007 | India | 1985-1999 | mixed | mixed | TS | 35-64 | 538 | 4044 | 13.30% |
| 69 | Sandhofer | | 2007 | Austria | NM | NM | mixed | TS | 40-70 | 776 | 1518 | 51.11% |
| 70 | Santos | | 2007 | Portugal | NM | NM | mixed | TS | 18-92 | 824 | 1433 | 57.51% |
| 71 | Yang | | 2007 | China | 2000-2004 | mixed | mixed | TS | 35-74 | 4773 | 15167 | 31.47% |
| 72 | Yoon | | 2007 | South Korea | 1985-1999 | NM | mixed | TS | ≥20 | 2571 | 7962 | 32.30% |
| 73 | Zabetian | | 2007 | Iran | 2000-2004 | NM | mixed | TS | ≥20 | 4396 | 10368 | 42.40% |
| 74 | Bindraban | | 2008 | Netherlands | 2000-2004 | mixed | mixed | TS | 35-60 | 774 | 1402 | 55.17% |
| 75 | Erem | | 2008 | Turkey | 2000-2004 | NM | ATP-III | TS | >20 | 1965 | 4809 | 40.86% |
| 76 | Fiuza | | 2008 | Portugal | 2005-2009 | urban | ATP-III | TS | 18-96 | 7818 | 16856 | 46.38% |
| 77 | Hu | | 2008 | Finland | 1985-2004 | NM | mixed | TS | 45-64 | 1769 | 3495 | 50.62% |
| 78 | Kelliny | | 2008 | Seychelles | 2000-2004 | NM | mixed | TS | 25-64 | 645 | 1218 | 52.92% |
| 79 | Maggi | | 2008 | Italy | 1985-1994 | NM | ATP-III | TS | 65-84 | 2509 | 4498 | 55.77% |
| 80 | Malik | | 2008 | United Arab Emirates | 2000-2004 | mixed | mixed | TS | ≥20 | 2369 | 4097 | 57.81% |
| 81 | Marquezine | | 2008 | Brazil | NM | urban | ATP-III | NM | 25-64 | 255 | 1561 | 16.34% |
| 82 | Moebus | | 2008 | Germany | 2005-2009 | NM | NHLBI | TS | 18-99 | 14170 | 35869 | 39.50% |
| 83 | Mokan | | 2008 | Slovakia | NM | NM | mixed | TS | ≥18 | 608 | 1517 | 40.10% |
| 84 | Neuhauser | | 2008 | Germany | 1985-1999 | NM | ATP-III | TS | 18-79 | 2173 | 6666 | 32.60% |
| 85 | Park | | 2008 | South Korea | 1985-1999 | NM | mixed | TS | ≥20 | 7833 | 38551 | 20.32% |
| 86 | Park | | 2008 | USA | 2000-2004 | urban | mixed | TS | 20-39 | 422 | 2273 | 18.55% |
| 87 | Perez | | 2008 | USA | 2005-2009 | urban | ATP-III | TS | 21-79 | 421 | 859 | 49.00% |
| 88 | Ramos | | 2008 | USA | 1985-2004 | NM | ATPIII | TS | 18-44 | 2046 | 5418 | 37.76% |
| 89 | Suarez | | 2008 | Spain | 2005-2009 | NM | NHLBI | TS | 50-70 | 662 | 858 | 77.20% |
| 90 | Sun | | 2008 | Taiwan | 2005-2009 | urban | mixed | TS | 35-74 | 7377 | 28408 | 25.97% |
| 91 | Surana | | 2008 | India | NM | urban | ATP-III | TS | NM | 2344 | 5088 | 46.07% |
| 92 | Welin | | 2008 | Sweden | 2000-2004 | urban | mixed | TS | 50-60 | 933 | 1917 | 48.65% |
| 93 | Arikan | | 2009 | Turkey | 2005-2009 | mixed | ATP-III | TS | 20-69 | 1063 | 2766 | 38.43% |
| 94 | Bener | | 2009 | Qatar | 2005-2009 | mixed | mixed | TS | >20 | 896 | 1204 | 74.38% |
| 95 | Buckland | | 2009 | Spain | 1985-2004 | NM | mixed | TS | 18-75 | 733 | 1959 | 37.42% |
| 96 | Can | | 2009 | Turkey | 1995-2004 | mixed | TCRP | TS | 32-58 | 1016 | 1690 | 60.10% |
| 97 | Delavar | | 2009 | Iran | NM | urban | ATP-III | TS | 30-50 | 723 | 944 | 76.59% |
| 98 | Donk | | 2009 | Netherlands | 2005-2009 | NM | ATP-III | TS | 20-70 | 4033 | 11862 | 34.00% |
| 99 | Ekelund | | 2009 | Denmark, Estonia, Portugal | 1985-1999 | mixed | IDF | TS | 10&15 | 281 | 1597 | 17.57% |
| 100 | Elasmi | | 2009 | Great Tunis | 2005-2009 | NM | ATP-III | NM | 30-70 | 1244 | 2483 | 50.09% |
| 101 | Ervin | | 2009 | USA | 2005-2009 | NM | ATP-III | TS | ≥20 | 3526 | 6600 | 53.43% |
| 102 | Fiuza | | 2009 | Portugal | NM | mixed | ATP-III | TS | >18 | 7636 | 16457 | 46.40% |
| 103 | Gelaye | | 2009 | Peru | NM | urban | ATP-III | TS | >18 | 668 | 1675 | 39.89% |
| 104 | Gundogan | | 2009 | Turkey | 2000-2004 | mixed | ATP-III | TS | 20-83 | 436 | 787 | 55.40% |
| 105 | Hadaegh | | 2009 | Iran | 2000-2004 | NM | mixed | TS | ≥65 | 441 | 720 | 61.25% |
| 106 | Quintana | | 2009 | Peru | NM | mixed | IDF | TS | ≥20 | 2659 | 4053 | 65.60% |
| 107 | Saucedo | | 2009 | Mexico | NM | urban | mixed | TS | 18-60 | 195 | 608 | 32.05% |
| 108 | Sharifi | | 2009 | Iran | 2000-2004 | urban | mixed | TS | >20 | 772 | 2941 | 26.24% |
| 109 | Tao | | 2009 | China | 2005-2009 | mixed | IDF | TS | >20 | 708 | 4116 | 17.20% |
| 110 | Xu | | 2009 | China | 2000-2004 | rural | IDF | TS | ≥20 | 911 | 2536 | 35.92% |
| 111 | Zuo | | 2009 | China | 2000-2004 | mixed | ATP-III | TS | 35-74 | 1299 | 3914 | 33.20% |
| 112 | Al-Daghri | | 2010 | Saudi Arabia | NM | urban | ATP-III | TS | 18-55 | 1114 | 2850 | 39.10% |
| 113 | Allal-Elasmi | | 2010 | Tunisia | 2005-2009 | NM | ATP-III | TS | 35-70 | 1287 | 2712 | 47.46% |
| 114 | Bhat | | 2010 | India | NM | Rural | ATP-III | TS | 20-60 | 26 | 500 | 5.20% |
| 115 | Biad | | 2010 | Algeria | 2005-2009 | urban | mixed | TS | ≥20 | 1163 | 1211 | 96.00% |
| 116 | Ferguson | | 2010 | Jamaica | 2005-2009 | mixed | IDF | TS | 18-20 | 134 | 839 | 16.00% |
| 117 | Ferguson | | 2010 | Jamaica | 1985-1999 | NM | mixed | TS | 25-74 | 672 | 1870 | 35.95% |
| 118 | Flowers | | 2010 | USA | 2005-2009 | NM | IDF | TS | 21-82 | 858 | 1445 | 59.38% |
| 119 | Guo | | 2010 | China | 2005-2009 | NM | mixed | TS | ≥18 | 1094 | 2745 | 39.85% |
| 120 | Khunti | | 2010 | UK | NM | NM | mixed | TS | 40-75 | 1858 | 3099 | 59.96% |
| 121 | Li | | 2010 | China | 2005-2009 | mixed | mixed | NM | ≥18 | 5023 | 16442 | 30.55% |
| 122 | Li | | 2010 | China | 2005-2009 | NM | ATP-III | TS | 17-92 | 446 | 1206 | 36.98% |
| 123 | Misra | | 2010 | USA | NM | urban | mixed | TS | 19-91 | 434 | 1038 | 41.85% |
| 124 | Moebus | | 2010 | Germany | 2005-2009 | NM | mixed | TS | 18-99 | 14175 | 35869 | 39.52% |
| 125 | Oguz | | 2010 | Turkey | 2000-2004 | mixed | mixed | NM | 35-70 | 1312 | 2312 | 56.77% |
| 126 | Oladapo | | 2010 | Nigeria | 2000-2004 | rural | ATP-III | TS | 18-64 | 124 | 2000 | 6.20% |
| 127 | Ravikiran | | 2010 | India | NM | NM | mixed | TS | ≥20 | 1022 | 2225 | 45.95% |
| 128 | Schipf | | 2010 | Germany | 1985-1999 | NM | IDF | TS | ≥20 | 2086 | 4223 | 49.40% |
| 129 | Sidorenkov | | 2010 | Russia | 2000-2004 | urban | mixed | TS | 18-90 | 698 | 3555 | 19.64% |
| 130 | Valenzuela | | 2010 | Chile | 2000-2004 | mixed | mixed | TS | ≥17 | 817 | 1833 | 44.55% |
| 131 | Wang | | 2010 | China | NM | mixed | ATP-III | TS | NM | 1365 | 3785 | 36.06% |
| 132 | Xavier | | 2010 | Brazil | 2005-2009 | urban | ATP-III | TS | 30-88 | 415 | 650 | 63.85% |
| 133 | Xu | | 2010 | China | 2005-2009 | urban | mixed | TS | 20-79 | 1895 | 5494 | 34.49% |
| 134 | Zhao | | 2010 | China | 2005-2009 | NM | IDF | TS | 35-74 | 303 | 553 | 54.79% |
| 135 | Zhao | | 2010 | China | NM | rural | IDF | TS | 25-74 | 544 | 1612 | 33.75% |
| 136 | Aekplakorn | | 2011 | Thailand | 2005-2009 | mixed | Harmonized | TS | ≥20 | 6295 | 19256 | 32.69% |
| 137 | Alkerwi | | 2011 | Luxembourg | 2005-2009 | NM | mixed | TS | 18-69 | 451 | 1349 | 33.45% |
| 138 | Alvi | | 2011 | Pakistan | 2000-2004 | urban | IDF | TS | >25 | 529 | 856 | 61.76% |
| 139 | Burazor | | 2011 | Serbia | NM | NM | ATP-III | TS | ≥30 | 935 | 1051 | 89.00% |
| 140 | Gavrila | | 2011 | Spain | 2000-2004 | mixed | mixed | TS | >20 | 822 | 1555 | 52.85% |
| 141 | Janszky | | 2011 | Poland | 2010-2014 | mixed | IDF | TS | 45-64 | 2897 | 3862 | 75.01% |
| 142 | Jiang | | 2011 | China | NM | mixed | IDF | TS | 30-80 | 1334 | 2053 | 64.98% |
| 143 | Kawada | | 2011 | Japan | 2010-2014 | NM | ATP-III | TS | 30-69 | 2952 | 3149 | 93.73% |
| 144 | Li | | 2011 | China | NM | NM | mixed | TS | NM | 871 | 8913 | 9.78% |
| 145 | Lim | | 2011 | South Korea | 1985-2004 | NM | ATP-III | TS | >20 | 12832 | 33091 | 38.78% |
| 146 | Mohamud | | 2011 | Malaysia | 2005-2009 | mixed | mixed | TS | >18 | 2492 | 4341 | 57.41% |
| 147 | Motala | | 2011 | South Africa | NM | rural | mixed | TS | >15 | 443 | 947 | 46.83% |
| 148 | Oliveira | | 2011 | Brazil | 2005-2009 | rural | IDF | TS | 18-69 | 369 | 606 | 60.89% |
| 149 | Orhan | | 2011 | Turkey | 2005-2009 | mixed | mixed | TS | 20-85 | 708 | 807 | 87.73% |
| 150 | Pan | | 2011 | USA | 2005-2009 | NM | ATP-III | NM | ≥18 | 1258 | 1847 | 68.11% |
| 151 | Pimenta | | 2011 | Brazil | 2005-2009 | rural | ATP-III | TS | ≥18 | 62 | 534 | 11.61% |
| 152 | Riediger | | 2011 | Canada | 2005-2009 | NM | ATP-III | TS | ≥18 | 630 | 1800 | 35.00% |
| 153 | Sawant | | 2011 | India | NM | urban | NHLBI | NM | ≥20 | 389 | 548 | 70.90% |
| 154 | Sharma | | 2011 | Nepal | NM | NM | mixed | TS | 20-100 | 3307 | 14425 | 22.92% |
| 155 | Tsai | | 2011 | Taiwan | 2005-2009 | NM | ATP-III | TS | 48 | 52 | 579 | 8.98% |
| 156 | Wei | | 2011 | China | 2005-2009 | mixed | IDF | TS | >35 | 2768 | 4019 | 68.87% |
| 157 | Wen | | 2011 | Taiwan | 2000-2004 | NM | mixed | TS | NM | 47540 | 486341 | 9.78% |
| 158 | Zhao | | 2011 | China | 2005-2009 | mixed | IDF | TS | 35-74 | 2860 | 5355 | 53.40% |
| 159 | Al Zenki | | 2012 | Kuwait | 2005-2009 | NM | mixed | TS | 20-86 | 640 | 992 | 64.50% |
| 160 | Alzahrani | | 2012 | Saudi Arabia | 2010-2014 | NM | ATP-III | TS | 20-50 | 129 | 600 | 21.48% |
| 161 | Cai | | 2012 | China | 2005-2009 | rural | mixed | TS | 18-74 | 5139 | 13505 | 38.05% |
| 162 | Cetin | | 2012 | Turkey | 2005-2009 | NM | ATP-III | TS | ≥20 | 305 | 669 | 45.59% |
| 163 | Deka | | 2012 | Croatia | 2005-2009 | mixed | mixed | TS | 18-97 | 790 | 1397 | 56.55% |
| 164 | Fei | | 2012 | China | 2005-2009 | mixed | mixed | TS | ≥20 | 1251 | 3511 | 35.64% |
| 165 | Ilow | | 2012 | Poland | 2000-2004 | NM | Harmonized | TS | 40-50 | 9334 | 18583 | 50.23% |
| 166 | Jesmin | | 2012 | Bangladesh | NM | rural | mixed | TS | 15-85 | 327 | 1535 | 21.31% |
| 167 | Kaduka | | 2012 | Kenya | 2005-2009 | urban | IDF | TS | ≥18 | 270 | 539 | 50.01% |
| 168 | Kim | | 2012 | South Korea | 2005-2009 | mixed | NHLBI | TS | >30 | 1123 | 2026 | 55.41% |
| 169 | Lao | | 2012 | China | 2000-2004 | mixed | IDF | TS | ≥20 | 951 | 5842 | 16.28% |
| 170 | Li | | 2012 | China | 2010-2014 | NM | IDF | TS | ≥18 | 1531 | 3442 | 44.48% |
| 171 | Mahjoub | | 2012 | Iran | 2005-2009 | NM | mixed | TS | >20 | 425 | 856 | 49.65% |
| 172 | Metelskaya | | 2012 | Russia | 2005-2009 | NM | ATP-III | TS | 55-92 | 1535 | 3576 | 42.92% |
| 173 | Mora Garcia | | 2012 | Colombia | 2005-2009 | urban | mixed | TS | 20-80 | 816 | 879 | 92.78% |
| 174 | Nezhad | | 2012 | France | 2000-2004 | NM | ATP-III | TS | 36-55 | 669 | 2579 | 25.95% |
| 175 | Prasad | | 2012 | India | NM | urban | Harmonized | TS | ≥20 | 576 | 1178 | 48.90% |
| 176 | Rampal | | 2012 | Malaysia | 2000-2004 | NM | Harmonized | TS | ≥15 | 6351 | 17211 | 36.90% |
| 177 | Saukkonen | | 2012 | Finland | 2005-2009 | NM | mixed | TS | >70 | 196 | 539 | 36.42% |
| 178 | Belfki | | 2013 | Tunisia | 2005-2009 | mixed | ATP-III | TS | 35-74 | 2220 | 4654 | 47.70% |
| 179 | Brutto | | 2013 | Ecuador | 2010-2014 | rural | IDF | TS | ≥40 | 388 | 517 | 75.10% |
| 180 | Esmailzadehha | | 2013 | Iran | 2010-2014 | NM | mixed | TS | 20-78 | 471 | 1107 | 42.58% |
| 181 | Gundogan | | 2013 | Turkey | NM | mixed | ATP-III | TS | 20-83 | 1858 | 4309 | 43.12% |
| 182 | Kumbasar | | 2013 | Turkey | 2000-2004 | urban | ATP-III | TS | ≥20 | 302 | 1106 | 27.31% |
| 183 | Lakshmipriya | | 2013 | India | NM | mixed | mixed | TS | ≥20 | 947 | 1875 | 50.51% |
| 184 | Li | | 2013 | China | NM | mixed | mixed | TS | ≥20 | 2725 | 6614 | 41.20% |
| 185 | Li | | 2013 | China | NM | urban | IDF | TS | 18-75 | 1315 | 4645 | 28.31% |
| 186 | Lim | | 2013 | USA | 1985-1999 | NM | ATP-III | TS | 15-19 | 478 | 4672 | 10.23% |
| 187 | Marcuello | | 2013 | Spain | 2010-2014 | NM | Harmonized | TS | ≥18 | 2558 | 4727 | 54.11% |
| 188 | Peer | | 2013 | South Africa | NM | urban | Harmonized | NM | 25-74 | 687 | 1099 | 62.49% |
| 189 | Pessinaba | | 2013 | Senegal | 2010-2014 | NM | mixed | TS | 15-96 | 583 | 1424 | 40.95% |
| 190 | Pimenta | | 2013 | Brazil | 2005-2009 | rural | ATP-III | TS | ≥18 | 62 | 534 | 11.61% |
| 191 | Rodriguez | | 2013 | USA | 2005-2009 | urban | ATP-III | TS | 45-51 | 3840 | 6843 | 56.11% |
| 192 | Schmitt | | 2013 | Brazil | 2005-2009 | NM | ATP-III | TS | 35-65 | 352 | 581 | 60.60% |
| 193 | Shahbazian | | 2013 | Iran | NM | urban | ATP-III | TS | 20-70 | 268 | 912 | 29.36% |
| 194 | Shalini | | 2013 | India | NM | mixed | ATP-III | TS | ≥18 | 454 | 1023 | 44.38% |
| 195 | Sherpa | | 2013 | China | NM | rural | IDF | TS | 30-80 | 290 | 692 | 41.91% |
| 196 | ES Lim | | 2013 | South Korea | 2005-2009 | NM | NHLBI | TS | ≥40 | 157224 | 690283 | 22.78% |
| 197 | Tsou | | 2013 | China | NM | urban | ATP-III | TS | ≥65 | 1072 | 2362 | 45.40% |
| 198 | Wang | | 2013 | China | NM | urban | mixed | TS | NM | 11486 | 22457 | 51.14% |
| 199 | Xi | | 2013 | China | NM | NM | mixed | TS | ≥18 | 3329 | 7488 | 44.46% |
| 200 | Zhang | | 2013 | China | NM | mixed | Harmonized | TS | 18-76 | 8475 | 19003 | 44.60% |
| 201 | Al-Thani | | 2014 | Qatar | 2010-2014 | urban | IDF | TS | 18-64 | 1087 | 2335 | 46.55% |
| 202 | Bermúdez | | 2014 | Venezuela | 2010-2014 | NM | mixed | TS | ≥18 | 1383 | 2230 | 62.00% |
| 203 | Binh | | 2014 | Vietnam | 2010-2014 | mixed | IDF | TS | 40-64 | 300 | 2443 | 12.30% |
| 204 | Brini | | 2014 | Morocco | 2010-2014 | NM | IDF | TS | ≥19 | 403 | 820 | 49.15% |
| 205 | Daghri | | 2014 | Saudi Arabia | 2005-2009 | NM | ATP-III | TS | 18-70 | 4086 | 9164 | 44.59% |
| 206 | Deedwania | | 2014 | India | 2005-2009 | urban | Harmonized | TS | NM | 2817 | 6198 | 45.45% |
| 207 | Hu | | 2014 | China | NM | NM | mixed | TS | ≥18 | 1450 | 3417 | 42.42% |
| 208 | Kang | | 2014 | South Korea | 2010-2014 | NM | ATP-III | TS | ≥20 | 2868480 | 10253085 | 27.98% |
| 209 | Mamun | | 2014 | Bangladesh | NM | rural | mixed | TS | ≥15 | 149 | 1485 | 10.03% |
| 210 | Moreira | | 2014 | Brazil | 2005-2009 | urban | ATP-III | TS | ≥18 | 1129 | 1369 | 82.50% |
| 211 | RG. Sy | | 2014 | Philippines | NM | mixed | mixed | TS | 20-50 | 1057 | 3072 | 34.40% |
| 212 | Salas | | 2014 | Mexico | 2010-2014 | NM | IDF | TS | ≥16 | 930 | 1200 | 77.50% |
| 213 | T Enkh-Oyun | | 2014 | Mongolia | NM | mixed | IDF | TS | 20-50 | 1229 | 1911 | 64.31% |
| 214 | Xu | | 2014 | China | NM | mixed | Harmonized | TS | ≥20 | 1000 | 2357 | 42.43% |
| 215 | Yu | | 2014 | China | NM | rural | ATP-III | TS | ≥35 | 4920 | 11496 | 42.80% |
| 216 | Zhao | | 2014 | China | NM | rural | mixed | TS | NM | 410 | 2990 | 13.70% |
| 217 | Amarasinghe | | 2015 | Sri Lanka | NM | mixed | ATP-III | TS | >18 | 83 | 511 | 16.24% |
| 218 | Karimi | | 2015 | Iran | 2010-2014 | rural | mixed | TS | >20 | 4870 | 13304 | 36.60% |
| 219 | Khan | | 2015 | USA | 2000-2004 | urban | ATP-III | TS | >20 | 3288 | 5227 | 62.90% |
| 220 | Khan | | 2015 | USA | 2010-2014 | NM | Harmonized | TS | 20-68 | 206 | 401 | 51.37% |
| 221 | Kosa | | 2015 | Hungary | 2010-2014 | NM | IDF | TS | >20 | 1466 | 2188 | 67.00% |
| 222 | Li | | 2015 | China | NM | mixed | Harmonized | TS | >40 | 14599 | 21128 | 69.10% |
| 223 | Obeidat | | 2015 | Jordan | NM | NM | IDF | TS | 20-70 | 451 | 630 | 71.60% |
| 224 | Park | | 2015 | South Korea | 2010-2014 | NM | Joint Scientific Statement | TS | >20 | 1977 | 5760 | 34.32% |
| 225 | Park | | 2015 | South Korea | 2010-2014 | NM | ATP-III | TS | >20 | 808 | 2213 | 36.51% |
| 226 | Peer | | 2015 | South Africa | 2005-2009 | NM | Harmonized | TS | 20-74 | 687 | 1099 | 62.50% |
| 227 | Romero | | 2015 | Mexico | 2010-2014 | NM | IDF | TS | 17-24 | 2551 | 6063 | 42.08% |
| 228 | Song | | 2015 | China | 2005-2009 | urban | NHLBI | TS | 18-74 | 6485 | 15477 | 41.90% |
| 229 | Strand | | 2015 | China | 2010-2014 | NM | ATP-III | TS | 44-56 | 731 | 791 | 92.36% |
| 230 | Tao | | 2015 | China | 2010-2014 | mixed | mixed | TS | 18-95 | 4261 | 8380 | 50.85% |
| 231 | Dhaheri | | 2016 | United Arab Emirates | 2010-2014 | NM | IDF | TS | 17-25 | 101 | 555 | 18.20% |
| 232 | Ding | | 2016 | China | 2010-2014 | NM | mixed | TS | 18-90 | 1001 | 4580 | 21.87% |
| 233 | Franca | | 2016 | Brazil | 2010-2014 | NM | mixed | TS | ≥18 | 435 | 787 | 55.30% |
| 234 | Guo | | 2016 | China | 2010-2014 | mixed | mixed | TS | NM | 1844 | 3902 | 47.26% |
| 235 | Houti | | 2016 | Algeria | 2005-2009 | urban | ATP-III | NM | 30-64 | 233 | 774 | 30.10% |
| 236 | Jiang | | 2016 | China | 2005-2009 | mixed | Harmonized | TS | 18-80 | 439 | 1042 | 42.13% |
| 237 | Kim | | 2016 | South Korea | 2010-2014 | Mixed | NHLBI | TS | ≥65 | 1512 | 3917 | 38.60% |
| 238 | Krishnadath | | 2016 | Suriname | 2010-2014 | NM | Harmonized | TS | 20-65 | 1519 | 2646 | 57.41% |
| 239 | Larrad | | 2016 | Spain | 2000-2004 | mixed | Harmonized | TS | 35-74 | 1623 | 3844 | 42.22% |
| 240 | Lee | | 2016 | South Korea | 2005-2009 | NM | ATP-III | TS | 66 | 59207 | 103763 | 57.06% |
| 241 | Liu | | 2016 | China | 2005-2009 | rural | mixed | TS | NM | 1052 | 6997 | 15.04% |
| 242 | Parini | | 2016 | Italy; Croatia | NM | rural | ATP-III | NM | NM | 1504 | 1839 | 81.78% |
| 243 | Soca | | 2016 | Cuba | 2005-2009 | NM | ATP-III | TS | ≥20 | 986 | 2085 | 47.29% |
| 244 | Soysal | | 2016 | Turkey | 2005-2009 | NM | mixed | NM | ≥30 | 6317 | 12876 | 49.06% |
| 245 | Suliga | | 2016 | Poland | 2010-2014 | mixed | IDF |  | 37-66 | 9710 | 12784 | 75.95% |
| 246 | Voevoda | | 2016 | Russia | 2010-2014 | NM | ARRSC | NM | 25-45 | 321 | 754 | 42.57% |
| 247 | Barros | | 2017 | Brazil | 2010-2014 | NM | IDF | TS | ≥19 | 545 | 1376 | 39.61% |
| 248 | Fontanelli | | 2017 | Brazil | 2010-2014 | urban | Harmonized | TS | ≥20 | 406 | 591 | 68.70% |
| 249 | Goetzel | | 2017 | USA | 2010-2014 | NM | NHLBI | TS | 18-64 | 8705 | 10018 | 86.89% |
| 250 | Hosseini | | 2017 | Iran | 2000-2004 | mixed | JIS | TS | ≥35 | 655 | 1994 | 32.85% |
| 251 | Lu | | 2017 | China | 2010-2014 | mixed | ATP-III | TS | ≥18 | 31751 | 97098 | 32.70% |
| 252 | Moore | | 2017 | USA | 1985-2004&2010-2014 | NM | Joint Scientific Statement | TS | ≥18 | 22086 | 51371 | 42.99% |
| 253 | Noshad | | 2017 | Iran | 2005-2014 | mixed | IDF | TS | 25-64 | 4458 | 8733 | 51.05% |
| 254 | Orces | | 2017 | Ecuador | 2000-2004 | mixed | IDF | NM | ≥60 | 1969 | 2568 | 76.67% |
| 255 | Owolabi | | 2017 | South Africa | NM | urban | mixed | TS | ≥18 | 615 | 998 | 61.62% |
| 256 | Raposo | | 2017 | Portugal | 2005-2009 | mixed | HARM | TS | ≥18 | 2028 | 3977 | 50.99% |
| 257 | Rodriguez | | 2017 | Mexico | 2005-2009 | NM | mixed | TS | 20-60 | 428 | 516 | 82.95% |
| 258 | Slagter | | 2017 | Netherlands | 2010-2014 | NM | ATP-III | TS | 18-79 | 45639 | 74531 | 61.23% |
| 259 | Tran | | 2017 | South Korea | 2005-2014 | NM | mixed | TS | ≥20 | 11185 | 34587 | 32.34% |
|  | | ARRSC, All-Russian Research Society of Cardiologists; ATP-III, Adult Treatment Panel III;  CDS, Chinese Diabetes Society; IDF, International Diabetes Federation; JIS, A Joint Interim Statement;  J-MS, Japanese Metabolic Syndrome; NHLBI, National Heart, Lung, and Blood Institute;  WHO, World Health Organization; NM, not mentioned; WC, Waist Circumference; TS, Trained Staff. | | | | | | | | | | |
|  | |  |  |  |  |  |  |  |  |  |  |  |

| TS |
| --- |

**Table References**

1. Ford ES, Giles WH, Dietz WH. Prevalence of the metabolic syndrome among US adults: findings from the third National Health and Nutrition Examination Survey. *JAMA* 2002; **287**(3): 356-9.

2. Al-Lawati JA, Mohammed AJ, Al-Hinai HQ, Jousilahti P. Prevalence of the metabolic syndrome among Omani adults. *Diabetes Care* 2003; **26**(6): 1781-5.

3. Azizi F, Salehi P, Etemadi A, Zahedi-Asl S. Prevalence of metabolic syndrome in an urban population: Tehran Lipid and Glucose Study. *Diabetes Research & Clinical Practice* 2003; **61**(1): 29-37.

4. Ford ES, Giles WH. A comparison of the prevalence of the metabolic syndrome using two proposed definitions. *Diabetes Care* 2003; **26**(3): 575-81.

5. Park YW, Zhu S, Palaniappan L, Heshka S, Carnethon MR, Heymsfield SB. The metabolic syndrome: prevalence and associated risk factor findings in the US population from the Third National Health and Nutrition Examination Survey, 1988-1994. *Archives of Internal Medicine* 2003; **163**(4): 427-36.

6. Tanchoco CC, Cruz AJ, Duante CA, Litonjua AD. Prevalence of metabolic syndrome among Filipino adults aged 20 years and over. *Asia Pacific Journal of Clinical Nutrition* 2003; **12**(3): 271-6.

7. Chuang SY, Chen CH, Chou P. Prevalence of metabolic syndrome in a large health check-up population in Taiwan. *Journal of the Chinese Medical Association: JCMA* 2004; **67**(12): 611-20.

8. Farrell SW, Cheng YJ, Blair SN. Prevalence of the metabolic syndrome across cardiorespiratory fitness levels in women. *Obesity Research* 2004; **12**(5): 824-30.

9. Ford ES, Giles WH, Mokdad AH. Increasing prevalence of the metabolic syndrome among u.s. Adults. *Diabetes Care* 2004; **27**(10): 2444-9.

10. Gupta R, Deedwania PC, Gupta A, Rastogi S, Panwar RB, Kothari K. Prevalence of metabolic syndrome in an Indian urban population. *International Journal of Cardiology* 2004; **97**(2): 257-61.

11. Ilanne-Parikka P, Eriksson JG, Lindstrom J, et al. Prevalence of the metabolic syndrome and its components: findings from a Finnish general population sample and the Diabetes Prevention Study cohort. *Diabetes Care* 2004; **27**(9): 2135-40.

12. Jia WP, Xiang KS, Chen L, et al. [A comparison of the application of two working definitions of metabolic syndrome in Chinese population]. *Chung-Hua i Hsueh Tsa Chih [Chinese Medical Journal]* 2004; **84**(7): 534-8.

13. Jorgensen ME, Bjerregaard P, Gyntelberg F, Borch-Johnsen K, Greenland Population S. Prevalence of the metabolic syndrome among the Inuit in Greenland. A comparison between two proposed definitions. *Diabetic Medicine* 2004; **21**(11): 1237-42.

14. Kim MH, Kim MK, Choi BY, Shin YJ. Prevalence of the metabolic syndrome and its association with cardiovascular diseases in Korea. *Journal of Korean Medical Science* 2004; **19**(2): 195-201.

15. Oh JY, Hong YS, Sung YA, Barrett-Connor E. Prevalence and factor analysis of metabolic syndrome in an urban Korean population. *Diabetes Care* 2004; **27**(8): 2027-32.

16. Santos AC, Lopes C, Barros H. Prevalence of metabolic syndrome in the city of Porto. *Revista Portuguesa de Cardiologia* 2004; **23**(1): 45-52.

17. Al-Nozha M, Al-Khadra A, Arafah MR, et al. Metabolic syndrome in Saudi Arabia. *Saudi Medical Journal* 2005; **26**(12): 1918-25.

18. Athyros VG, Bouloukos VI, Pehlivanidis AN, et al. The prevalence of the metabolic syndrome in Greece: the MetS-Greece Multicentre Study. *Diabetes, Obesity & Metabolism* 2005; **7**(4): 397-405.

19. Bo S, Gentile L, Ciccone G, et al. The metabolic syndrome and high C-reactive protein: prevalence and differences by sex in a southern-European population-based cohort. *Diabetes/Metabolism Research Reviews* 2005; **21**(6): 515-24.

20. Boronat M, Chirino R, Varillas VF, et al. Prevalence of the metabolic syndrome in the island of Gran Canaria: comparison of three major diagnostic proposals. *Diabetic Medicine* 2005; **22**(12): 1751-6.

21. Choi SH, Ahn CW, Cha BS, et al. The prevalence of the metabolic syndrome in Korean adults: comparison of WHO and NCEP criteria. *Yonsei Medical Journal* 2005; **46**(2): 198-205.

22. Dekker JM, Girman C, Rhodes T, et al. Metabolic syndrome and 10-year cardiovascular disease risk in the Hoorn Study. *Circulation* 2005; **112**(5): 666-73.

23. Florez H, Silva E, Fernandez V, et al. Prevalence and risk factors associated with the metabolic syndrome and dyslipidemia in White, Black, Amerindian and Mixed Hispanics in Zulia State, Venezuela. *Diabetes Research & Clinical Practice* 2005; **69**(1): 63-77.

24. Grandinetti A, Chang HK, Theriault A, Mor J. Metabolic syndrome in a multiethnic population in rural Hawaii. *Ethnicity & Disease* 2005; **15**(2): 233-7.

25. Guerrero-Romero F, Rodriguez-Moran M. Concordance between the 2005 International Diabetes Federation definition for diagnosing metabolic syndrome with the National Cholesterol Education Program Adult Treatment Panel III and the World Health Organization definitions. *Diabetes Care* 2005; **28**(10): 2588-9.

26. Ko GT, Cockram CS, Chow CC, et al. High prevalence of metabolic syndrome in Hong Kong Chinese--comparison of three diagnostic criteria. *Diabetes Research & Clinical Practice* 2005; **69**(2): 160-8.

27. Li R, Zhang SH, Ren W, et al. Epidemiological study on the metabolic syndrome and related factors in the elderly of Chongqing area. [Chinese]. *Chinese Journal of Clinical Rehabilitation* 2005; **9**(11): 1-3.

28. Miccoli R, Bianchi C, Odoguardi L, et al. Prevalence of the metabolic syndrome among Italian adults according to ATP III definition. *Nutrition Metabolism & Cardiovascular Diseases* 2005; **15**(4): 250-4.

29. Rosenbaum P, Gimeno SGA, Sanudo A, et al. Analysis of criteria for metabolic syndrome in a population-based study of Japanese-Brazilians. *Diabetes, Obesity and Metabolism* 2005; **7**(4): 352-9.

30. Son le NT, Kunii D, Hung NT, Sakai T, Yamamoto S. The metabolic syndrome: prevalence and risk factors in the urban population of Ho Chi Minh City. *Diabetes Research & Clinical Practice* 2005; **67**(3): 243-50.

31. Tanaka H, Shimabukuro T, Shimabukuro M. High prevalence of metabolic syndrome among men in Okinawa. *Journal of Atherosclerosis & Thrombosis* 2005; **12**(5): 284-8.

32. Thomas GN, Ho SY, Janus ED, et al. The US National Cholesterol Education Programme Adult Treatment Panel III (NCEP ATP III) prevalence of the metabolic syndrome in a Chinese population. *Diabetes Research & Clinical Practice* 2005; **67**(3): 251-7.

33. Tillin T, Forouhi N, Johnston DG, McKeigue PM, Chaturvedi N, Godsland IF. Metabolic syndrome and coronary heart disease in South Asians, African-Caribbeans and white Europeans: a UK population-based cross-sectional study. *Diabetologia* 2005; **48**(4): 649-56.

34. Urashima M, Wada T, Fukumoto T, et al. Prevalence of metabolic syndrome in a 22,892 japanese population and its association with life style. *Japan Medical Association Journal* 2005; **48**(9): 441-50.

35. Yan W, Yang X, Zheng Y, et al. The metabolic syndrome in Uygur and Kazak populations. *Diabetes Care* 2005; **28**(10): 2554-5.

36. Zhang G, Zhu ZM, Zhao ZG, et al. Comparative study of different diagnostic criteria of metabolic syndrome. [Chinese]. *Zhonghua yi xue za zhi* 2005; **85**(7): 490-1.

37. Al-Lawati JA, Jousilahti P. Prevalence of metabolic syndrome in Oman using the International Diabetes Federation's Criteria. *Saudi Medical Journal* 2006; **27**(12): 1925-6.

38. Al-Qahtani DA, Imtiaz ML, Saad OS, Hussein NM. A comparison of the prevalence of metabolic syndrome in Saudi adult females using two definitions. *Metabolic Syndrome and Related Disorders* 2006; **4**(3): 204-14.

39. Arai H, Yamamoto A, Matsuzawa Y, et al. Prevalence of metabolic syndrome in the general Japanese population in 2000. *Journal of Atherosclerosis & Thrombosis* 2006; **13**(4): 202-8.

40. Bouguerra R, Ben Salem L, Alberti H, et al. Prevalence of metabolic abnormalities in the Tunisian adults: a population based study. *Diabetes & Metabolism* 2006; **32**(3): 215-21.

41. Fakhrzadeh H, Ebrahimpour P, Pourebrahim R, Heshmat R, Larijani B. Metabolic syndrome and its associated risk factors in healthy adults: A population-based study in Iran. *Metabolic Syndrome and Related Disorders* 2006; **4**(1): 28-34.

42. Feng Y, Hong X, Li Z, et al. Prevalence of metabolic syndrome and its relation to body composition in a Chinese rural population. *Obesity* 2006; **14**(11): 2089-98.

43. Gause-Nilsson I, Gherman S, Kumar Dey D, Kennerfalk A, Steen B. Prevalence of metabolic syndrome in an elderly Swedish population. *Acta Diabetologica* 2006; **43**(4): 120-6.

44. Harzallah F, Alberti H, Ben Khalifa F. The metabolic syndrome in an Arab population: a first look at the new International Diabetes Federation criteria. *Diabetic Medicine* 2006; **23**(4): 441-4.

45. He Y, Jiang B, Wang J, et al. Prevalence of the metabolic syndrome and its relation to cardiovascular disease in an elderly Chinese population. *Journal of the American College of Cardiology* 2006; **47**(8): 1588-94.

46. Hu XS, Guo ZR, Zhou H, et al. [Study on the prevalence of metabolic syndrome among 35-74 year-olds in Jiangsu province]. *Chung-Hua Liu Hsing Ping Hsueh Tsa Chih Chinese Journal of Epidemiology* 2006; **27**(9): 751-6.

47. Ko GT, Cockram CS, Chow CC, et al. Metabolic syndrome by the international diabetes federation definition in Hong Kong Chinese. *Diabetes Research & Clinical Practice* 2006; **73**(1): 58-64.

48. Liu J, Grundy SM, Smith Jr SC, et al. Ethnic-specific criteria for the metabolic syndrome: Evidence from China. *Diabetes Care* 2006; **29**(6): 1414-6.

49. Liu J, Hanley AJ, Young TK, Harris SB, Zinman B. Characteristics and prevalence of the metabolic syndrome among three ethnic groups in Canada. *International Journal of Obesity* 2006; **30**(4): 669-76.

50. Lu W, Liu MX, Li R, Fu H, Jin TY, Zhang SN. [Epidemiological feature of metabolic syndrome in Shanghai residents aged 15 - 74 years]. *Chung-Hua Yu Fang i Hsueh Tsa Chih [Chinese Journal of Preventive Medicine]* 2006; **40**(4): 262-8.

51. Miyatake N, Kawasaki Y, Nishikawa H, Takenami S, Numata T. Prevalence of metabolic syndrome in Okayama prefecture, Japan. *Internal Medicine* 2006; **45**(2): 107-8.

52. Park HS, Lee SY, Kim SM, Han JH, Kim DJ. Prevalence of the metabolic syndrome among Korean adults according to the criteria of the International Diabetes Federation. *Diabetes Care* 2006; **29**(4): 933-4.

53. Patel A, Huang KC, Janus ED, et al. Is a single definition of the metabolic syndrome appropriate?--A comparative study of the USA and Asia. *Atherosclerosis* 2006; **184**(1): 225-32.

54. Ravaglia G, Forti P, Maioli F, et al. Metabolic Syndrome: prevalence and prediction of mortality in elderly individuals. *Diabetes Care* 2006; **29**(11): 2471-6.

55. Seclen S, Villena A, Martinez Larrad MT, et al. Prevalence of the metabolic syndrome in the mestizo population of Peru. *Metabolic Syndrome and Related Disorders* 2006; **4**(1): 1-6.

56. Szurkowska M, Szafraniec K, Gilis-Januszewska A, et al. [Prevalence of the metabolic syndrome and its components in adult inhabitants of Krakow]. *Przeglad Lekarski* 2006; **63**(9): 733-7.

57. Deepa M, Farooq S, Datta M, Deepa R, Mohan V. Prevalence of metabolic syndrome using WHO, ATPIII and IDF definitions in Asian Indians: the Chennai Urban Rural Epidemiology Study (CURES-34). *Diabetes/Metabolism Research Reviews* 2007; **23**(2): 127-34.

58. Group DS. Prevalence of the metabolic syndrome in populations of Asian origin. Comparison of the IDF definition with the NCEP definition. *Diabetes Research & Clinical Practice* 2007; **76**(1): 57-67.

59. Hadaegh F, Zabetian A, Azizi F. Prevalence of metabolic syndrome in Iranian adult population, concordance between the IDF with the ATPIII and the WHO definitions. [Polish]. *Iranian Journal of Diabetes and Lipid Disorders* 2007; **6**(4): 59-67+E44.

60. Hildrum B, Mykletun A, Hole T, Midthjell K, Dahl AA. Age-specific prevalence of the metabolic syndrome defined by the International Diabetes Federation and the National Cholesterol Education Program: the Norwegian HUNT 2 study. *BMC Public Health* 2007; **7**: 220.

61. Hwang LC, Bai CH, Chen CJ, Chien KL. Gender difference on the development of metabolic syndrome: A population-based study in Taiwan. *European Journal of Epidemiology* 2007; **22**(12): 899-906.

62. Khader Y, Bateiha A, El-Khateeb M, Al-Shaikh A, Ajlouni K. High prevalence of the metabolic syndrome among Northern Jordanians. *Journal of Diabetes & its Complications* 2007; **21**(4): 214-9.

63. Kim HM, Kim DJ, Jung IH, Park C, Park J. Prevalence of the metabolic syndrome among Korean adults using the new International Diabetes Federation definition and the new abdominal obesity criteria for the Korean people. *Diabetes Research & Clinical Practice* 2007; **77**(1): 99-106.

64. Kozan O, Oguz A, Abaci A, et al. Prevalence of the metabolic syndrome among Turkish adults. *European Journal of Clinical Nutrition* 2007; **61**(4): 548-53.

65. Li Y, Zhao D, Wang W, et al. [A comparison of three diagnostic criterions for metabolic syndrome applied in a Chinese population aged 35-64 in 11 provinces]. *Chung-Hua Liu Hsing Ping Hsueh Tsa Chih Chinese Journal of Epidemiology* 2007; **28**(1): 83-7.

66. Lin CC, Liu CS, Lai MM, et al. Metabolic syndrome in a Taiwanese metropolitan adult population. *BMC Public Health* 2007; **7**: 239.

67. Mattsson N, Ronnemaa T, Juonala M, Viikari JS, Raitakari OT. The prevalence of the metabolic syndrome in young adults. The Cardiovascular Risk in Young Finns Study. *Journal of Internal Medicine* 2007; **261**(2): 159-69.

68. Park HS, Kim SM, Lee JS, et al. Prevalence and trends of metabolic syndrome in Korea: Korean National Health and Nutrition Survey 1998-2001. *Diabetes, Obesity & Metabolism* 2007; **9**(1): 50-8.

69. Prabhakaran D, Chaturvedi V, Shah P, et al. Differences in the prevalence of metabolic syndrome in urban and rural India: a problem of urbanization. *Chronic Illness* 2007; **3**(1): 8-19.

70. Sandhofer A, Iglseder B, Paulweber B, Ebenbichler CF, Patsch JR. Comparison of different definitions of the metabolic syndrome. *European Journal of Clinical Investigation* 2007; **37**(2): 109-16.

71. Santos AC, Barros H. Impact of metabolic syndrome definitions on prevalence estimates: a study in a Portuguese community. *Diabetes & Vascular Disease Research* 2007; **4**(4): 320-7.

72. Yang W, Reynolds K, Gu D, Chen J, He J. A comparison of two proposed definitions for metabolic syndrome in the Chinese adult population. *American Journal of the Medical Sciences* 2007; **334**(3): 184-9.

73. Yoon YS, Lee ES, Park C, Lee S, Oh SW. The new definition of metabolic syndrome by the international diabetes federation is less likely to identify metabolically abnormal but non-obese individuals than the definition by the revised national cholesterol education program: the Korea NHANES study. *International Journal of Obesity* 2007; **31**(3): 528-34.

74. Bindraban NR, van Valkengoed IG, Mairuhu G, et al. A new tool, a better tool? Prevalence and performance of the International Diabetes Federation and the National Cholesterol Education Program criteria for metabolic syndrome in different ethnic groups. *European Journal of Epidemiology* 2008; **23**(1): 37-44.

75. Erem C, Hacihasanoglu A, Deger O, et al. Prevalence of metabolic syndrome and associated risk factors among Turkish adults: Trabzon MetS study.[Erratum appears in Endocrine. 2008 Aug;35(2):269]. *Endocrine* 2008; **33**(1): 9-20.

76. Fiuza M, Cortez-Dias N, Martins S, Belo A. Metabolic syndrome in Portugal: Prevalence and implications for cardiovascular risk - Results from the VALSIM Study [107]. [Portuguese, English]

Sindrome metabolica em Portugal: Prevalencia e implicacoes no risco cardiovascular - Resultados do estudo VALSIM [107]. *Revista Portuguesa de Cardiologia* 2008; **27**(12): 1495-529.

77. Hu G, Lindstrom J, Jousilahti P, et al. The increasing prevalence of metabolic syndrome among Finnish men and women over a decade. *Journal of Clinical Endocrinology & Metabolism* 2008; **93**(3): 832-6.

78. Kelliny C, William J, Riesen W, Paccaud F, Bovet P. Metabolic syndrome according to different definitions in a rapidly developing country of the African region. *Cardiovascular Diabetology* 2008; **7**: 27.

79. Lopez Suarez A, Elvira Gonzalez J, Beltran Robles M, et al. [Prevalence of obesity, diabetes, hypertension, hypercholesterolemia and metabolic syndrome in over 50-year-olds in Sanlucar de Barrameda, Spain]. *Revista Espanola de Cardiologia* 2008; **61**(11): 1150-8.

80. Maggi S, Noale M, Zambon A, et al. Validity of the ATP III diagnostic criteria for the metabolic syndrome in an elderly Italian Caucasian population: the Italian Longitudinal Study on Aging. *Atherosclerosis* 2008; **197**(2): 877-82.

81. Malik M, Razig SA. The prevalence of the metabolic syndrome among the multiethnic population of the United Arab Emirates: a report of a national survey. *Metabolic Syndrome & Related Disorders* 2008; **6**(3): 177-86.

82. Marquezine GF, Oliveira CM, Pereira AC, Krieger JE, Mill JG. Metabolic syndrome determinants in an urban population from Brazil: social class and gender-specific interaction. *International Journal of Cardiology* 2008; **129**(2): 259-65.

83. Moebus S, Hanisch J, Bramlage P, et al. Regional differences in the prevalence of the metabolic syndrome in primary care practices in Germany. [German]

Regional unterschiedliche pravalenz des metabolischen syndroms: Daten zur primararztlichen versorgung in Deutschland. *Deutsches Arzteblatt* 2008; **105**(12): 207-13.

84. Mokan M, Galajda P, Pridavkova D, et al. Prevalence of diabetes mellitus and metabolic syndrome in Slovakia. *Diabetes Research & Clinical Practice* 2008; **81**(2): 238-42.

85. Neuhauser H, Ellert U. Estimation of the metabolic syndrome prevalence in the general population in Germany. *Journal of Public Health* 2008; **16**(3): 221-7.

86. Park HS, Park CY, Oh SW, Yoo HJ. Prevalence of obesity and metabolic syndrome in Korean adults. *Obesity Reviews* 2008; **9**(2): 104-7.

87. Park J, Mendoza JA, O'Neil CE, Hilmers DC, Liu Y, Nicklas TA. A comparison of the prevalence of the metabolic syndrome in the United States (US) and Korea in young adults aged 20 to 39 years. *Asia Pacific Journal of Clinical Nutrition* 2008; **17**(3): 471-82.

88. Perez CM, Guzman M, Ortiz AP, et al. Prevalence of the metabolic syndrome in San Juan, Puerto Rico. *Ethnicity and Disease* 2008; **18**(4): 434-41.

89. Ramos RG, Olden K. The prevalence of metabolic syndrome among US women of childbearing age. *American Journal of Public Health* 2008; **98**(6): 1122-7.

90. Sun F, Tao QS, Zhan SY. [Comparison of five different diagnostic criteria on metabolic syndrome applied during physical check-up programs among population aged 35-74, in Taiwan]. *Chung-Hua Liu Hsing Ping Hsueh Tsa Chih Chinese Journal of Epidemiology* 2008; **29**(9): 925-9.

91. Surana SP, Shah DB, Gala K, et al. Prevalence of metabolic syndrome in an urban Indian diabetic population using the NCEP ATP III guidelines. *Journal of the Association of Physicians of India* 2008; **56**: 865-8.

92. Welin L, Adlerberth A, Caidahl K, et al. Prevalence of cardiovascular risk factors and the metabolic syndrome in middle-aged men and women in Gothenburg, Sweden. *BMC Public Health* 2008; **8**: 403.

93. Arikan I, Metintas S, Kalyoncu C, Colak O, Arikan U. Evaluation of metabolic syndrome prevalence in semi-rural areas of Central Anatolia, Turkey. *Saudi Medical Journal* 2009; **30**(8): 1073-80.

94. Bener A. Prevalence of metabolic syndrome according to ATP III and IDF criteria : A population based study. *Journal of Diabetes* 2009; **1**: A86.

95. Buckland GG, Salas-Salvado J, Serra-Majem L, Castell C, Cabre J, Salleras-Sanmarti L. Increase in metabolic syndrome as defined by ATPIII from 1992-1993 to 2002-2003 in a Mediterranean population. *Nutrition Reviews* 2009; **67 Suppl 1**: S117-25.

96. Can AS, Ozbayrakci S, Palaoglu KE, Bersot TP. Comparison between Turkish Cardiovascular Risk Platform and United States National Cholesterol Education Program Adult Treatment Panel III definitions of the metabolic syndrome in Turkish adults. *Journal of the CardioMetabolic Syndrome* 2009; **4**(1): 26-32.

97. Cardenas Quintana H, Sanchez Abanto J, Roldan Arbieto L, Mendoza Tasayco F. [Prevalence of metabolic syndrome in people 20 years old and more. Peru, 2005]. *Revista Espanola de Salud Publica* 2009; **83**(2): 257-65.

98. Delavar MA, Lye MS, Khor GL, Hanachi P, Hassan STBS. Prevalence of Metabolic syndrome among middle aged women in Babol, Iran. *Southeast Asian Journal of Tropical Medicine and Public Health* 2009; **40**(3): 612-28.

99. Ekelund U, Anderssen S, Andersen LB, et al. Prevalence and correlates of the metabolic syndrome in a population-based sample of European youth. *American Journal of Clinical Nutrition* 2009; **89**(1): 90-6.

100. Elasmi M, Zayani Y, Hadj Taieb S, et al. Prevalence of the metabolic syndrome among adults in Tunisia. *Journal of Diabetes* 2009; **1**: A88.

101. Ervin RB. Prevalence of metabolic syndrome among adults 20 years of age and over, by sex, age, race and ethnicity, and body mass index: United States, 2003-2006. *National health statistics reports* 2009; (13): 1-7.

102. Fiuza MM, Cortez-Dias N, Martins S, Belo A. Obesity, waist circumference and metabolic syndrome in the Portuguese population. Insights of the VALSIM study. *European Heart Journal* 2009; **30**: 442.

103. Gelaye B, Revilla L, Lopez T, Sanchez S, Williams MA. Prevalence of metabolic syndrome and its relationship with leisure time physical activity among Peruvian adults. *European Journal of Clinical Investigation* 2009; **39**(10): 891-8.

104. Gundogan K, Bayram F, Capak M, et al. Prevalence of metabolic syndrome in the mediterranean region of Turkey: Evaluation of hypertension, diabetes mellitus, obesity, and dyslipidemia. *Metabolic Syndrome and Related Disorders* 2009; **7**(5): 427-34.

105. Hadaegh F, Zabetian A, Tohidi M, Ghasemi A, Sheikholeslami F, Azizi F. Prevalence of metabolic syndrome by the Adult Treatment Panel III, International Diabetes Federation, and World Health Organization definitions and their association with coronary heart disease in an elderly Iranian population. *Annals of the Academy of Medicine, Singapore* 2009; **38**(2): 142-9.

106. Rosas-Saucedo J, San Roman-Torres S, Eguia A, Rosas-Guzman J. Prevalence of metabolic syndrome in the city of Celaya, Mexico. *Canadian Journal of Diabetes* 2009; **33 (3)**: 309.

107. Sharifi F, Mousavinasab SN, Saeini M, Dinmohammadi M. Prevalence of metabolic syndrome in an adult urban population of the west of Iran. *Experimental Diabetes Research* 2009; **2009**: 136501.

108. Tao SB, Ren Y, Ran XW, et al. [Epidemiological study on metabolic syndrome in Chengdu adult in 2007]. *Sichuan da Xue Xue Bao Yi Xue Ban/Journal of Sichuan University Medical Science Edition* 2009; **40**(6): 1062-5, 126.

109. van den Donk M, Bobbink IW, Gorter KJ, Salome PL, Rutten GE. Identifying people with metabolic syndrome in primary care by screening with a mailed tape measure: a survey of 14,000 people in the Netherlands. *Preventive Medicine* 2009; **48**(4): 345-50.

110. Xu T, Zhang Y, Yu L, Tong W. Prevalence of the metabolic syndrome and its risk factors in Inner Mongolia, China. *Acta Cardiologica* 2009; **64**(3): 397-404.

111. Zuo H, Shi Z, Hu X, Wu M, Guo Z, Hussain A. Prevalence of metabolic syndrome and factors associated with its components in Chinese adults. *Metabolism: Clinical & Experimental* 2009; **58**(8): 1102-8.

112. Al-Daghri NM, Al-Attas OS, Alokail MS, Alkharfy KM, Sabico SLB, Chrousos GP. Decreasing prevalence of the full metabolic syndrome but a persistently high prevalence of dyslipidemia among adult Arabs. *PLoS ONE* 2010; **5 (8) (no pagination)**(e12159).

113. Allal-Elasmi M, Haj Taieb S, Hsairi M, et al. The metabolic syndrome: prevalence, main characteristics and association with socio-economic status in adults living in Great Tunis. *Diabetes & Metabolism* 2010; **36**(3): 204-8.

114. Bhat RA, Laway BA, Zargar AH. Prevalence of metabolic syndrome in Kashmir valley of Indian subcontinent. *Indian Journal of Medical Sciences* 2010; **64**(6): 259-64.

115. Biad A, Chibane A, Makhlouf L, Atif A, Lanasri L, Kessous L. The prevalence of the metabolic syndrome in east of algiers. *Journal of Hypertension* 2010; **28**: e133.

116. Ferguson TS, Tulloch-Reid MK, Younger NO, et al. Prevalence of the metabolic syndrome and its components in relation to socioeconomic status among Jamaican young adults: a cross-sectional study. *BMC Public Health* 2010; **10**: 307.

117. Ferguson TS, Younger N, Tulloch-Reid MK, et al. Prevalence of the metabolic syndrome in Jamaican adults and its relationship to income and education levels. *West Indian Medical Journal* 2010; **59**(3): 265-73.

118. Flowers E, Molina C, Mathur A, et al. Prevalence of metabolic syndrome in South Asians residing in the United States. *Metabolic Syndrome & Related Disorders* 2010; **8**(5): 417-23.

119. Guo H, Guo SX, Zhang JY, et al. [Study on the prevalence of metabolic syndrome among the Kazakh population in Xinjiang]. [Chinese]. *Zhonghua liu xing bing xue za zhi = Zhonghua liuxingbingxue zazhi* 2010; **31**(7): 747-50.

120. Khunti K, Taub N, Tringham J, et al. Screening for the metabolic syndrome using simple anthropometric measurements in south Asian and white Europeans: a population-based screening study. The Leicester Ethnic Atherosclerosis and Diabetes Risk (LEADER) Study. *Primary care diabetes* 2010; **4**(1): 25-32.

121. Li G, de Courten M, Jiao S, Wang Y. Prevalence and characteristics of the metabolic syndrome among adults in Beijing, China. *Asia Pacific Journal of Clinical Nutrition* 2010; **19**(1): 98-102.

122. Li JB, Wang X, Zhang JX, et al. Metabolic syndrome: prevalence and risk factors in southern China. *Journal of International Medical Research* 2010; **38**(3): 1142-8.

123. Misra R, Patel T, Kotha P, et al. Prevalence of diabetes, metabolic syndrome, and cardiovascular risk factors in US Asian Indians: results from a national study. *Journal of Diabetes & its Complications* 2010; **24**(3): 145-53.

124. Moebus S, Balijepalli C, Losch C, et al. Age- and sex-specific prevalence and ten-year risk for cardiovascular disease of all 16 risk factor combinations of the metabolic syndrome - A cross-sectional study. *Cardiovascular Diabetology* 2010; **9**: 34.

125. Oguz A, Altuntas Y, Karsidag K, et al. The prevalence of metabolic syndrome in Turkey. *Obesity Reviews* 2010; **11 (6)**: 486.

126. Oladapo OO, Salako L, Sodiq O, Shoyinka K, Adedapo K, Falase AO. A prevalence of cardiometabolic risk factors among a rural Yoruba south-western Nigerian population: a population-based survey. *Cardiovascular Journal of Africa* 2010; **21**(1): 26-31.

127. Ravikiran M, Bhansali A, Ravikumar P, et al. Prevalence and risk factors of metabolic syndrome among Asian Indians: a community survey. *Diabetes Research & Clinical Practice* 2010; **89**(2): 181-8.

128. Schipf S, Alte D, Volzke H, et al. Pravalenz of metabolic syndrome in Germany: Results from the study of health in pomerania (SHIP). [German]

Pravalenz des Metabolischen Syndroms in Deutschland: Ergebnisse der Study of Health in Pomerania (SHIP). *Diabetologie und Stoffwechsel* 2010; **5**(3): 161-8.

129. Sidorenkov O, Nilssen O, Brenn T, Martiushov S, Arkhipovsky VL, Grjibovski AM. Prevalence of the metabolic syndrome and its components in Northwest Russia: the Arkhangelsk study. *BMC Public Health* 2010; **10**: 23.

130. Valenzuela AA, Maiz A, Margozzini P, et al. [Prevalence of metabolic syndrome among Chilean adults]. *Revista Medica de Chile* 2010; **138**(6): 707-14.

131. Wang L, Tao Y, Xie Z, et al. Prevalence of metabolic syndrome, insulin resistance, impaired fasting blood glucose, and dyslipidemia in Uygur and Kazak populations. *Journal of Clinical Hypertension* 2010; **12**(9): 741-5.

132. Xavier NP, Chaim RC, Gimeno SGA, et al. Prevalence of metabolic syndrome in Japanese-Brazilians according to specific definitions for ethnicity. *Metabolic Syndrome and Related Disorders* 2010; **8**(2): 143-8.

133. Xu WH, Ruan XN, Fu XJ, et al. Prevalence of the metabolic syndrome in Pudong New Area of Shanghai using three proposed definitions among Chinese adults. *BMC Public Health* 2010; **10**: 246.

134. Zhao H, Zhu J, Wang ZH, et al. Epidemiological investigation of metabolic syndrome in 553 general adults aged 35-74 years from Sangong Community of Urumqi. [Chinese]. *Journal of Clinical Rehabilitative Tissue Engineering Research* 2010; **14**(15): 2833-7.

135. Zhao Y, Liu XY, Jin J, Yang JJ, Zhang YH. [Prevalence of metabolic syndrome among rural adult residents in Ningxia.]. [Chinese]. *Zhonghua liu xing bing xue za zhi = Zhonghua liuxingbingxue zazhi* 2010; **31**(3): 245-50.

136. Aekplakorn W, Kessomboon P, Sangthong R, et al. Urban and rural variation in clustering of metabolic syndrome components in the Thai population: results from the fourth National Health Examination Survey 2009. *BMC Public Health* 2011; **11**: 854.

137. Alkerwi A, Donneau AF, Sauvageot N, et al. Prevalence of the metabolic syndrome in Luxembourg according to the Joint Interim Statement definition estimated from the ORISCAV-LUX study. *BMC Public Health* 2011; **11**(1): 4.

138. Burazor I, Vukadinovic N, Burazor M, Burazor N, Lazovic M, Burazor Z. Metabolic syndrome prevalence and its individual risk factors characteristics in population of central Serbia. *Journal of Clinical Lipidology* 2011; **5 (3)**: 230.

139. De Oliveira GF, De Oliveira TRR, Rodrigues FF, Correa LF, De Arruda TB, Casulari LA. Prevalence of metabolic syndrome in the indigenous population, aged 19 to 69 years, from Jaguapiru Village, Dourados (MS), Brazil. *Ethnicity and Disease* 2011; **21**(3): 301-6.

140. Faraz Danish Alvi S, Zafar Iqbal Hydrie M, Fawwad A, Basit A, Riaz M, Samad Shera A. Ethnic differences in metabolic syndrome among South Asians of Pakistan. *Pakistan Journal of Medical Sciences* 2011; **27**(3): 484-9.

141. Gavrila D, Salmeron D, Egea-Caparros JM, et al. Prevalence of metabolic syndrome in Murcia Region, a southern European Mediterranean area with low cardiovascular risk and high obesity. *BMC Public Health* 2011; **11**: 562.

142. Janszky I, Vatten L, Romundstad P, et al. Metabolic syndrome in Poland - the PONS Study. *Annals of Agricultural & Environmental Medicine* 2011; **18**(2): 270-2.

143. Jiang S, Zhang L, Shao L, Yan LJ, Yldos A, Xie ZJ. [Epidemiological investigation and comparison of three different diagnostic criteria regarding metabolic syndrome among population age 30 - 80 in Xinjiang Uigur areas]. [Chinese]. *Zhonghua liu xing bing xue za zhi = Zhonghua liuxingbingxue zazhi* 2011; **32**(8): 756-9.

144. Kawada T, Otsuka T, Endo T, Kon Y. Prevalence of the metabolic syndrome and its relationship with diabetes mellitus by aging. *Aging Male* 2011; **14**(3): 203-6.

145. Li WC, Chen JY, Lin CH, Chao YJ, Chuang HH. Reevaluating the diagnostic criteria for metabolic syndrome in the Taiwanese population. *Journal of the American College of Nutrition* 2011; **30**(4): 241-7.

146. Lim S, Shin H, Song JH, et al. Increasing prevalence of metabolic syndrome in Korea: the Korean National Health and Nutrition Examination Survey for 1998-2007. *Diabetes Care* 2011; **34**(6): 1323-8.

147. Mohamud WN, Ismail AA, Sharifuddin A, et al. Prevalence of metabolic syndrome and its risk factors in adult Malaysians: results of a nationwide survey.[Erratum appears in Diabetes Res Clin Pract. 2012 Apr;96(1):90], [Reprint in Diabetes Res Clin Pract. 2012 Apr;96(1):91-7; PMID: 22553777]. *Diabetes Research & Clinical Practice* 2011; **91**(2): 239-45.

148. Motala AA, Esterhuizen T, Pirie FJ, Omar MA. The prevalence of metabolic syndrome and determination of the optimal waist circumference cutoff points in a rural South african community. *Diabetes Care* 2011; **34**(4): 1032-7.

149. Orhan H, Sadikoglu G, Ozcakir A, Bilgel NG. Metabolic syndrome among women: A study from bursa turkey

150. Pan JJ, Qu HQ, Rentfro A, McCormick JB, Fisher-Hoch SP, Fallon MB. Prevalence of metabolic syndrome and risks of abnormal serum alanine aminotransferase in Hispanics: a population-based study. *PLoS ONE [Electronic Resource]* 2011; **6**(6): e21515.

151. Pimenta AM, Gazzinelli A, Velasquez-Melendez G. [Prevalence of metabolic syndrome and its associated factors in a rural area of Minas Gerais State (MG, Brazil)]. *Ciencia & Saude Coletiva* 2011; **16**(7): 3297-306.

152. Riediger ND, Clara I. Prevalence of metabolic syndrome in the Canadian adult population. *CMAJ Canadian Medical Association Journal* 2011; **183**(15): E1127-34.

153. Sawant A, Mankeshwar R, Shah S, et al. Prevalence of metabolic syndrome in Urban India. *Cholesterol* 2011; **2011 (no pagination)**(920983).

154. Sharma SK, Ghimire A, Radhakrishnan J, et al. Prevalence of hypertension, obesity, diabetes, and metabolic syndrome in Nepal. *International Journal of Hypertension* 2011; **2011 (no pagination)**(821971).

155. Tsai CH, Li TC, Lin CC, Tsay HS. Factor analysis of modifiable cardiovascular risk factors and prevalence of metabolic syndrome in adult Taiwanese. *Endocrine* 2011; **40**(2): 256-64.

156. Wei YL, Peng X, Ma YT, et al. Relationship between the prevalence study of smoking and metabolic syndrome among the males of Uygur and Kazakh in Xinjiang. [Chinese]. *National Medical Journal of China* 2011; **91**(12): 810-4.

157. Wen CP, Chan HT, Tsai MK, et al. Attributable mortality burden of metabolic syndrome: Comparison with its individual components. *European Journal of Cardiovascular Prevention and Rehabilitation* 2011; **18**(4): 561-73.

158. Zhao J, Pang ZC, Zhang L, et al. Prevalence of metabolic syndrome in rural and urban Chinese population in Qingdao. *Journal of Endocrinological Investigation* 2011; **34**(6): 444-8.

159. Al Zenki S, Al Omirah H, Al Hooti S, et al. High prevalence of metabolic syndrome among Kuwaiti adults-a wake-up call for public health intervention. *International Journal of Environmental Research and Public Health* 2012; **9**(5): 1984-96.

160. Alzahrani AM, Karawagh AM, Alshahrani FM, Naser TA, Ahmed AA, Alsharef EH. Prevalence and predictors of metabolic syndrome among healthy Saudi Adults. *British Journal of Diabetes and Vascular Disease* 2012; **12**(2): 78-80.

161. Azimi-Nezhad M, Herbeth B, Siest G, et al. High prevalence of metabolic syndrome in Iran in comparison with France: what are the components that explain this? *Metabolic Syndrome & Related Disorders* 2012; **10**(3): 181-8.

162. Cai H, Huang J, Xu G, et al. Prevalence and determinants of metabolic syndrome among women in Chinese rural areas. *PLoS ONE [Electronic Resource]* 2012; **7**(5): e36936.

163. Cetin F, Gunes G, Ozer A. [Prevalence of metabolic syndrome, its relationship with mental health (anger) and sociodemographic characteristics in women residing in central district of Malatya: a cross-sectional observational study]. *Anadolu Kardiyoloji Dergisi* 2012; **12**(1): 53-9.

164. Deka R, Durakovic Z, Niu W, et al. Prevalence of metabolic syndrome and related metabolic traits in an island population of the Adriatic. *Annals of Human Biology* 2012; **39**(1): 46-53.

165. Fei YF, Wang C, Liu GJ, et al. [Comparison of different diagnostic criteria for metabolic syndrome in Sichuan population]. *Sichuan da Xue Xue Bao Yi Xue Ban/Journal of Sichuan University Medical Science Edition* 2012; **43**(4): 547-52.

166. Ilow R, Regulska-Ilow B, Rozanska D, Kowalisko A, Biernat J. Prevalence of metabolic syndrome among 40- and 50-year-old inhabitants of Wroclaw, Poland. *Annals of Agricultural & Environmental Medicine* 2012; **19**(3): 551-6.

167. Jesmin S, Islam MR, Islam AM, et al. Comprehensive assessment of metabolic syndrome among rural Bangladeshi women. *BMC Public Health* 2012; **12**: 49.

168. Kaduka LU, Kombe Y, Kenya E, et al. Prevalence of metabolic syndrome among an urban population in Kenya. *Diabetes Care* 2012; **35**(4): 887-93.

169. Kim TN, Kim JM, Won JC, et al. A decision tree-based approach for identifying urban-rural differences in metabolic syndrome risk factors in the adult Korean population. *Journal of Endocrinological Investigation* 2012; **35**(9): 847-52.

170. Lao XQ, Zhang YH, Wong MC, et al. The prevalence of metabolic syndrome and cardiovascular risk factors in adults in southern China. *BMC Public Health* 2012; **12**: 64.

171. Li CH, Guo SX, Ma RL, et al. [The epidemic situation of metabolic syndrome among the Uygur in Kashgar of Xinjiang in 2010]. [Chinese]. *Zhonghua yu fang yi xue za zhi [Chinese journal of preventive medicine]* 2012; **46**(5): 419-23.

172. Mahjoub S, Ahmadi MH, Faramarzi M, Ghorbani H, Moazezi Z. The prevalence of metabolic syndrome according to the Iranian Committee of Obesity and ATP III criteria in Babol, North of Iran. *Caspian Journal of Internal Medicine* 2012; **3**(2): 410-6.

173. Metelskaya VA, Shkolnikova MA, Shalnova SA, et al. Prevalence, components, and correlates of metabolic syndrome (MetS) among elderly Muscovites. *Archives of Gerontology & Geriatrics* 2012; **55**(2): 231-7.

174. Mora Garcia G, Salguedo Madrid G, Ruiz Diaz M, et al. [Agreement between Five Definitions of Metabolic Syndrome: Cartagena, Colombia]. *Revista Espanola de Salud Publica* 2012; **86**(3): 301-11.

175. Prasad DS, Kabir Z, Dash AL, Das BC. Prevalence and risk factors for metabolic syndrome in Asian Indians: A community study from urban Eastern India. *Journal of Cardiovascular Disease Research* 2012; **3**(3): 204-11.

176. Rampal S, Mahadeva S, Guallar E, et al. Ethnic differences in the prevalence of metabolic syndrome: results from a multi-ethnic population-based survey in Malaysia. *PLoS ONE [Electronic Resource]* 2012; **7**(9): e46365.

177. Saukkonen T, Jokelainen J, Timonen M, et al. Prevalence of metabolic syndrome components among the elderly using three different definitions: a cohort study in Finland. *Scandinavian Journal of Primary Health Care* 2012; **30**(1): 29-34.

178. Belfki H, Ben Ali S, Aounallah-Skhiri H, et al. Prevalence and determinants of the metabolic syndrome among Tunisian adults: results of the Transition and Health Impact in North Africa (TAHINA) project. *Public Health Nutrition* 2013; **16**(4): 582-90.

179. Del Brutto OH, Zambrano M, Penaherrera E, Montalvan M, Pow-Chon-Long F, Tettamanti D. Prevalence of the metabolic syndrome and its correlation with the cardiovascular health status in stroke- and ischemic heart disease-free Ecuadorian natives/mestizos aged >=40 years living in Atahualpa: a population-based study. *Diabetes & Metabolic Syndrome* 2013; **7**(4): 218-22.

180. Esmailzadehha N, Ziaee A, Kazemifar AM, Ghorbani A, Oveisi S. Prevalence of metabolic syndrome in Qazvin Metabolic Diseases Study (QMDS), Iran: a comparative analysis of six definitions. *Endocrine Regulations* 2013; **47**(3): 111-20.

181. Gundogan K, Bayram F, Gedik V, et al. Metabolic syndrome prevalence according to ATP III and IDF criteria and related factors in Turkish adults. *Archives of Medical Science* 2013; **9**(2): 243-53.

182. Kumbasar B, Yenigun M, Ataoglu HE, et al. The prevalence of metabolic syndrome in different ethnic groups in Turkey. *Journal of International Medical Research* 2013; **41**(1): 188-99.

183. Lakshmipriya N, Gayathri R, Praseena K, et al. Type of vegetable oils used in cooking and risk of metabolic syndrome among Asian Indians. *International Journal of Food Sciences & Nutrition* 2013; **64**(2): 131-9.

184. Latifi SM, Jalali MT, Shahbazian H, Amani R, Nikhoo A, Aleali AM. Metabolic syndrome and its correlated factors in an urban population in South West of Iran. *Journal of Diabetes and Metabolic Disorders* 2013; **12 (1) (no pagination)**(11).

185. Li J, Shi Y, Yan J, Xu W, Weng J. [The prevalence and risk factors of metabolic syndrome among adult residents in Guangdong and Jiangsu provinces in China]. [Chinese]. *Zhonghua nei ke za zhi* 2013; **52**(8): 659-63.

186. Li YQ, Zhao LQ, Liu XY, et al. Prevalence and distribution of metabolic syndrome in a southern Chinese population. Relation to exercise, smoking, and educational level. *Saudi Medical Journal* 2013; **34**(9): 929-36.

187. Lim ES, Ko YK, Ban KO. Prevalence and risk factors of metabolic syndrome in the Korean population--Korean National Health Insurance Corporation Survey 2008. *Journal of Advanced Nursing* 2013; **69**(7): 1549-61.

188. Lim S, Jang HC, Park KS, et al. Changes in metabolic syndrome in American and Korean youth, 1997-2008. *Pediatrics* 2013; **131**(1): e214-22.

189. Marcuello C, Calle-Pascual AL, Fuentes M, et al. Prevalence of the metabolic syndrome in Spain using regional cutoff points for waist circumference: the di@bet.es study. *Acta Diabetologica* 2013; **50**(4): 615-23.

190. Peer N, Steyn K, Lombard C, Gwebushe N, Levitt N. Prevalence of the metabolic syndrome in the urban black population of Cape Town. *Journal of Endocrinology, Metabolism and Diabetes of South Africa* 2013; **18 (1)**: 44.

191. Pessinaba S, Mbaye A, Yabeta GAD, et al. Prevalence survey of cardiovascular risk factors in the general population in St. Louis (Senegal). [French, English]. *Annales de Cardiologie et d'Angeiologie* 2013; **62**(4): 253-8.

192. Pimenta AM, Felisbino-Mendes MS, Velasquez-Melendez G. Clustering and combining pattern of metabolic syndrome components in a rural Brazilian adult population. *Sao Paulo Medical Journal = Revista Paulista de Medicina* 2013; **131**(4): 213-9.

193. Rodriguez F, Naderi S, Wang Y, Johnson CE, Foody JM. High prevalence of metabolic syndrome in young Hispanic women: findings from the national Sister to Sister campaign. *Metabolic Syndrome & Related Disorders* 2013; **11**(2): 81-6.

194. Schmitt AC, Cardoso MR, Lopes H, et al. Prevalence of metabolic syndrome and associated factors in women aged 35 to 65 years who were enrolled in a family health program in Brazil. *Menopause* 2013; **20**(4): 470-6.

195. Shalini M, Suresh Babu KP, Srinivasa Murthy AG, et al. Metabolic syndrome among urban and rural women population - A cross sectional study. *Journal of Clinical and Diagnostic Research* 2013; **7**(9): 1938-40.

196. Sherpa LY, Deji, Stigum H, Chongsuvivatwong V, Nafstad P, Bjertness E. Prevalence of metabolic syndrome and common metabolic components in high altitude farmers and herdsmen at 3700 m in Tibet. *High Altitude Medicine & Biology* 2013; **14**(1): 37-44.

197. Tsou MT, Chang BCC. Sex differences in metabolic syndrome of metropolitan elderly people in Northern Taiwan. *Journal of Clinical Gerontology and Geriatrics* 2013; **4**(2): 42-50.

198. Wang GR, Li L, Pan YH, et al. Prevalence of metabolic syndrome among urban community residents in China. *BMC Public Health* 2013; **13**: 599.

199. Xi B, He D, Hu Y, Zhou D. Prevalence of metabolic syndrome and its influencing factors among the Chinese adults: the China Health and Nutrition Survey in 2009. *Preventive Medicine* 2013; **57**(6): 867-71.

200. Zhang WH, Xue P, Yao MY, Chang HM, Wu Y, Zhang L. Prevalence of metabolic syndrome and its relationship with physical activity in suburban Beijing, China. *Annals of Nutrition & Metabolism* 2013; **63**(4): 298-304.

201. Al Mamun MA, Jesmin S, Rahman MA, et al. Metabolic syndrome prevalence and its components among rural Bangladeshi women. *Diabetes Research and Clinical Practice* 2014; **106**: S151.

202. Al-Daghri NM, Alkharfy KM, Al-Attas OS, et al. Gender-dependent associations between socioeconomic status and metabolic syndrome: a cross-sectional study in the adult Saudi population. *BMC Cardiovascular Disorders* 2014; **14**: 51.

203. Bermudez V, Rojas J, Salazar J, et al. The Maracaibo city metabolic syndrome prevalence study: Primary results and agreement level of 3 diagnostic criteria. *Revista Latinoamericana de Hipertension* 2014; **9**(4): 20-32.

204. Binh TQ, Phuong PT, Nhung BT, Tung do D. Metabolic syndrome among a middle-aged population in the Red River Delta region of Vietnam. *BMC Endocrine Disorders* 2014; **14**: 77.

205. Deedwania PC, Gupta R, Sharma KK, et al. High prevalence of metabolic syndrome among urban subjects in India: a multisite study. *Diabetes & Metabolic Syndrome* 2014; **8**(3): 156-61.

206. El Brini O, Akhouayri O, Gamal A, Mesfioui A, Benazzouz B. Prevalence of metabolic syndrome and its components based on a harmonious definition among adults in Morocco. *Diabetes, Metabolic Syndrome and Obesity: Targets and Therapy* 2014; **7**: 341-6.

207. Hu Z, Zhang R, Huang L, Zhou B, He D, Ding G. [A comparison of applicative effect with different diagnostic criteria of metabolic syndrome among the urban adult population]. *Chung-Hua Yu Fang i Hsueh Tsa Chih [Chinese Journal of Preventive Medicine]* 2014; **48**(6): 507-11.

208. Kang YU, Kim HY, Choi JS, et al. Metabolic syndrome and chronic kidney disease in an adult Korean population: results from the Korean National Health Screening. *PLoS ONE [Electronic Resource]* 2014; **9**(5): e93795.

209. Moreira GC, Cipullo JP, Ciorlia LA, Cesarino CB, Vilela-Martin JF. Prevalence of metabolic syndrome: association with risk factors and cardiovascular complications in an urban population. *PLoS ONE [Electronic Resource]* 2014; **9**(9): e105056.

210. Salas R, Bibiloni MDM, Ramos E, et al. Metabolic syndrome prevalence among Northern Mexican adult population. *PLoS ONE* 2014; **9 (8) (no pagination)**(e105581).

211. Sy RG, Llanes EJB, Reganit PFM, et al. Socio-demographic factors and the prevalence of metabolic syndrome among filipinos from the LIFECARE cohort. *Journal of Atherosclerosis and Thrombosis* 2014; **21**(SUPPL. 1): S9-S17.

212. Tsogzolbaatar E, Kotani K, Dambadarjaa D, Aoyama A, Tsuboi T, Ae A. Epidemiologic features of metabolic syndrome in a general Mongolian population. *Obesity Reviews* 2014; **15**: 68.

213. Xu S, Ming J, Yang C, et al. Urban, semi-urban and rural difference in the prevalence of metabolic syndrome in Shaanxi province, northwestern China: a population-based survey. *BMC Public Health* 2014; **14**: 104.

214. Yu S, Guo X, Yang H, Zheng L, Sun Y. An update on the prevalence of metabolic syndrome and its associated factors in rural northeast China. *BMC Public Health* 2014; **14**: 877.

215. Zhao Y, Yan H, Yang R, Li Q, Dang S, Wang Y. Prevalence and determinants of metabolic syndrome among adults in a rural area of Northwest China. *PLoS ONE [Electronic Resource]* 2014; **9**(3): e91578.

216. Amarasinghe S, Balakumar S, Arasaratnam V. Prevalence and factors associated with metabolic syndrome among Tamils aged over 18 years in Jaffna district, Sri Lanka. *Journal of Diabetes and Metabolic Disorders* 2015; **14 (1) (no pagination)**(61).

217. Karimi F, Jahandideh D, Dabbaghmanesh M, Fattahi M, Omrani GR. The prevalence of metabolic syndrome and its components among adults in a rural community, Fars, Iran. *International Cardiovascular Research Journal* 2015; **9**(2): 94-9.

218. Khan RJ, Gebreab SY, Sims M, Riestra P, Xu R, Davis SK. Prevalence, associated factors and heritabilities of metabolic syndrome and its individual components in African Americans: the Jackson Heart Study. *BMJ Open* 2015; **5**(10): e008675.

219. Kosa Z, Moravcsik-Kornyicki A, Dioszegi J, et al. Prevalence of metabolic syndrome among Roma: a comparative health examination survey in Hungary. *European Journal of Public Health* 2015; **25**(2): 299-304.

220. Li D, Liu J, Zhang Q, et al. The prevalence of metabolic syndrome prevalence and its risk factors among middle-aged and elderly Chinese in Gansu Province, western China. *Diabetes/Metabolism Research and Reviews* 2015; **31**: 27-8.

221. Murguia-Romero M, Jimenez-Flores JR, Sigrist-Flores SC, et al. Prevalence Of Metabolic Syndrome In Young Mexicans: A Sensitivity Analysis on Its Components. *Nutricion Hospitalaria* 2015; **32**(1): 189-95.

222. Obeidat AA, Ahmad MN, Haddad FH, Azzeh FS. Alarming high prevalence of metabolic syndrome among jordanian adults. *Pakistan Journal of Medical Sciences* 2015; **31**(6): 1377-82.

223. Park E, Kim J. Gender- and age-specific prevalence of metabolic syndrome among Korean adults: analysis of the fifth Korean National Health and Nutrition Examination Survey. *Journal of Cardiovascular Nursing* 2015; **30**(3): 256-66.

224. Park S, Kim SJ, Lee M, Kang KA, Hendrix E. Prevalence and associated factors of metabolic syndrome among South Korean adults. *Journal of Community Health Nursing* 2015; **32**(1): 24-38.

225. Peer N, Lombard C, Steyn K, Levitt N. High prevalence of metabolic syndrome in the Black population of Cape Town: The Cardiovascular Risk in Black South Africans (CRIBSA) study. *European Journal of Preventive Cardiology* 2015; **22**(8): 1036-42.

226. Song QB, Zhao Y, Liu YQ, Zhang J, Xin SJ, Dong GH. Sex difference in the prevalence of metabolic syndrome and cardiovascular-related risk factors in urban adults from 33 communities of China: The CHPSNE study. *Diabetes & Vascular Disease Research* 2015; **12**(3): 189-98.

227. Strand MA, Will T, Gu X, Perry J. A descriptive study of the progression of the Metabolic Syndrome in middle-aged Chinese population. *International Quarterly of Community Health Education* 2015; **35**(2): 163-76.

228. Tao R, Wu M, Qin Y, et al. Epidemiological characteristics of metabolic syndrome and comparison between its different diagnostic criteria in adults of Jiangsu Province. [Chinese]. *Journal of Jilin University Medicine Edition* 2015; **41**(1): 181-9.

229. Al-Thani MH, Al-Thani AAM, Cheema S, et al. Prevalence and determinants of metabolic syndrome in Qatar: Results from a National Health Survey. *BMJ Open* 2016; **6 (9) (no pagination)**(e009514).

230. Dhaheri ASA, Mohamad MN, Jarrar AH, et al. A cross-sectional study of the prevalence of metabolic syndrome among young female Emirati adults. *PLoS ONE* 2016; **11 (7) (no pagination)**(e0159378).

231. Ding Z, Pi F, Zhang S, et al. Establishment and application of a new diagnostic definition of metabolic syndrome in the Shantou region of southern China. *Scientific Reports* 2016; **6**: 22210.

232. Franca SL, Lima SS, Vieira JR. Metabolic Syndrome and Associated Factors in Adults of the Amazon Region. *PLoS ONE [Electronic Resource]* 2016; **11**(12): e0167320.

233. Guo H, Liu J, Zhang J, et al. The Prevalence of Metabolic Syndrome Using Three Different Diagnostic Criteria among Low Earning Nomadic Kazakhs in the Far Northwest of China: New Cut-Off Points of Waist Circumference to Diagnose MetS and Its Implications. *PLoS ONE [Electronic Resource]* 2016; **11**(2): e0148976.

234. Houti L, Hamani-Medjaoui I, Lardjam-Hetraf SA, et al. Prevalence of metabolic syndrome and its related risk factors in the city of Oran, Algeria: The ISOR study. *Ethnicity and Disease* 2016; **26**(1): 99-106.

235. Jiang B, Li B, Wang Y, et al. The nine-year changes of the incidence and characteristics of metabolic syndrome in China: longitudinal comparisons of the two cross-sectional surveys in a newly formed urban community. *Cardiovascular Diabetology* 2016; **15**: 84.

236. Khan SA, Jackson RT. The prevalence of metabolic syndrome among low-income South Asian Americans. *Public Health Nutrition* 2016; **19**(3): 418-28.

237. Kim S, So WY. Prevalence and correlates of metabolic syndrome and its components in elderly Korean adults. *Experimental Gerontology* 2016; **84**: 107-12.

238. Krishnadath ISK, Toelsie JR, Hofman A, Jaddoe VWV. Ethnic disparities in the prevalence of metabolic syndrome and its risk factors in the Suriname Health Study: A cross-sectional population study. *BMJ Open* 2016; **6 (12) (no pagination)**(943162).

239. Lee S, Ko Y, Kwak C, Yim ES. Gender differences in metabolic syndrome components among the Korean 66-year-old population with metabolic syndrome.[Erratum appears in BMC Geriatr. 2016;16:71; PMID: 27013318]. *BMC Geriatrics* 2016; **16**: 27.

240. Liu Y, Huang J, Xu G, et al. Prevalence and Determinants of Metabolic Syndrome-identified by Three Criteria among Men in Rural China: A Population-based Cross-sectional Study Conducted during 2007-2008. *Journal of Nutrition, Health & Aging* 2016; **20**(5): 574-82.

241. Martinez-Larrad MT, Corbaton-Anchuelo A, Fernandez-Perez C, Lazcano-Redondo Y, Escobar-Jimenez F, Serrano-Rios M. Metabolic syndrome, glucose tolerance categories and the cardiovascular risk in Spanish population. *Diabetes Research & Clinical Practice* 2016; **114**: 23-31.

242. Miguel-Soca PE, Rivas-Estevez M, Sarmiento-Teruel Y, et al. Prevalence of metabolic syndrome risk factors in adults in holguin, Cuba (2004-2013). *MEDICC Review* 2016; **18**(1-2): 28-33.

243. Parini A, Ivkovic V, Vrdoljak A, et al. Metabolic syndrome in european rural population-data from the brisghella heart study (Italy) and ENAH study (Croatia). *Journal of Hypertension* 2016; **34**: e87.

244. Soysal A, Simsek H, Doganay S, Gunay T. Prevalence of metabolic syndrome and affecting factors among individuals aged 30 and over in Balcova district of Izmir. *Balkan Medical Journal* 2016; **33**(3): 331-8.

245. Suliga E, Koziel D, Gluszek S. Prevalence of metabolic syndrome in normal weight individuals. *Annals of Agricultural & Environmental Medicine* 2016; **23**(4): 631-5.

246. Voevoda MI, Koval'kova NA, Ragino YI, Travnikova NY, Denisova DV. [Prevalence of metabolic syndrome in 25-45-year-old Novosibirsk dwellers]. *Terapevticheskii Arkhiv* 2016; **88**(10): 51-6.

247. Barros BSV, Santos DC, Pizarro MH, de Melo LGN, Gomes MB. Type 1 diabetes and non-alcoholic fatty liver disease: When should we be concerned? a nationwide study in Brazil. *Nutrients* 2017; **9 (8) (no pagination)**(878).

248. de Mello Fontanelli M, Sales CH, Carioca AAF, Marchioni DM, Fisberg RM. The relationship between carbohydrate quality and the prevalence of metabolic syndrome: challenges of glycemic index and glycemic load. *European Journal of Nutrition* 2017: 1-9.

249. Goetzel RZ, Kent K, Henke RM, et al. Prevalence of Metabolic Syndrome in an Employed Population as Determined by Analysis of Three Data Sources. *Journal of Occupational and Environmental Medicine* 2017; **59**(2): 161-8.

250. Hosseini N, Talaei M, Dianatkhah M, Sadeghi M, Oveisgharan S, Sarrafzadegan N. Determinants of Incident Metabolic Syndrome in a Middle Eastern Population: Isfahan Cohort Study. *Metabolic Syndrome and Related Disorders* 2017; **15**(7): 354-62.

251. Lu J, Li M, Xu Y, et al. Metabolic syndrome among adults in China: The 2010 China Noncommunicable Disease Surveillance. *Journal of Clinical Endocrinology and Metabolism* 2017; **102**(2): 507-15.

252. Moore JX, Chaudhary N, Akinyemiju T. Metabolic Syndrome Prevalence by Race/Ethnicity and Sex in the United States, National Health and Nutrition Examination Survey, 1988-2012. *Preventing Chronic Disease* 2017; **14**: E24.

253. Noshad S, Abbasi M, Etemad K, et al. Prevalence of metabolic syndrome in Iran: A 2011 update. *Journal Of Diabetes* 2017; **9**(5): 518-25.

254. Orces C. Prevalence of metabolic syndrome among older adults in Ecuador. *Journal of the American Geriatrics Society* 2017; **65**: S49.

255. Ortiz-Rodriguez MA, Yanez-Velasco L, Carnevale A, et al. Prevalence of metabolic syndrome among elderly Mexicans. *Archives of Gerontology and Geriatrics* 2017; **73**: 288-93.

256. Owolabi EO, Goon DT, Adeniyi OV, Adedokun AO, Seekoe E. Prevalence and correlates of metabolic syndrome among adults attending healthcare facilities in eastern cape, South Africa. *Open Public Health Journal* 2017; **10**: 148-59.

257. Raposo L, Severo M, Barros H, Santos AC. The prevalence of the metabolic syndrome in Portugal: the PORMETS study. *BMC public health* 2017; **17**(1): 555.

258. Slagter SN, Van Waateringe RP, Van Beek AP, van der Klauw MM, Wolffenbuttel BHR, van Vliet-Ostaptchouk JV. Sex, BMI and age differences in metabolic syndrome: The dutch lifelines cohort study. *Endocrine Connections* 2017; **6**(4): 278-88.

259. Tran BT, Jeong BY, Oh JK. The prevalence trend of metabolic syndrome and its components and risk factors in Korean adults: results from the Korean National Health and Nutrition Examination Survey 2008-2013. *BMC public health* 2017; **17**(1): 71.

| **Supplementary Table 6. Quality assessment of selected articles** | | | | |
| --- | --- | --- | --- | --- |
| **First author** | **Publication year** | **Is the patient definition adequate?*** | **Representativeness of the population**** |  |
| Ford | 2002 | a | a |  |
| Al-Lawati | 2003 | a | a |  |
| Azizi | 2003 | a | a |  |
| Ford | 2003 | a | a |  |
| Park | 2003 | a | a |  |
| Tanchoco | 2003 | a | a |  |
| Chuang | 2004 | a | a |  |
| Farrell | 2004 | a | a |  |
| Ford | 2004 | a | a |  |
| Gupta | 2004 | b | a |  |
| Jia | 2004 | a | a |  |
| Jorgensen | 2004 | a | b |  |
| Kim | 2004 | a | a |  |
| Oh | 2004 | a | b |  |
| Parikka | 2004 | a | a |  |
| Santos | 2004 | b | a |  |
| Al-Nozha | 2005 | a | a |  |
| Athyros | 2005 | a | a |  |
| Bo | 2005 | b | a |  |
| Boronat | 2005 | b | a |  |
| Choi | 2005 | a | a |  |
| Dekker | 2005 | a | a |  |
| Florez | 2005 | a | a |  |
| Grandinetti | 2005 | a | a |  |
| Ko | 2005 | a | a |  |
| Li | 2005 | a | a |  |
| Miccoli | 2005 | a | a |  |
| Romero | 2005 | b | b |  |
| Rosenbaum | 2005 | b | a |  |
| Son le | 2005 | a | b |  |
| Tanaka | 2005 | a | a |  |
| Thomas | 2005 | a | a |  |
| Tillin | 2005 | a | a |  |
| Urashima | 2005 | a | a |  |
| Yan | 2005 | c | a |  |
| Zhang | 2005 | b | b |  |
| Arai | 2006 | a | a |  |
| Bouguerra | 2006 | a | a |  |
| Fakhrzadeh | 2006 | b | a |  |
| Feng | 2006 | a | a |  |
| Harzallah | 2006 | a | b |  |
| He | 2006 | a | a |  |
| Hu | 2006 | b | a |  |
| Ko | 2006 | a | a |  |
| Lawati | 2006 | a | a |  |
| Liu | 2006 | a | a |  |
| Liu | 2006 | a | a |  |
| Lu | 2006 | a | a |  |
| Miyatake | 2006 | b | a |  |
| Nilsson | 2006 | a | b |  |
| PARK | 2006 | a | a |  |
| Patel | 2006 | a | a |  |
| Qahtani | 2006 | a | a |  |
| Ravaglia | 2006 | a | a |  |
| Seclen | 2006 | a | b |  |
| Szurkowska | 2006 | a | a |  |
| DECODA | 2007 | a | a |  |
| Deepa | 2007 | a | a |  |
| Hildrum | 2007 | a | a |  |
| Hwang | 2007 | a | a |  |
| Khader | 2007 | a | a |  |
| Kim | 2007 | a | a |  |
| Kozan | 2007 | b | a |  |
| Li | 2007 | a | a |  |
| Lin | 2007 | a | a |  |
| Mattsson | 2007 | a | a |  |
| Park | 2007 | a | a |  |
| Prabhakaran | 2007 | a | a |  |
| Sandhofer | 2007 | b | a |  |
| Santos | 2007 | b | a |  |
| Yang | 2007 | a | a |  |
| Yoon | 2007 | a | a |  |
| Zabetian | 2007 | b | b |  |
| Bindraban | 2008 | a | a |  |
| Erem | 2008 | a | a |  |
| Fiuza | 2008 | a | a |  |
| Hu | 2008 | a | a |  |
| Kelliny | 2008 | a | a |  |
| Maggi | 2008 | a | a |  |
| Malik | 2008 | a | a |  |
| Marquezine | 2008 | b | a |  |
| Moebus | 2008 | a | a |  |
| Mokan | 2008 | b | a |  |
| Neuhauser | 2008 | a | a |  |
| Park | 2008 | a | a |  |
| Park | 2008 | a | a |  |
| Perez | 2008 | a | b |  |
| Ramos | 2008 | a | a |  |
| Suarez | 2008 | a | b |  |
| Sun | 2008 | a | a |  |
| Surana | 2008 | a | b |  |
| Welin | 2008 | a | a |  |
| Arikan | 2009 | a | a |  |
| Bener | 2009 | a | a |  |
| Buckland | 2009 | a | b |  |
| Can | 2009 | a | a |  |
| Delavar | 2009 | b | b |  |
| Donk | 2009 | a | a |  |
| Ekelund | 2009 | a | a |  |
| Elasmi | 2009 | a | a |  |
| Ervin | 2009 | a | a |  |
| Fiuza | 2009 | a | a |  |
| Gelaye | 2009 | b | a |  |
| Gundogan | 2009 | a | b |  |
| Hadaegh | 2009 | a | b |  |
| Quintana | 2009 | a | a |  |
| Saucedo | 2009 | b | b |  |
| Sharifi | 2009 | a | a |  |
| Tao | 2009 | a | a |  |
| Xu | 2009 | a | a |  |
| Zuo | 2009 | a | a |  |
| Al-Daghri | 2010 | b | a |  |
| Allal-Elasmi | 2010 | a | a |  |
| Bhat | 2010 | b | b |  |
| Biad | 2010 | a | a |  |
| Ferguson | 2010 | a | b |  |
| Ferguson | 2010 | a | a |  |
| Flowers | 2010 | a | a |  |
| Guo | 2010 | a | a |  |
| Khunti | 2010 | b | a |  |
| Li | 2010 | a | a |  |
| Li | 2010 | a | a |  |
| Misra | 2010 | b | b |  |
| Moebus | 2010 | a | a |  |
| Oguz | 2010 | a | a |  |
| Oladapo | 2010 | a | a |  |
| Ravikiran | 2010 | b | a |  |
| Schipf | 2010 | b | a |  |
| Sidorenkov | 2010 | a | a |  |
| Valenzuela | 2010 | a | a |  |
| Wang | 2010 | b | a |  |
| Xavier | 2010 | a | b |  |
| Xu | 2010 | a | a |  |
| Zhao | 2010 | a | b |  |
| Zhao | 2010 | b | a |  |
| Aekplakorn | 2011 | a | a |  |
| Alkerwi | 2011 | a | a |  |
| Alvi | 2011 | a | b |  |
| Burazor | 2011 | b | a |  |
| Gavrila | 2011 | a | a |  |
| Janszky | 2011 | a | a |  |
| Jiang | 2011 | b | a |  |
| Kawada | 2011 | a | a |  |
| Li | 2011 | b | a |  |
| Lim | 2011 | a | a |  |
| Mohamud | 2011 | a | a |  |
| Motala | 2011 | b | b |  |
| Oliveira | 2011 | a | b |  |
| Orhan | 2011 | a | b |  |
| Pan | 2011 | a | a |  |
| Pimenta | 2011 | a | b |  |
| Riediger | 2011 | a | a |  |
| Sawant | 2011 | b | b |  |
| Sharma | 2011 | b | a |  |
| Tsai | 2011 | a | b |  |
| Wei | 2011 | a | a |  |
| Wen | 2011 | a | a |  |
| Zhao | 2011 | a | a |  |
| Al Zenki | 2012 | a | b |  |
| Alzahrani | 2012 | a | b |  |
| Belfki | 2012 | a | a |  |
| Cai | 2012 | a | a |  |
| Cetin | 2012 | a | b |  |
| Deka | 2012 | a | a |  |
| Fei | 2012 | a | a |  |
| Gundogan | 2012 | a | a |  |
| Ilow | 2012 | a | a |  |
| Jesmin | 2012 | b | a |  |
| Kaduka | 2012 | a | b |  |
| Kim | 2012 | a | a |  |
| Lao | 2012 | a | a |  |
| Li | 2012 | a | a |  |
| Mahjoub | 2012 | a | b |  |
| Metelskaya | 2012 | a | a |  |
| Mora Garcia | 2012 | a | b |  |
| Nezhad | 2012 | a | a |  |
| Prasad | 2012 | a | a |  |
| Rampal | 2012 | a | a |  |
| Saukkonen | 2012 | a | b |  |
| Belfki | 2013 | a | a |  |
| Brutto | 2013 | b | b |  |
| Esmailzadehha | 2013 | a | a |  |
| Kumbasar | 2013 | a | a |  |
| Lakshmipriya | 2013 | a | a |  |
| Li | 2013 | a | a |  |
| Li | 2013 | a | a |  |
| Lim | 2013 | a | a |  |
| Marcuello | 2013 | a | a |  |
| Peer | 2013 | a | a |  |
| Pessinaba | 2013 | a | a |  |
| Pimenta | 2013 | a | b |  |
| Rodriguez | 2013 | a | a |  |
| Schmitt | 2013 | a | b |  |
| Shahbazian | 2013 | b | b |  |
| Shalini | 2013 | b | a |  |
| Sherpa | 2013 | a | b |  |
| Tsou | 2013 | a | a |  |
| Wang | 2013 | a | a |  |
| Xi | 2013 | a | a |  |
| Zhang | 2013 | a | a |  |
| Al-Thani | 2014 | a | a |  |
| Bermúdez | 2014 | a | a |  |
| Binh | 2014 | a | a |  |
| Brini | 2014 | a | b |  |
| Daghri | 2014 | a | a |  |
| Deedwania | 2014 | a | a |  |
| Hu | 2014 | a | a |  |
| Kang | 2014 | a | a |  |
| Mamun | 2014 | b | a |  |
| Moreira | 2014 | a | a |  |
| RG. Sy | 2014 | b | a |  |
| Salas | 2014 | a | a |  |
| T Enkh-Oyun | 2014 | b | a |  |
| Xu | 2014 | a | a |  |
| Yu | 2014 | a | a |  |
| Zhao | 2014 | a | a |  |
| Amarasinghe | 2015 | b | b |  |
| Karimi | 2015 | a | a |  |
| Khan | 2015 | a | a |  |
| Khan | 2015 | a | a |  |
| Kosa | 2015 | a | b |  |
| Li | 2015 | b | a |  |
| Obeidat | 2015 | b | b |  |
| Park | 2015 | a | a |  |
| Park | 2015 | a | a |  |
| Peer | 2015 | a | a |  |
| Romero | 2015 | a | a |  |
| Song | 2015 | a | a |  |
| Strand | 2015 | a | b |  |
| Tao | 2015 | a | a |  |
| Dhaheri | 2016 | a | b |  |
| Ding | 2016 | a | a |  |
| Franca | 2016 | a | b |  |
| Guo | 2016 | a | a |  |
| Houti | 2016 | a | b |  |
| Jiang | 2016 | a | a |  |
| Kim | 2016 | a | a |  |
| Krishnadath | 2016 | a | a |  |
| Larrad | 2016 | a | a |  |
| Lee | 2016 | a | a |  |
| Liu | 2016 | a | a |  |
| Parini | 2016 | b | a |  |
| Soca | 2016 | a | a |  |
| Soysal | 2016 | a | a |  |
| Suliga | 2016 | a | a |  |
| Voevoda | 2016 | a | b |  |
| Barros | 2017 | a | a |  |
| Fontanelli | 2017 | a | b |  |
| Goetzel | 2017 | a | a |  |
| Hosseini | 2017 | a | a |  |
| Lu | 2017 | a | a |  |
| Moore | 2017 | a | a |  |
| Noshad | 2017 | a | a |  |
| Orces | 2017 | a | a |  |
| Owolabi | 2017 | b | b |  |
| Raposo | 2017 | a | a |  |
| Rodriguez | 2017 | a | b |  |
| Slagter | 2017 | a | a |  |
| Tran | 2017 | a | a |  |
| According to Newcastle-Ottawa quality assessment scale: *a=yes, with independent validation; b=yes, e.g. record linkage or based on self reports; c=no description **a=consecutive or obviously representative series of population; b=potential for selection biases or not stated | | | | |
|  |  |  |  |  |
|  |  |  |  |  |

| **Supplementary Table 7 Results of Univariate and Multivariate Meta-regression Analysis** | | | | | | | |
| --- | --- | --- | --- | --- | --- | --- | --- |
| Variable | **Univariate Regression Analysis** | | | | Multivariate Regression Analysis | |  |
|  | No. of Studies (%) | Crude RR | 95% CI | *p* | Adjusted RR | 95% CI | *p* |
| **Age** |  |  |  |  |  |  |  |
| <55 | 122 (42.36%) | 1 (Reference) | 1 (Reference) |  | 1 (Reference) | 1 (Reference) |  |
| ≥55 | 152 (52.78%) | 1.19 | (1.05-1.36) | 0.007 | 1.23 | (1.09-1.38) | 0.001 |
| NM | 14 (4.86%) | 0.80 | (0.59-1.07) | 0.128 | 0.93 | (0.70-1.23) | 0.606 |
| **Region** |  |  |  |  |  |  |  |
| Africa | 16 (5.56%) | 1 (Reference) | 1 (Reference) |  | 1 (Reference) | 1 (Reference) |  |
| Asia | 163 (56.60%) | 0.75 | (0.57 -0.99) | 0.042 | 0.79 | (0.61-1.02 | 0.071 |
| Europe | 52 (18.06%) | 1.02 | (0.76-1.38) | 0.883 | 1.08 | (0.81-1.43) | 0.609 |
| America | 57 (19.97%) | 0.94 | (0.70-1.26) | 0.681 | 0.96 | (0.73-1.27) | 0.779 |
| **Place** |  |  |  |  |  |  |  |
| Rural | 21 (7.29%) | 1 (Reference) | 1 (Reference) |  | 1 (Reference) | 1 (Reference) |  |
| Urban | 51 (17.71%) | 1.65 | (1.26-2.16) | <0.001 | 1.70 | (1.31-2.19) | <0.001 |
| Mixed | 74 (25.69%) | 1.56 | (1.20-2.03) | 0.001 | 1.67 | (1.30-2.14) | <0.001 |
| NM | 142 (49.31%) | 1.43 | (1.12-1.83) | 0.004 | 1.48 | (1.16-1.88) | 0.002 |
| **Period** |  |  |  |  |  |  |  |
| 1980-2004 | 112 (38.89%) | 1 (Reference) | 1 (Reference) |  | 1 (Reference) | 1 (Reference) |  |
| 2005-2014 | 114 (39.58%) | 1.33 | (1.16-1.53) | <0.001 | 1.34 | (1.17-1.53) | <0.001 |
| NM | 62 (21.53%) | 1.22 | (1.04-1.44) | 0.016 | 1.24 | (1.03-1.49) | 0.025 |
| **Criteria** |  |  |  |  |  |  |  |
| ATP-III | 95 (32.99%) | 1 (Reference) | 1 (Reference) |  | 1 (Reference) | 1 (Reference) |  |
| IDF | 41 (14.24%) | 1.14 | (0.93-1.39) | 0.194 | 1.17 | (0.97-1.41) | 0.099 |
| JIS | 4 (1.39%) | 1.11 | (0.64-1.91) | 0.711 | 1.05 | (0.64-1.74) | 0.849 |
| NMLBI | 10 (3.27%) | 1.30 | (0.91-1.85) | 0.150 | 1.25 | (0.89-1.76) | 0.192 |
| Mixed | 138 (47.92%) | 0.98 | (0.85-1.13) | 0.817 | 1.03 | (0.90-1.17) | 0.691 |
| **Measurement method** |  |  |  |  |  |  |  |
| Trained staff | 273 (94.79%) | 1 (Reference) | 1 (Reference) |  |  |  |  |
| NM | 15 (5.21%) | 1.15 | (0.87-1.53) | 0.326 | 1.06 | (0.82-1.38) | 0.657 |
| **Study quality** |  |  |  |  |  |  |  |
| Low | 85 (29.62%) | 1 (Reference) | 1 (Reference) |  |  |  |  |
| High | 202 (70.36%) | 0.87 | (0.76-0.99) | 0.042 | 0.93 | (0.80-1.08) | 0.330 |
| ATP-III, Adult Treatment Panel III; IDF, International Diabetes Federation; NHLBI, National Heart, Lung, and Blood Institute; NM, not mentioned. | | | | | | | |

**Supplementary Table 8 Results of sensitivity analysis**

| **Study omitted** | **Estimate** | **[95% Conf. Interval]** | |
| --- | --- | --- | --- |
| Kang ((2014)) | 0.4295111 | 0.4078232 | 0.45119902 |
| ES Lim ((2013)) | 0.42940822 | 0.41176939 | 0.44704705 |
| Wen ((2011)) | 0.42964444 | 0.41426212 | 0.44502673 |
| Lee (2016) | 0.4277505 | 0.41234109 | 0.44315991 |
| Lu (2017) | 0.42883855 | 0.41266751 | 0.44500956 |
| Slagter (2017) | 0.42757943 | 0.41219306 | 0.44296578 |
| Patel (2006) | 0.4286727 | 0.41260251 | 0.44474289 |
| Li (2007) | 0.42802534 | 0.41222769 | 0.44382298 |
| Moore (2017) | 0.42839965 | 0.41242391 | 0.44437543 |
| Szurkowska (2006) | 0.42897296 | 0.41292614 | 0.44501981 |
| Park (2008) | 0.42931205 | 0.41329569 | 0.44532841 |
| Moebus (2008) | 0.42854202 | 0.41254598 | 0.44453803 |
| Moebus (2010) | 0.42854142 | 0.4125455 | 0.44453734 |
| Tran (2017) | 0.42883283 | 0.41280583 | 0.44485983 |
| Lim (2011) | 0.42857116 | 0.41257432 | 0.44456801 |
| Liu (2006) | 0.42898977 | 0.41296861 | 0.4450109 |
| Sun (2008) | 0.42908549 | 0.41307119 | 0.4450998 |
| Chuang (2004) | 0.42975613 | 0.41383687 | 0.4456754 |
| Urashima (2005) | 0.42926639 | 0.41327474 | 0.44525808 |
| Wang (2013) | 0.42806286 | 0.41216433 | 0.44396138 |
| Li (2015) | 0.42731312 | 0.41163087 | 0.44299537 |
| Aekplakorn (2011) | 0.42881319 | 0.41282201 | 0.44480437 |
| Zhang (2013) | 0.42833188 | 0.41238225 | 0.44428152 |
| Feng (2006) | 0.42942306 | 0.4134523 | 0.4453938 |
| Ilow (2012) | 0.42810193 | 0.41218802 | 0.44401583 |
| Rampal (2012) | 0.42864323 | 0.41266477 | 0.44462171 |
| Al-Nozha (2005) | 0.42847839 | 0.41251329 | 0.44444349 |
| Fiuza (2008) | 0.4282594 | 0.41231918 | 0.44419962 |
| Fiuza (2009) | 0.42825869 | 0.41231838 | 0.44419897 |
| Li (2010) | 0.4288978 | 0.41291115 | 0.44488445 |
| Song (2015) | 0.42844108 | 0.41248077 | 0.44440138 |
| Yang (2007) | 0.42886046 | 0.41287741 | 0.44484347 |
| Sharma (2011) | 0.42920044 | 0.41322246 | 0.44517842 |
| Liu (2006) | 0.42951348 | 0.41355568 | 0.44547129 |
| DECODA (2007) | 0.42933607 | 0.41336513 | 0.44530702 |
| Cai (2012) | 0.42859578 | 0.41262648 | 0.44456509 |
| Park (2007) | 0.42974889 | 0.41381609 | 0.44568172 |
| Karimi (2015) | 0.42865387 | 0.41268185 | 0.44462585 |
| Park (2003) | 0.42839506 | 0.41244042 | 0.44434971 |
| Soysal (2016) | 0.42815128 | 0.41222125 | 0.44408128 |
| Suliga (2016) | 0.42704695 | 0.41132948 | 0.44276446 |
| Miyatake (2006) | 0.42941514 | 0.4134514 | 0.44537884 |
| Donk (2009) | 0.42875773 | 0.4127847 | 0.44473076 |
| Yu (2014) | 0.42840448 | 0.41245005 | 0.44435889 |
| Zabetian (2007) | 0.42842036 | 0.41246557 | 0.44437513 |
| Hildrum (2007) | 0.42875576 | 0.41278633 | 0.44472519 |
| Goetzel (2017) | 0.42660409 | 0.4109427 | 0.44226551 |
| Azizi (2003) | 0.42891622 | 0.41294518 | 0.44488725 |
| Daghri (2014) | 0.42833239 | 0.41238385 | 0.44428092 |
| Li (2011) | 0.42971647 | 0.41377637 | 0.44565657 |
| Ford (2002) | 0.42857248 | 0.41261163 | 0.44453329 |
| Noshad (2017) | 0.4280726 | 0.41214371 | 0.4440015 |
| Ford (2003) | 0.42798987 | 0.41206822 | 0.44391152 |
| Tao (2015) | 0.42808089 | 0.41215053 | 0.44401121 |
| Ford (2004) | 0.42853746 | 0.412579 | 0.44449589 |
| Yoon (2007) | 0.42882374 | 0.41285807 | 0.44478941 |
| Xi (2013) | 0.42833766 | 0.4123891 | 0.44428626 |
| Farrell (2004) | 0.42975667 | 0.41381779 | 0.44569555 |
| Liu (2016) | 0.42950675 | 0.41355428 | 0.44545925 |
| Tanaka (2005) | 0.42854553 | 0.41258836 | 0.44450268 |
| Rodriguez (2013) | 0.4278703 | 0.41195306 | 0.44378752 |
| PARK (2006) | 0.42926615 | 0.41330582 | 0.44522646 |
| Neuhauser (2008) | 0.42881051 | 0.41284797 | 0.44477302 |
| Li (2013) | 0.42846781 | 0.41251385 | 0.44442174 |
| Ervin (2009) | 0.42797866 | 0.41205198 | 0.44390538 |
| Deedwania (2014) | 0.42829812 | 0.41235161 | 0.44424465 |
| Kim (2004) | 0.42861518 | 0.41265732 | 0.44457301 |
| Romero (2015) | 0.42843282 | 0.41248068 | 0.44438493 |
| Hu (2006) | 0.42879787 | 0.4128373 | 0.44475847 |
| Hwang (2007) | 0.42891288 | 0.41295144 | 0.44487429 |
| Lao (2012) | 0.4294557 | 0.4135024 | 0.445409 |
| Park (2015) | 0.42874116 | 0.4127816 | 0.44470072 |
| Xu (2010) | 0.42873418 | 0.41277528 | 0.44469306 |
| Ramos (2008) | 0.42860419 | 0.41264784 | 0.44456053 |
| Zhao (2011) | 0.42798078 | 0.41205019 | 0.44391137 |
| Khan (2015) | 0.42760104 | 0.41169658 | 0.44350553 |
| Surana (2008) | 0.42827371 | 0.41232806 | 0.44421938 |
| Erem (2008) | 0.4284808 | 0.4125284 | 0.44443324 |
| Marcuello (2013) | 0.42795351 | 0.41202253 | 0.44388452 |
| Tillin (2005) | 0.4282718 | 0.41232613 | 0.44421747 |
| Lim (2013) | 0.42969087 | 0.41374671 | 0.44563505 |
| Belfki (2013) | 0.42820901 | 0.41226575 | 0.44415227 |
| Li (2013) | 0.42897791 | 0.41301945 | 0.44493636 |
| Ding (2016) | 0.42923269 | 0.41327628 | 0.44518906 |
| Tanchoco (2003) | 0.42948964 | 0.41353872 | 0.44544053 |
| Maggi (2008) | 0.42788759 | 0.41195935 | 0.4438158 |
| Kim (2007) | 0.42892992 | 0.41297194 | 0.44488788 |
| Mohamud (2011) | 0.42782319 | 0.41189787 | 0.44374847 |
| Gundogan (2013) | 0.4283911 | 0.41244158 | 0.44434059 |
| Kozan (2007) | 0.42866495 | 0.41270965 | 0.44462028 |
| Schipf (2010) | 0.42814198 | 0.41220096 | 0.44408301 |
| Athyros (2005) | 0.42784768 | 0.41192016 | 0.44377521 |
| Tao (2009) | 0.42941463 | 0.4134624 | 0.44536689 |
| Malik (2008) | 0.42780724 | 0.41188163 | 0.44373286 |
| Quintana (2009) | 0.42749745 | 0.41159049 | 0.44340441 |
| Prabhakaran (2007) | 0.42956764 | 0.41361931 | 0.44551596 |
| Wei (2011) | 0.42736733 | 0.41146922 | 0.44326544 |
| Raposo (2017) | 0.42807889 | 0.41214001 | 0.4440178 |
| Kim (2016) | 0.42856985 | 0.4126167 | 0.44452301 |
| Zuo (2009) | 0.42878374 | 0.41282782 | 0.44473967 |
| Guo (2016) | 0.42822713 | 0.41228271 | 0.44417155 |
| Janszky (2011) | 0.42712331 | 0.41124192 | 0.44300473 |
| Larrad (2016) | 0.42842659 | 0.41247651 | 0.44437668 |
| Wang (2010) | 0.42866999 | 0.41271555 | 0.44462442 |
| Metelskaya (2012) | 0.42839873 | 0.41244951 | 0.44434795 |
| Sidorenkov (2010) | 0.42931661 | 0.41336352 | 0.4452697 |
| Fei (2012) | 0.42868665 | 0.41273257 | 0.44464073 |
| Hu (2008) | 0.42809469 | 0.41215432 | 0.44403502 |
| Lu (2006) | 0.42865428 | 0.41270068 | 0.44460791 |
| Li (2012) | 0.42833731 | 0.41238964 | 0.44428498 |
| Bouguerra (2006) | 0.42911229 | 0.41315734 | 0.44506726 |
| Hu (2014) | 0.42841807 | 0.41246855 | 0.44436759 |
| Arai (2006) | 0.42881769 | 0.41286311 | 0.44477227 |
| Parikka (2004) | 0.42813304 | 0.41219097 | 0.44407511 |
| Kawada (2011) | 0.42638451 | 0.41055071 | 0.4422183 |
| Florez (2005) | 0.4284001 | 0.41245121 | 0.44434902 |
| Khunti (2010) | 0.42772648 | 0.41179934 | 0.44365364 |
| RG. Sy (2014) | 0.42873397 | 0.4127804 | 0.44468755 |
| Zhao (2014) | 0.42954555 | 0.41359735 | 0.44549379 |
| Sharifi (2009) | 0.42905393 | 0.41309994 | 0.44500792 |
| Al-Daghri (2010) | 0.42854956 | 0.41259843 | 0.44450065 |
| Thomas (2005) | 0.42944509 | 0.41349515 | 0.44539499 |
| Arikan (2009) | 0.42857522 | 0.41262394 | 0.44452652 |
| Guo (2010) | 0.42851931 | 0.41256878 | 0.44446984 |
| Allal-Elasmi (2010) | 0.42822063 | 0.41227573 | 0.44416553 |
| Krishnadath (2016) | 0.42782941 | 0.41189578 | 0.44376302 |
| Nezhad (2012) | 0.42906407 | 0.41311106 | 0.44501707 |
| Orces (2017) | 0.42707089 | 0.41117126 | 0.44297051 |
| Xu (2009) | 0.42867312 | 0.41272125 | 0.44462496 |
| Elasmi (2009) | 0.42811728 | 0.41217464 | 0.44405988 |
| Binh (2014) | 0.4295963 | 0.41364941 | 0.4455432 |
| Tsou (2013) | 0.42830235 | 0.41235572 | 0.44424897 |
| Lin (2007) | 0.42901281 | 0.41306028 | 0.44496536 |
| Xu (2014) | 0.42841816 | 0.4124696 | 0.44436672 |
| Deepa (2007) | 0.42844984 | 0.41250083 | 0.44439885 |
| Al-Thani (2014) | 0.42825669 | 0.4123109 | 0.44420245 |
| He (2006) | 0.4281821 | 0.41223782 | 0.44412637 |
| Oguz (2010) | 0.42785737 | 0.41192138 | 0.44379336 |
| Park (2008) | 0.42934936 | 0.41339913 | 0.44529963 |
| Bermúdez (2014) | 0.42765146 | 0.41172165 | 0.44358128 |
| RavikAsia (2010) | 0.4282811 | 0.41233489 | 0.44422734 |
| Park (2015) | 0.42864913 | 0.41269815 | 0.44460008 |
| Kosa (2015) | 0.42745662 | 0.41153356 | 0.44337967 |
| Mattsson (2007) | 0.42909512 | 0.41314328 | 0.44504696 |
| Yan (2005) | 0.42867664 | 0.4127256 | 0.44462764 |
| Miccoli (2005) | 0.42858699 | 0.41263682 | 0.44453719 |
| Soca (2016) | 0.42822835 | 0.41228306 | 0.44417366 |
| Jiang (2011) | 0.42753765 | 0.41161036 | 0.44346493 |
| Jia (2004) | 0.42918956 | 0.41323853 | 0.4451406 |
| Kim (2012) | 0.42791098 | 0.4119724 | 0.44384953 |
| Oladapo (2010) | 0.42982569 | 0.41388324 | 0.44576815 |
| Hosseini (2017) | 0.42879084 | 0.41283959 | 0.44474211 |
| Buckland (2009) | 0.42861298 | 0.41266286 | 0.44456309 |
| Qahtani (2006) | 0.42784154 | 0.41190436 | 0.44377875 |
| Welin (2008) | 0.42817509 | 0.41223067 | 0.44411951 |
| T Enkh-Oyun (2014) | 0.42756566 | 0.41163626 | 0.44349506 |
| Lakshmipriya (2013) | 0.4281038 | 0.41216063 | 0.44404694 |
| Ferguson (2010) | 0.4286702 | 0.41271988 | 0.44462052 |
| Pan (2011) | 0.42741862 | 0.41149345 | 0.44334382 |
| Parini (2016) | 0.42688555 | 0.41098133 | 0.44278973 |
| Valenzuela (2010) | 0.42833468 | 0.41238776 | 0.44428161 |
| Riediger (2011) | 0.42870614 | 0.41275579 | 0.44465649 |
| Can (2009) | 0.42773208 | 0.41179651 | 0.44366765 |
| Li (2005) | 0.42794085 | 0.41200048 | 0.44388118 |
| Gelaye (2009) | 0.42851651 | 0.41256776 | 0.44446522 |
| Bo (2005) | 0.42863977 | 0.41269016 | 0.44458938 |
| Zhao (2010) | 0.42875335 | 0.41280326 | 0.4447034 |
| Ekelund (2009) | 0.42937633 | 0.41342771 | 0.44532496 |
| Marquezine (2008) | 0.42942402 | 0.41347587 | 0.44537213 |
| Gavrila (2011) | 0.42801479 | 0.41207275 | 0.44395682 |
| Jesmin (2012) | 0.42923215 | 0.41328269 | 0.44518158 |
| Sandhofer (2007) | 0.42808241 | 0.41213918 | 0.44402564 |
| Mokan (2008) | 0.4285084 | 0.41256005 | 0.44445676 |
| Ko (2005) | 0.42932403 | 0.4133752 | 0.44527286 |
| Ko (2006) | 0.42938507 | 0.41343668 | 0.44533342 |
| Grandinetti (2005) | 0.42859656 | 0.41264758 | 0.44454551 |
| Mamun (2014) | 0.42966419 | 0.41371882 | 0.44560957 |
| Fakhrzadeh (2006) | 0.42843845 | 0.41249076 | 0.44438615 |
| Flowers (2010) | 0.4277648 | 0.41182718 | 0.4437024 |
| Santos (2004) | 0.42887649 | 0.41292667 | 0.44482633 |
| Santos (2007) | 0.42783728 | 0.4118982 | 0.4437764 |
| Pessinaba (2013) | 0.42847496 | 0.41252705 | 0.44442287 |
| Al-Lawati (2003) | 0.42910314 | 0.41315353 | 0.44505277 |
| Lawati (2006) | 0.42855614 | 0.41260764 | 0.44450465 |
| Bindraban (2008) | 0.42792618 | 0.41198531 | 0.44386703 |
| Dekker (2005) | 0.42818701 | 0.41224217 | 0.44413182 |
| Deka (2012) | 0.42787457 | 0.41193461 | 0.44381449 |
| Barros (2017) | 0.42852607 | 0.41257787 | 0.44447428 |
| Moreira (2014) | 0.42687714 | 0.4109633 | 0.44279099 |
| Alkerwi (2011) | 0.42876285 | 0.41281354 | 0.44471216 |
| Choi (2005) | 0.42790115 | 0.41196021 | 0.44384208 |
| Kelliny (2008) | 0.42801532 | 0.41207266 | 0.44395795 |
| Biad (2010) | 0.42636603 | 0.41046664 | 0.44226545 |
| Li (2010) | 0.42862552 | 0.4126772 | 0.44457388 |
| Bener (2009) | 0.42719471 | 0.41126841 | 0.44312105 |
| Salas (2014) | 0.427077 | 0.41115379 | 0.44300023 |
| Prasad (2012) | 0.42817086 | 0.4122262 | 0.44411549 |
| Rosenbaum (2005) | 0.42852125 | 0.41257358 | 0.44446892 |
| Khader (2007) | 0.42810738 | 0.41216344 | 0.4440513 |
| Esmailzadehha (2013) .428413 | 0.428413 | 0.4124662 | 0.44435981 |
| Kumbasar (2013) | 0.42899209 | 0.41304338 | 0.44494078 |
| Peer (2013) | 0.42765382 | 0.41171649 | 0.44359112 |
| Peer (2015) | 0.42765382 | 0.41171649 | 0.44359112 |
| Gupta (2004) | 0.42876869 | 0.41282022 | 0.44471717 |
| Burazor (2011) | 0.42665064 | 0.41073668 | 0.44256461 |
| Jiang (2016) | 0.42842877 | 0.41248199 | 0.44437554 |
| Misra (2010) | 0.42844087 | 0.412494 | 0.4443877 |
| Shalini (2013) | 0.42834362 | 0.4123975 | 0.44428974 |
| Owolabi (2017) | 0.42769146 | 0.41175297 | 0.44362998 |
| Al Zenki (2012) | 0.42758226 | 0.4116455 | 0.44351903 |
| Boronat (2005) | 0.42732483 | 0.41139281 | 0.44325686 |
| Ravaglia (2006) | 0.42819113 | 0.41224638 | 0.4441359 |
| Motala (2011) | 0.42825341 | 0.41230816 | 0.4441987 |
| Delavar (2009) | 0.42712915 | 0.41120067 | 0.44305763 |
| Jorgensen (2004) | 0.42862263 | 0.41267526 | 0.44457 |
| Shahbazian (2013) | 0.42890742 | 0.41295952 | 0.44485536 |
| Mora Garcia (2012) | 0.42652437 | 0.41060933 | 0.44243944 |
| Harzallah (2006) | 0.42764494 | 0.41170645 | 0.44358343 |
| Perez (2008) | 0.42817077 | 0.41222629 | 0.44411525 |
| Suarez (2008) | 0.42711574 | 0.41118628 | 0.4430452 |
| Alvi (2011) | 0.4276917 | 0.41175252 | 0.4436309 |
| Mahjoub (2012) | 0.4281469 | 0.41220263 | 0.44409117 |
| Ferguson (2010) | 0.42940563 | 0.41345924 | 0.44535199 |
| Brini (2014) | 0.42816642 | 0.41222203 | 0.44411081 |
| Zhang (2005) | 0.42741296 | 0.41147766 | 0.44334826 |
| Orhan (2011) | 0.4267261 | 0.41080412 | 0.44264808 |
| Strand (2015) | 0.42655382 | 0.4106355 | 0.4424721 |
| Gundogan (2009) | 0.42793423 | 0.41199198 | 0.44387648 |
| Franca (2016) | 0.42793897 | 0.41199666 | 0.44388127 |
| Oh (2004) | 0.42938375 | 0.41343752 | 0.44532996 |
| Houti (2016) | 0.42887518 | 0.41292793 | 0.44482243 |
| Voevoda (2016) | 0.42841178 | 0.41246593 | 0.4443576 |
| Hadaegh (2009) | 0.42772093 | 0.41178086 | 0.443661 |
| Romero (2005) | 0.42899647 | 0.41304964 | 0.44494331 |
| Sherpa (2013) | 0.42843622 | 0.41249049 | 0.44438195 |
| Cetin (2012) | 0.42830077 | 0.41235581 | 0.44424573 |
| Xavier (2010) | 0.42763138 | 0.41169208 | 0.4435707 |
| Obeidat (2015) | 0.42735019 | 0.41141424 | 0.44328615 |
| Seclen (2006) | 0.42684647 | 0.410918 | 0.44277495 |
| Son le (2005) | 0.42885974 | 0.41291344 | 0.44480604 |
| Saucedo (2009) | 0.42879421 | 0.41284797 | 0.44474041 |
| Oliveira (2011) | 0.42774394 | 0.41180333 | 0.44368452 |
| Alzahrani (2012) | 0.4291783 | 0.41323233 | 0.44512427 |
| Fontanelli (2017) | 0.42746148 | 0.41152385 | 0.4433991 |
| Schmitt (2013) | 0.42775762 | 0.41181687 | 0.44369841 |
| Tsai (2011) | 0.42962939 | 0.41368529 | 0.44557345 |
| Dhaheri (2016) | 0.42929173 | 0.41334638 | 0.4452371 |
| Zhao (2010) | 0.42797008 | 0.41202757 | 0.44391257 |
| Sawant (2011) | 0.42738572 | 0.41144872 | 0.44332272 |
| KadEuropea (2012) | 0.42814052 | 0.41219696 | 0.44408408 |
| SaEuropekonen (2012) .42863512 | 0.42863512 | 0.41268963 | 0.44458064 |
| Pimenta (2011) | 0.42952514 | 0.41358078 | 0.4454695 |
| Pimenta (2013) | 0.42952514 | 0.41358078 | 0.4454695 |
| Brutto (2013) | 0.42724594 | 0.41131037 | 0.44318151 |
| Rodriguez (2017) | 0.42696264 | 0.41103077 | 0.44289452 |
| Amarasinghe (2015) | 0.42935425 | 0.41340938 | 0.44529909 |
| Nilsson (2006) | 0.42867404 | 0.4127287 | 0.44461939 |
| Bhat (2010) | 0.42974621 | 0.41380322 | 0.4456892 |
| Khan (2015) | 0.42810482 | 0.41216218 | 0.44404745 |
|  |  |  |  |
| **Combined** | **0.42840178** | **0.41248599** | **0.44431756** |
